# Access to publicly funded weight management services in England using routine data from primary and secondary care (2007–2020): An observational cohort study

**DOI:** 10.1371/journal.pmed.1004282

**Published:** 2023-09-28

**Authors:** Karen D. Coulman, Ruta Margelyte, Tim Jones, Jane M. Blazeby, John Macleod, Amanda Owen-Smith, Helen Parretti, Richard Welbourn, Maria Theresa Redaniel, Andy Judge

**Affiliations:** 1 Health Economics Bristol, Population Health Sciences, University of Bristol, Bristol, United Kingdom; 2 National Institute for Health Research Bristol Biomedical Research Centre, Population Health Sciences, University of Bristol, Bristol, United Kingdom; 3 Centre for Academic Primary Care, Population Health Sciences, University of Bristol, Bristol, United Kingdom; 4 The National Institute for Health Research Applied Research Collaboration West (NIHR ARC West), University Hospitals Bristol and Weston NHS Foundation Trust, Bristol, United Kingdom; 5 Musculoskeletal Research Unit, Translational Health Sciences, University of Bristol, Bristol, United Kingdom; 6 Norwich Medical School, University of East Anglia, Norwich, United Kingdom; 7 Department of Upper GI and Bariatric Surgery, Somerset NHS Foundation Trust, Taunton, United Kingdom

## Abstract

**Background:**

Adults living with overweight/obesity are eligible for publicly funded weight management (WM) programmes according to national guidance. People with the most severe and complex obesity are eligible for bariatric surgery. Primary care plays a key role in identifying overweight/obesity and referring to WM interventions. This study aimed to (1) describe the primary care population in England who (a) are referred for WM interventions and (b) undergo bariatric surgery and (2) determine the patient and GP practice characteristics associated with both.

**Methods and findings:**

An observational cohort study was undertaken using routinely collected primary care data in England from the Clinical Practice Research Datalink linked with Hospital Episode Statistics. During the study period (January 2007 to June 2020), 1,811,587 adults met the inclusion criteria of a recording of overweight/obesity in primary care, of which 54.62% were female and 20.10% aged 45 to 54. Only 56,783 (3.13%) were referred to WM, and 3,701 (1.09% of those with severe and complex obesity) underwent bariatric surgery. Multivariable Poisson regression examined the associations of demographic, clinical, and regional characteristics on the likelihood of WM referral and bariatric surgery. Higher body mass index (BMI) and practice region had the strongest associations with both outcomes. People with BMI ≥40 kg/m^2^ were more than 6 times as likely to be referred for WM (10.05% of individuals) than BMI 25.0 to 29.9 kg/m^2^ (1.34%) (rate ratio (RR) 6.19, 95% confidence interval (CI) [5.99,6.40], *p* < 0.001). They were more than 5 times as likely to undergo bariatric surgery (3.98%) than BMI 35.0 to 40.0 kg/m^2^ with a comorbidity (0.53%) (RR 5.52, 95% CI [5.07,6.02], *p* < 0.001). Patients from practices in the West Midlands were the most likely to have a WM referral (5.40%) (RR 2.17, 95% CI [2.10,2.24], *p* < 0.001, compared with the North West, 2.89%), and practices from the East of England least likely (1.04%) (RR 0.43, 95% CI [0.41,0.46], *p* < 0.001, compared with North West). Patients from practices in London were the most likely to undergo bariatric surgery (2.15%), and practices in the North West the least likely (0.68%) (RR 3.29, 95% CI [2.88,3.76], *p* < 0.001, London compared with North West). Longer duration since diagnosis with severe and complex obesity (e.g., 1.67% of individuals diagnosed in 2007 versus 0.34% in 2015, RR 0.20, 95% CI [0.12,0.32], *p* < 0.001), and increasing comorbidities (e.g., 2.26% of individuals with 6+ comorbidities versus 1.39% with none (RR 8.79, 95% CI [7.16,10.79], *p* < 0.001) were also strongly associated with bariatric surgery. The main limitation is the reliance on overweight/obesity being recorded within primary care records to identify the study population.

**Conclusions:**

Between 2007 and 2020, a very small percentage of the primary care population eligible for WM referral or bariatric surgery according to national guidance received either. Higher BMI and GP practice region had the strongest associations with both. Regional inequalities may reflect differences in commissioning and provision of WM services across the country. Multi-stakeholder qualitative research is ongoing to understand the barriers to accessing WM services and potential solutions. Together with population-wide prevention strategies, improved access to WM interventions is needed to reduce obesity levels.

## Abstract 2

Using routinely collected primary and secondary care data, Karen D Coulman and colleagues investigate how access to weight management services varies across England.

## Author summary

**Why was this study done?:**

- Nearly two-thirds of adults in England live with overweight or obesity, which can affect physical, mental, and social health and well-being.
- According to national guidance, these individuals should be able to access publicly funded weight management (WM) programmes, including bariatric surgery for those where weight is having a serious impact on their health (severe and complex obesity).
- However, researchers have previously uncovered concerns that a very low percentage of people were accessing these services, and it is unknown whether this has improved in line with the growing number of people who might benefit from them.

**What did the researchers do and find?:**

- The researchers used routinely collected health data to identify 1,811,587 adults who had overweight or obesity documented in their primary care record during 2007 to 2020.
- Only 3% of these adults had a WM referral recorded during the study period. Only 1% of the 436,501 adults with severe and complex obesity underwent bariatric surgery.
- Higher body mass index and region of GP practice were the factors that were most strongly associated with receiving a WM referral and undergoing bariatric surgery.

**What do these findings mean?:**

- This study suggests that access to WM interventions in England has not improved over the last 10 years, despite obesity rates continuing to rise.
- The regional differences in access to WM programmes including bariatric surgery are important and require national attention.
- Not everyone with overweight and obesity have their weight recorded in their primary care record, and so, the true percentage receiving a WM referral may be even lower. We did not, however, identify individuals who may receive WM advice through other disease programmes.

## Introduction

Over 1.9 billion adults worldwide are living with overweight or obesity, with rates of obesity tripling since 1975 [1]. The Health Survey for England (HSE) estimates that 64% of the English adult population are living with overweight or obesity [2]. Excess weight is associated with an increased risk of type 2 diabetes mellitus (T2DM), cardiovascular disease, certain cancers, depression, reduced quality of life, and premature death [3–8]. Obesity is reported to cost the NHS £6.5 billion annually, with the full annual cost of obesity in the United Kingdom around £58 billion [9]. While effective public health initiatives are necessary to prevent future overweight and obesity, these are not sufficient to achieve weight loss in those that are already living with obesity, particularly those with severe and complex obesity who are at the highest risk of morbidity and premature death [2,10,11]. Effective clinical interventions are needed for those already living with obesity, to reduce associated morbidity and healthcare costs [10].

In England, the National Institute for Health and Care Excellence (NICE) recommends publicly funded community weight management (WM) services for people with a body mass index (BMI) ≥30 kg/m^2^ (or lower for people from minority ethnic groups who are at an increased risk of developing weight-related comorbidities), and where there is capacity, access should not be restricted for those with BMI 25.0 to 29.9 kg/m^2^ (or lower for people from ethnic minority groups) [12]. Community WM services can include group-based and/or individual interventions delivered by community-based professionals to reduce energy intake and encourage physical activity [12,13]. Specialist WM services are available for people with severe and complex obesity, which adopt a multidisciplinary health professional approach to WM [14,15]. From here, consideration for bariatric surgery can also be made [14]. NICE recommends that bariatric surgery is considered as a cost-effective potential intervention for people with severe and complex obesity, unable to achieve adequate or sustained weight loss through nonsurgical interventions alone [11,16,17].

The number of referrals made to WM services is not known, although the National Obesity Audit was launched in April 2022 with the aim of improving understanding of access to and outcomes of WM programmes in England [18,19]. Access to healthcare has been theorised as a multidimensional concept reflecting the availability, utilisation, and outcomes of services [20]. Previous research undertaken using anonymised primary care records from the Clinical Practice Research Datalink (CPRD; records from around 7% of GP practices in England) between 2005 and 2012 reported low access to primary care WM interventions in terms of service utilisation, which included brief advice from general practitioners (GPs), prescriptions for WM drugs, and referrals to WM services [21,22]. Approximately 60% of people with a BMI ≥40 kg/m^2^ did not have any WM intervention recorded during the study period, and this rose to 90% in those with a BMI 25 to 29.9 kg/m^2^. In terms of WM referrals specifically, only around 17% of people with a BMI ≥40 kg/m^2^ received a referral, dropping to 3% in those with a BMI 25 to 29.9 kg/m^2^. Reasons for the low rates of WM interventions may include lack of consistency in the availability of WM services and access criteria across the country [23]. It is not known whether intervention rates have improved in the last 10 years. A recent systematic review investigated inequalities in the uptake, adherence to and effectiveness of behavioural WM interventions within trial settings, and found that most trials did not examine whether inequalities occurred [24]. To our knowledge, no systematic review has investigated inequalities in WM referrals within routine clinical practice.

Similarly, NHS bariatric surgery rates in the UK are low compared with other European countries and are estimated to be far below clinical need [25–27]. Rates and clinical outcomes of bariatric surgery have previously been investigated in CPRD studies; however, to our knowledge, there are no data on patient and practice characteristics associated with likelihood of undergoing bariatric surgery within the eligible primary care population in England [28–31].

The aims of this study were to (1) describe the population of adults with a recording of overweight or obesity in primary care in England, including those who (a) are referred for a publicly funded WM intervention from primary care and (b) undergo NHS bariatric surgery, and (2) determine the patient and GP practice characteristics associated with (a) WM referral and (b) bariatric surgery. In this paper, we focus on the service utilisation aspect of access.

## Methods

### Ethics statement

The study protocol was approved by the Independent Scientific Advisory Committee (ISAC, now CPRD Expert Review Committees; protocol number: 20_138). All data used in this study are routinely collected and anonymised, and, thus, consent is not required. The use of CPRD in research has ethics approval from the Health Research Authority (https://cprd.com/safeguarding-patient-data).

### Study design

An observational cohort study design was undertaken using primary care data from CPRD of all adult patients in England with a record indicating overweight or obesity. The study period ran from January 2007 (to coincide with the publication of the first NICE guidance for obesity in December 2006) to June 2020 (the latest CPRD update available when the data were extracted). The CPRD-approved protocol with the statistical analysis plan is available in **S1 Appendix.** Our protocol contained 4 specific aims; this paper reports the first 2 aims, a separate publication will focus on the trends in bariatric surgery before and after April 2017 using interrupted time series analysis. Our research questions were as follows: (1) Of people with overweight and obesity in primary care in England (eligible population), who and how many are (a) referred for WM and (b) undergo publicly funded bariatric surgery (in the subgroup with severe and complex obesity)? (2) For people who are eligible, which patient and GP-practice factors are associated with WM referral and bariatric surgery? This study is reported as per the Strengthening the Reporting of Observational Studies in Epidemiology (STROBE) guideline for observational studies using routinely collected data (**S1 Checklist**) [32].

### Data sources

This study was carried out using anonymised primary care data from CPRD GOLD. At the time of data extraction (June 2020), CPRD GOLD included records from 918 general practices in the UK, and 21,506,368 patients. The demographics of registered patients within CPRD have been reported to be representative of the UK [22]. CPRD data are validated, audited, and quality checked [33]. Primary care data from CPRD GOLD were linked to the Hospital Episode Statistics–Admitted Patient Care (HES-APC) data, and the Index of Multiple Deprivation (IMD, patient level), where patients were eligible for linkage (76% of patients in the current study). Data from the 2011 Rural–Urban classification were also linked at the general practice level. All data were extracted and cleaned by members of the CPRD research team (TJ and RM) at the University of Bristol, which held a CPRD license, looking at completeness and consistency of the data. Linkages were undertaken by the CPRD data team.

### Participants and inclusion criteria

The source population included all patients with “research quality” data (deemed “acceptable” quality by CPRD and providing some “up-to-standard” data), registered with general practices between January 2007 and June 2020 contributing to the CPRD GOLD primary care dataset [22]. Adults aged ≥18 years who were registered at GP practices in England were included in the study if during the study period they had a recording of overweight or obesity (BMI ≥25.0 or ≥23.0 kg/m^2^ in people of Black and Asian ethnic groups as defined in the 2014 NICE obesity guidance) in CPRD GOLD [11] (**Table 1**). To identify the first recording of obesity within primary care, we only included individuals who had ≥365 days of up-to-standard registration with the practice (based on when the practice’s data were classed as “research quality” by CPRD) prior to their recording of overweight/obesity to be included in the study. Individuals who had a recording of bariatric surgery in CPRD GOLD or HES-APC before their index date (earliest eligible recording of overweight/obesity) were excluded. For analyses with bariatric surgery as the outcome, the study population was restricted to individuals with severe and complex obesity as defined in **Table 1** who were eligible for bariatric surgery according to NICE guidance [11]. The earliest eligible recording of severe and complex obesity was the “severe and complex obesity index date.” In addition to weight, height, and BMI recordings in CPRD, relevant obesity medical (READ) and product codes (obesity medications) within CPRD were used to define overweight or obesity and severe and complex obesity (study population) (**Table 1**).

**Table 1:** Exposure definitions^a^.

Definition of overweight or obesity	Definition of severe and complex obesity^b^
A recorded BMI ≥25.0 kg/m^2^ in CPRD GOLD **OR**	A recorded BMI ≥40.0 kg/m^2^ in CPRD GOLD **OR**
A recorded BMI ≥23.0 kg/m^2^ in CPRD GOLD in people of Black African, African-Caribbean, and Asian (B&A) ethnic groups^c^ **OR**	A recorded BMI 35.0–39.9 kg/m^2^ in CPRD GOLD **AND** one of the following recorded comorbidities^d^: • T2DM • Hypertension • CHD • OSA • Asthma • Chronic musculoskeletal condition (e.g., osteoarthritis, back pain, knee pain, excluding those with inflammatory conditions only) • GORD • Liver disease • PCOS • Fertility problems • Depression • Anxiety • IIH **OR**
A clinical code in CPRD GOLD indicating a diagnosis of overweight or obesity **OR**	A recorded BMI 30.0–34.9 kg/m^2^ in CPRD GOLD **AND** T2DM diagnosed no more than 10 years prior to first eligible BMI measurement **OR**
A clinical code in CPRD GOLD indicating a prescription for obesity drugs	A recorded BMI 27.5–30.0 kg/m^2^ CPRD GOLD in people from B&A groups^c^ **AND** T2DM diagnosed no more than 10 years prior to first eligible BMI measurement **OR**
	A clinical code in CPRD GOLD indicating a diagnosis of severe obesity

^a^All code lists available in S2 and S3 Appendices. Ethnicity and comorbidities were defined using relevant codes from both CPRD GOLD and HES-APC where available.

^b^The date of the earliest recorded event, which matches these criteria, was the “severe and complex obesity index date.” For points 2, 3, and 4, the date at which a patient is first diagnosed with a relevant comorbidity after their eligible BMI recording is their index date. If a relevant comorbidity was diagnosed prior to their eligible BMI recording, the index date is the date of first eligible BMI recording.

^c^People of “Mixed” Black or Asian ethnicity were also included within these groups. In people with “Unknown” ethnicity stated, the BMI thresholds for the White population were used.

^d^Weight-related comorbidities were included as per the National Bariatric Surgery Registry and discussion with the study team including expert obesity health professionals.

BMI, body mass index; CHD, coronary heart disease; CPRD, Clinical Practice Research Datalink; GORD, gastro-oesophageal reflux disease; HES-APC, Hospital Episode Statistics–Admitted Patient Care; IIH, idiopathic intracranial hypertension; OSA, obstructive sleep apnoea; PCOS, polycystic ovary syndrome; T2DM, type 2 diabetes mellitus.

### Outcomes

Relevant clinical codes were used to define whether an individual was referred for a WM intervention (YES/NO) (outcome A). We were not able to differentiate between types of WM referrals (e.g., community-based or specialist WM services) from the available codes. NHS bariatric surgery (YES/NO) (outcome B) was identified using the International Classification of Diseases (version 10) (ICD-10) diagnostic codes for obesity and OPCS Classification of Interventions and Procedures (OPCS-4) codes for bariatric surgery in HES-APC. KC (obesity specialist dietitian) compiled the code lists for the exposures and outcomes, which were cross-checked by HP (GP with specialist interest in obesity). Previously published bariatric surgery procedure codes (cross-checked by RW, bariatric surgeon) were used to identify bariatric surgery [34]. Bariatric surgery as recorded in CPRD was not used to define the outcome due to the inclusion of private bariatric surgery within primary care records. Code lists used to define exposures and outcomes are available in **S2 Appendix**.

### Covariables

Co-ariables included the following patient-level variables: sex, age group at diagnosis with overweight/obesity (or severe and complex obesity), ethnicity (identified using both clinical codes within CPRD and patient information within HES mapped into six ethnicity categories as per Mathur and colleagues [35]), IMD (categorised into quintiles with 1 being the least deprived and 5 being the most deprived group), smoking status (earliest after index date, not censored for outcome; identified from clinical codes and Additional Clinical Details file in CPRD and classified into current, ex-, and never/nonsmoker), BMI category (as defined in the 2014 NICE obesity guidance [11]) at diagnosis with overweight/obesity (or severe and complex obesity), year of diagnosis with overweight/obesity (or severe and complex obesity), and the 13 weight-related comorbidities specified in **Table 1**. Comorbidities were included in the analysis models in 2 different ways; as presence of individual comorbidities (13 individual binary YES/NO comorbidity variables, model A) and as a total number of comorbidities (a single categorical variable, model B). Both clinical codes within CPRD and ICD-10 codes within HES were used to identify comorbidities where possible to improve completeness of data. Existing publicly available code lists were used where possible. Code lists used to define covariables are available in **S3 Appendix**. General practice–level variables included region (Strategic Health Authority) of England and Rural–Urban classification (classified as either “rural” or “urban”) based on practice postcode.

### Follow-up

Individuals included in the study were followed up from first indication of overweight/obesity until the earliest of the following: first indication of WM referral or bariatric surgery, death, transfer out from practice; end of practice data collection; or end of the study period (June 2020). Date of death was taken from CPRD GOLD.

### Statistical analyses

Descriptive statistics were used to describe the characteristics of adults living with overweight and obesity in England since 2007 (the publication of the first NICE guidance for obesity), including those who were referred for WM and/or underwent bariatric surgery. Multivariable Poisson regression was used to determine the association between patient and general practice characteristics (as specified in “Covariables”) and (a) having a WM referral and (b) undergoing bariatric surgery. Analyses with bariatric surgery as outcome were restricted to the subgroup of the study population with severe and complex obesity. To allow for adequate time for an outcome to occur, regression analyses were restricted to individuals with at least 2- and 5-year follow-up data for WM referral and bariatric surgery, respectively, based on examining the distribution of outcome occurrence after index date using kernel density plots. Individuals that had an “unacceptable” data quality indicator during the study period as assigned by CPRD (e.g., data were not deemed to be of research quality) were not included in the regression models. The Poisson regression calculated effect sizes (reported as both crude and adjusted rate ratios (RRs)), 95% confidence intervals (CIs), and Wald *p*-values.

Some data were missing for IMD, smoking, and Rural–Urban index of GP practice. Additionally, for ethnicity, 16.5% of individuals included in the study were coded as “Unknown” ethnicity. Missing or “Unknown” data for these covariables were not imputed as they are probably not missing at random [36,37]. The characteristics of individuals included in the full model versus those with complete data across all variables included in the adjusted model were compared to examine the potential for information and confounding bias. We also conducted a sensitivity analysis removing people with “Unknown” ethnicity from the adjusted models. All statistical analyses were conducted using Stata/MP version 16.1.

### Patient and public involvement

This study is part of a wider project to investigate barriers to accessing WM services, which has established a patient and public involvement (PPI) advisory group consisting of people living with obesity and bariatric surgery. The PPI advisory group specifically provided input on which covariables to collect within this study and will help with dissemination of the study findings.

## Results

### Participants and descriptive information

**Fig 1:**
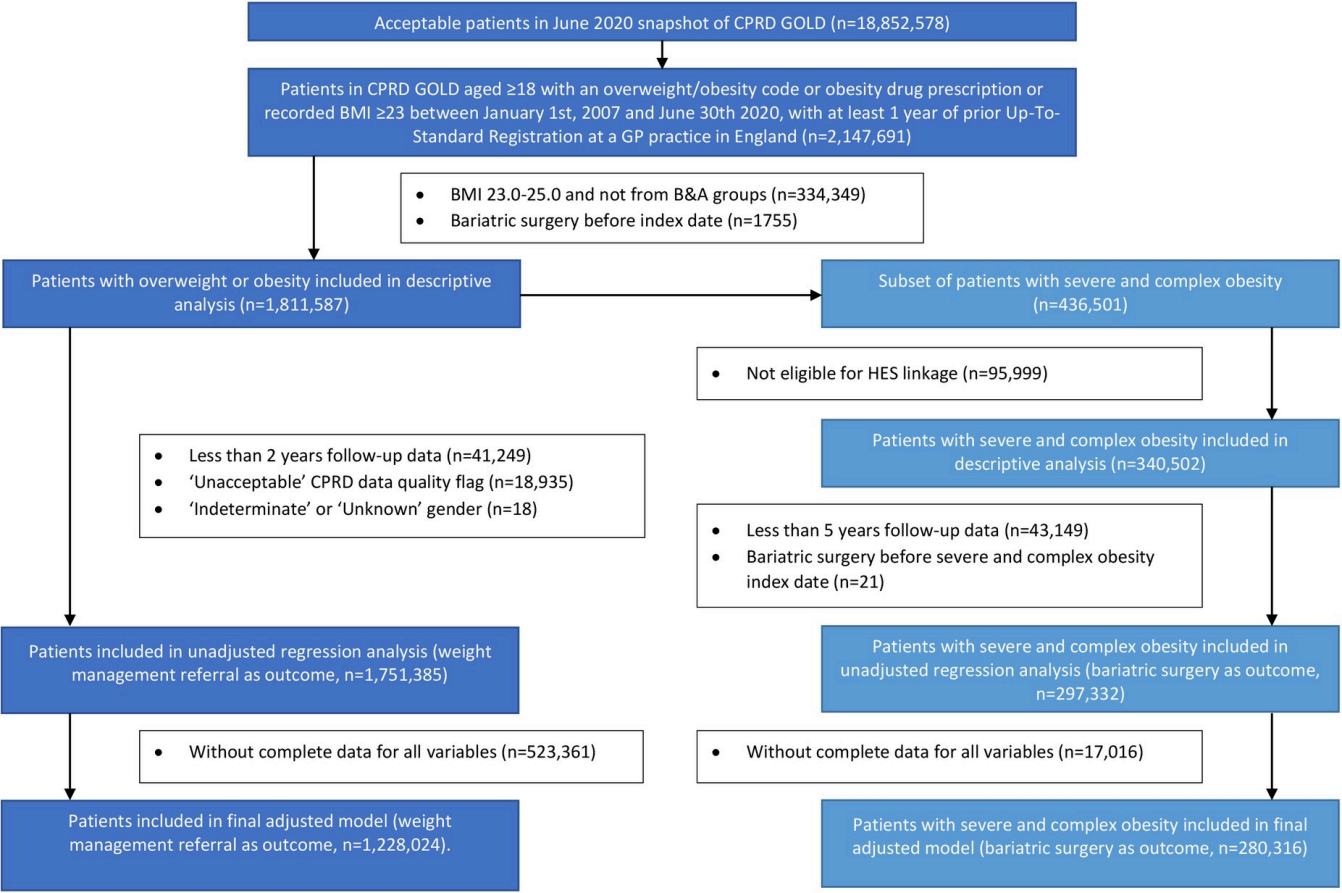
Study flow diagram. BMI, body mass index; B&A, Black and Asian ethnic groups; HES, Hospital Episode Statistics.

A total of 1,811,587 adults met the study inclusion criteria of having a recording of overweight or obesity during the study period (**Fig 1**), representing 31.09% of the 5,827,309 adults with at least 365 days of practice registration in CPRD in England. Of those with a recording of overweight or obesity (1,811,587), 989,432 (54.62%) were female, 364,084 (20.10%) were aged 45 to 54, and 268,522 (14.82%) had a recording of T2DM (**S1 Table**). Of adults in CPRD in England in 2007 (3,503,814), 15.33% (537,266) had a recording of overweight or obesity (**S1 Fig**). This increased throughout each study year until 2015, peaking at 38.33% (884,243 out of 2,306,665). Thereafter, it gradually dropped (35.90% (238,131 out of 663,328) in 2020).

### Weight management referrals

The median study follow-up time from date of diagnosis with overweight/obesity (index date) was 7.18 years (interquartile range (IQR) 3.47 to 10.70 years). During the study period, 56,783/1,811,587 adults with overweight or obesity had a recorded WM referral equating to just 3.13% of adults living with overweight or obesity within the study (**Table 2**). The percentage with a WM referral was highest in adults with a BMI ≥40 kg/m^2^ (8,685/87,031, 9.98%), and adults living with overweight or obesity with a medical code for whom BMI category was not available (4,131/25,904, 15.95%). It was lowest in adults from Black and Asian ethnic groups with BMI 23.0 to 24.9 kg/m^2^ (209/30,335, 0.69%). Adults from GP practices in the West Midlands had the highest percentage of WM referrals recorded (13,235/249,143, 5.31%), whereas the East of England had the lowest (1,860/172,177, 1.08%). In 2007, 0.38% (2,067/537,266) of adults active in the study that year had a WM referral recorded; this increased over time to 1.47% (4,886/333,272) of adults active in the study in 2019 having a WM referral that year (**S2 Fig**).

**Table 2:** Characteristics of adults with overweight and obesity in England with a WM referral recorded in CPRD GOLD, by BMI group (all years 2007–2020).

	BMI (kg/m^2^) group at diagnosis with overweight/obesity						
	23.0–24.9 (B&A only)	25.0–29.9	30.0–34.9	35.0–40.0	40.0 +	Medical codes^b^	All
	**n (%** ^**a**^ **)**	**n (%** ^**a**^ **)**	**n (%** ^**a**^ **)**	**n (%** ^**a**^ **)**	**n (%** ^**a**^ **)**	**n (%** ^**a**^ **)**	**n (%** ^**c**^ **)**
**Total**	209 (0.37)	15,349 (27.03)	18,068 (31.82)	10,341 (18.21)	8,685 (15.30)	4,131 (7.28)	56,783
***Sex***							
Male	74 (0.38)	6,054 (31.00)	6,618 (33.89)	3,121 (15.98)	2,310 (11.83)	1,353 (6.93)	19,530 (34.39)
Female	135 (0.36)	9,294 (24.95)	11,449 (30.73)	7,220 (19.38)	6,375 (17.11)	2,778 (7.46)	37,251 (65.60)
Indeterminate/Unknown	0 (0.00)	1 (50.00)	1 (50.00)	0 (0.00)	0 (0.00)	0 (0.00)	2 (0.00)
***Age group at WM referral***							
18–24	1 (0.06)	279 (16.84)	491 (29.63)	372 (22.45)	369 (22.27)	145 (8.75)	1,657 (2.92)
25–34	12 (0.27)	675 (15.36)	1,159 (26.37)	1,080 (24.57)	1,160 (26.39)	309 (7.03)	4,395 (7.74)
35–44	56 (0.58)	2,269 (23.39)	2,853 (29.41)	1,861 (19.18)	1,885 (19.43)	778 (8.02)	9,702 (17.09)
45–54	83 (0.52)	4,279 (26.98)	4,999 (31.52)	2,830 (17.84)	2,475 (15.61)	1,193 (7.52)	15,859 (27.93)
55–64	42 (0.30)	4,171 (29.43)	4,674 (32.98)	2,468 (17.41)	1,834 (12.94)	984 (6.94)	14,173 (24.96)
65–74	15 (0.16)	3,238 (34.51)	3,235 (34.47)	1,469 (15.65)	841 (8.96)	586 (6.24)	9,384 (16.53)
75+	0 (0.00)	438 (27.15)	657 (40.73)	261 (16.18)	121 (7.50)	136 (8.43)	1,613 (2.84)
***Ethnic group***							
White	0 (0.00)	12,081 (26.91)	14,245 (31.73)	8,273 (18.43)	6,985 (15.56)	3,307 (7.37)	44,891 (79.06)
Asian	125 (6.70)	605 (32.40)	642 (34.39)	264 (14.14)	150 (8.03)	81 (4.34)	1,867 (3.29)
Black	57 (3.54)	343 (21.32)	563 (34.99)	318 (19.76)	265 (16.47)	63 (3.92)	1,609 (2.83)
Mixed	27 (6.80)	117 (29.47)	123 (30.98)	54 (13.60)	61 (15.37)	15 (3.78)	397 (0.70)
Other	0 (0.00)	197 (31.02)	213 (33.54)	89 (14.02)	74 (11.65)	62 (9.76)	635 (1.12)
Unknown	0 (0.00)	2,006 (27.17)	2,282 (30.90)	1,343 (18.19)	1,150 (15.57)	603 (8.17)	7,384 (13.00)
***IMD***							
1 (least deprived)	18 (0.24)	2,467 (32.40)	2,618 (34.38)	1,261 (16.56)	860 (11.29)	390 (5.12)	7,614 (13.41)
2	31 (0.35)	2,751 (30.85)	2,891 (32.42)	1,426 (15.99)	1,057 (11.85)	761 (8.53)	8,917 (15.70)
3	24 (0.32)	2,081 (27.43)	2,381 (31.38)	1,381 (18.20)	1,112 (14.66)	608 (8.01)	7,587 (13.36)
4	27 (0.33)	1,841 (22.61)	2,645 (32.49)	1,615 (19.84)	1,487 (18.26)	527 (6.47)	8,142 (14.34)
5 (most deprived)	32 (0.36)	1,657 (18.63)	2,666 (29.97)	1,975 (22.20)	1,946 (21.88)	620 (6.97)	8,896 (15.67)
*Missing*	77 (0.49)	4,552 (29.13)	4,867 (31.14)	2,683 (17.17)	2,223 (14.23)	1,225 (7.84)	15,627 (27.52)
***Smoking status***							
Current smoker	21 (0.18)	3,238 (27.78)	3,376 (28.96)	2,019 (17.32)	1,759 (15.09)	1,243 (10.66)	11,656 (20.53)
Ex-smoker	27 (0.18)	3,909 (26.54)	4,995 (33.92)	2,815 (19.11)	2,129 (14.46)	852 (5.79)	14,727 (25.94)
Never/nonsmoker	159 (0.56)	8,004 (28.20)	9,165 (32.29)	5,093 (17.94)	4,338 (15.28)	1,626 (5.73)	28,385 (49.99)
*Missing*	2 (0.10)	198 (9.83)	532 (26.40)	414 (20.55)	459 (22.78)	410 (20.35)	2,015 (3.55)
***Presence of comorbidities***							
Type 2 diabetes	12 (0.13)	959 (10.50)	2,537 (27.78)	2,308 (25.27)	2,757 (30.19)	560 (6.13)	9,133 (16.08)
Hypertension	17 (0.13)	2,128 (16.11)	4,162 (31.51)	3,035 (22.98)	3,009 (22.78)	857 (6.49)	13,208 (23.26)
Coronary heart disease	4 (0.09)	858 (18.96)	1,508 (33.33)	1,004 (22.19)	840 (18.56)	311 (6.87)	4,525 (7.97)
Obstructive sleep apnoea	0 (0.00)	195 (8.26)	476 (20.17)	517 (21.91)	958 (40.59)	214 (9.07)	2,360 (4.16)
Asthma	23 (0.23)	2,073 (20.55)	3,000 (29.74)	2,133 (21.14)	2,143 (21.24)	716 (7.10)	10,088 (17.77)
Chronic musculoskeletal condition	19 (0.14)	2,904 (21.19)	4,428 (32.30)	2,871 (20.95)	2,546 (18.57)	939 (6.85)	13,707 (24.14)
Gastro-oesophageal reflux disease	57 (0.32)	4,582 (25.54)	5,800 (32.33)	3,454 (19.26)	2,788 (15.54)	1,257 (7.01)	17,938 (31.59)
Liver disease	1 (0.07)	209 (15.48)	375 (27.78)	300 (22.22)	347 (25.70)	118 (8.74)	1,350 (2.38)
Polycystic ovarian syndrome	5 (0.30)	181 (10.95)	382 (23.11)	400 (24.20)	545 (32.97)	140 (8.47)	1,653 (2.91)
Fertility problems	11 (0.59)	421 (22.66)	557 (29.98)	370 (19.91)	359 (19.32)	140 (7.53)	1,858 (3.27)
Depression	36 (0.18)	4,517 (22.60)	5,971 (29.87)	3,995 (19.98)	3,980 (19.91)	1,492 (7.46)	19,991 (35.21)
Anxiety	32 (0.23)	3,248 (23.76)	4,208 (30.78)	2,658 (19.44)	2,471 (18.07)	1,054 (7.71)	13,671 (24.08)
Idiopathic intracranial hypertension	0 (0.00)	11 (7.38)	28 (18.79)	34 (22.82)	63 (42.28)	13 (8.72)	149 (0.26)
***Strategic Health Authority of GP practice***							
North East	1 (0.07)	258 (18.42)	455 (32.48)	312 (22.27)	293 (20.91)	82 (5.85)	1,401 (2.47)
North West	11 (0.14)	1,640 (20.23)	2,389 (29.46)	1,751 (21.60)	1,688 (20.82)	629 (7.76)	8,108 (14.28)
Yorkshire and the Humber	0 (0.00)	191 (25.10)	208 (27.33)	159 (20.89)	167 (21.94)	36 (4.73)	761 (1.34)
East Midlands	1 (0.05)	215 (11.70)	665 (36.20)	481 (26.18)	357 (19.43)	118 (6.42)	1,837 (3.24)
West Midlands	54 (0.41)	3,901 (29.47)	4,205 (31.77)	2,046 (15.46)	1,504 (11.36)	1,525 (11.52)	13,235 (23.31)
East of England	1 (0.05)	318 (17.10)	603 (32.42)	417 (22.42)	408 (21.94)	113 (6.08)	1,860 (3.28)
South West	3 (0.06)	722 (15.44)	1,523 (32.56)	1,133 (24.22)	1,039 (22.22)	257 (5.49)	4,677 (8.24)
South Central	5 (0.08)	1,032 (15.56)	2,439 (36.77)	1,526 (23.00)	1,245 (18.77)	387 (5.83)	6,634 (11.68)
London	56 (0.94)	1,429 (23.88)	2,017 (33.71)	1,107 (18.50)	957 (16.00)	417 (6.97)	5,983 (10.54)
South East Coast	77 (0.63)	5,643 (45.93)	3,564 (29.01)	1,409 (11.47)	1,027 (8.36)	567 (4.61)	12,287 (21.64)
***Rural–urban classification of GP practice***							
Rural	4 (0.08)	1,659 (33.20)	1,692 (33.86)	785 (15.71)	573 (11.47)	284 (5.68)	4,997 (8.80)
Urban	135 (0.36)	9,381 (25.31)	11,783 (31.79)	7,050 (19.02)	6,042 (16.30)	2,670 (7.20)	37,061 (65.27)
*Missing*	70 (0.48)	4,309 (29.26)	4,593 (31.19)	2,506 (17.02)	2,070 (14.06)	1,177 (7.99)	14,725 (25.93)

BMI, body mass index; B&A, Black and Asian ethnic groups; CPRD, Clinical Practice Research Datalink; GP, general practitioner; IMD, Index of Multiple Deprivation; WM, weight management.

^a^Percentage of the total variable.

^b^Medical codes indicating diagnosis with overweight or obesity where BMI category not specified.

^c^Percentage of “All” (*n =* 56,783).

### Bariatric surgery

During the study period, 436,501 (7.49%) adults had a recording of severe and complex obesity (as defined by 2014 NICE guidance) out of 5,827,309 adults with at least 365 days of practice registration. The median follow-up time from severe and complex obesity index date was 6.85 years (IQR 3.28 to 10.51). Of the 340,502 eligible for HES linkage, 3,701 (1.09%) underwent NHS bariatric surgery (**Table 3**). The percentage undergoing bariatric surgery was highest in adults with a BMI ≥40 kg/m^2^ (2,872/78,406, 3.66%) and lowest in adults with BMI 27.5 to 29.9 kg/m^2^ from Black and Asian ethnic groups with T2DM diagnosed in the last 10 years (9/41,946, 0.02%). The highest percentage of bariatric surgery was in adults from GP practices in the North East (193/8,585, 2.25%), with the lowest in the North West (367/59,721, 0.61%), where there were 5 and 3 NHS bariatric centres, respectively. A full list of current NHS bariatric surgery centres in England provided by the British and Metabolic Surgery Society is available in **S4 Appendix.** Of adults with severe and complex obesity active in the study in 2007, 0.06% (62/99,445) underwent bariatric surgery that year; this was highest in 2018 with 0.51% (355/70,236) of adults active in the study this year undergoing bariatric surgery (**S3 Fig**).

**Table 3:** Characteristics of adults with severe and complex obesity in CPRD GOLD undergoing bariatric surgery in England, by BMI group (all years 2007–2020).

BMI (kg/m^2^) group at diagnosis with severe and complex obesity							
	27.5–29.9 (B&A only with T2DM in last 10 years) n (%^a^)	30.0–34.9 with T2DM in last 10 years n (%^a^)	35.0–39.9 + comorbidity n (%^a^)	40.0 + n (%^a^)	Medical codes^b^ n (%^a^)	All n (%^c^)	
**Total**	9 (0.24)	57 (1.54)	757 (20.45)	2,872 (77.60)	6 (0.16)	3,701	
***Sex***							
Male	3 (0.34)	25 (2.86)	151 (17.26)	694 (79.31)	2 (0.23)	875 (23.64)	
Female	6 (0.21)	32 (1.13)	606 (21.44)	2,178 (77.07)	4 (0.14)	2,826 (76.36)	
***Age group at bariatric surgery***							
18–24	0 (0.00)	0 (0.00)	7 (13.21)	46 (86.79)	0 (0.00)	53 (1.43)	
25–34	0 (0.00)	1 (0.19)	111 (21.31)	407 (78.12)	2 (0.38)	521 (14.08)	
35–44	1 (0.11)	4 (0.43)	183 (19.89)	731 (79.46)	1 (0.11)	920 (24.86)	
45–54	3 (0.23)	20 (1.56)	268 (20.86)	992 (77.20)	2 (0.16)	1,285 (34.72)	
55–64	1 (0.14)	15 (2.05)	139 (18.96)	577 (78.72)	1 (0.14)	733 (19.81)	
65–74	2 (1.16)	14 (8.09)	44 (25.43)	113 (65.32)	0 (0.00)	173 (4.67)	
75+	2 (12.50)	3 (18.75)	5 (31.25)	6 (37.50)	0 (0.00)	16 (0.43)	
***Ethnic group***							
White	5 (0.15)	47 (1.40)	654 (19.52)	2,641 (78.81)	4 (0.12)	3,351 (90.54)	
Asian	1 (1.08)	4 (4.30)	26 (27.96)	62 (66.67)	0 (0.00)	93 (2.51)	
Black	3 (2.38)	4 (3.17)	38 (30.16)	80 (63.49)	1 (0.79)	126 (3.40)	
Mixed	0 (0.00)	1 (2.38)	13 (30.95)	28 (66.67)	0 (0.00)	42 (1.13)	
Other	0 (0.00)	1 (1.61)	19 (30.65)	42 (67.74)	0 (0.00)	62 (1.68)	
Unknown	0 (0.00)	0 (0.00)	7 (25.93)	19 (70.37)	1 (3.70)	27 (0.73)	
***IMD***							
1 (least deprived)	2 (0.45)	7 (1.56)	105 (23.39)	335 (74.61)	0 (0.00)	449 (12.13)	
2	2 (0.31)	15 (2.30)	142 (21.75)	493 (75.50)	1 (0.15)	653 (17.64)	
3	2 (0.26)	9 (1.18)	155 (20.39)	593 (78.03)	1 (0.13)	760 (20.53)	
4	0 (0.00)	13 (1.47)	166 (18.74)	705 (79.57)	2 (0.23)	886 (23.94)	
5 (most deprived)	3 (0.32)	13 (1.37)	189 (19.85)	745 (78.26)	2 (0.21)	952 (25.72)	
*Missing*	0 (0.00)	0 (0.00)	0 (0.00)	1 (100.00)	0 (0.00)	1 (0.03)	
***Smoking status***							
Current smoker	1 (0.13)	17 (2.17)	193 (24.65)	572 (73.05)	0 (0.00)	783 (21.16)	
Ex-smoker	2 (0.20)	17 (1.72)	219 (22.12)	750 (75.76)	2 (0.20)	990 (26.75)	
Never/nonsmoker	6 (0.37)	22 (1.36)	294 (18.22)	1,289 (79.86)	3 (0.19)	1,614 (43.61)	
*Missing*	0 (0.00)	1 (0.32)	51 (16.24)	261 (83.12)	1 (0.32)	314 (8.48)	
***Presence of comorbidities***							
Type 2 diabetes	9 (0.72)	57 (4.57)	208 (16.68)	971 (77.87)	2 (0.16)	1,247 (33.69)	
Hypertension	4 (0.34)	25 (2.12)	206 (17.47)	941 (79.81)	3 (0.25)	1,179 (31.86)	
Coronary heart disease	0 (0.00)	20 (5.38)	72 (19.35)	280 (75.27)	0 (0.00)	372 (10.05)	
Obstructive sleep apnoea	1 (0.16)	2 (0.33)	89 (14.61)	517 (84.89)	0 (0.00)	609 (16.46)	
Asthma	2 (0.17)	21 (1.76)	276 (23.17)	889 (74.64)	3 (0.25)	1,191 (32.18)	
Chronic musculoskeletal condition	2 (0.16)	21 (1.63)	255 (19.80)	1,009 (78.34)	1 (0.08)	1,288 (34.80)	
Gastro-oesophageal reflux disease	5 (0.31)	21 (1.30)	401 (24.81)	1,187 (73.45)	2 (0.12)	1,616 (43.66)	
Liver disease	1 (0.24)	13 (3.08)	93 (22.04)	314 (74.41)	1 (0.24)	422 (11.40)	
Polycystic ovarian syndrome	0 (0.00)	3 (0.74)	87 (21.53)	314 (77.72)	0 (0.00)	404 (10.92)	
Fertility problems	1 (0.43)	1 (0.43)	61 (26.18)	170 (72.96)	0 (0.00)	233 (6.30)	
Depression	3 (0.14)	28 (1.26)	519 (23.41)	1,664 (75.06)	3 (0.14)	2,217 (59.90)	
Anxiety	3 (0.24)	17 (1.36)	293 (23.48)	934 (74.84)	1 (0.08)	1,248 (33.72)	
Idiopathic intracranial hypertension	0 (0.00)	1 (2.13)	9 (19.15)	37 (78.72)	0 (0.00)	47 (1.27)	
***Strategic Health Authority of GP practice***							**Number of NHS bariatric surgery centres per region** ^**d**^
North East	2 (1.04)	0 (0.00)	45 (23.32)	146 (75.65)	0 (0.00)	193 (5.21)	5
North West	1 (0.27)	8 (2.18)	72 (19.62)	286 (77.93)	0 (0.00)	367 (9.92)	3
Yorkshire and the Humber	0 (0.00)	2 (1.19)	21 (12.50)	145 (86.31)	0 (0.00)	168 (4.54)	7
East Midlands	0 (0.00)	1 (0.96)	18 (17.31)	85 (81.73)	0 (0.00)	104 (2.81)	2
West Midlands	0 (0.00)	7 (1.76)	77 (19.40)	312 (78.59)	1 (0.25)	397 (10.73)	5
East of England	2 (0.76)	5 (1.89)	46 (17.42)	211 (79.92)	0 (0.00)	264 (7.13)	1
South West	0 (0.00)	7 (1.42)	97 (19.72)	387 (78.66)	1 (0.20)	492 (13.29)	6
South Central	1 (0.23)	5 (1.15)	92 (21.15)	336 (77.24)	1 (0.23)	435 (11.75)	3
London	3 (0.43)	16 (2.30)	183 (26.33)	492 (70.79)	1 (0.14)	695 (18.78)	8
South East Coast	0 (0.00)	6 (1.02)	106 (18.09)	472 (80.55)	2 (0.34)	586 (15.83)	3
***Rural–urban classification of GP practice***							
Rural	1 (0.24)	5 (1.20)	77 (18.47)	332 (79.62)	2 (0.48)	417 (11.27)	
Urban	8 (0.24)	52 (1.58)	680 (20.71)	2,540 (77.34)	4 (0.12)	3,284 (88.73)	

BMI, body mass index; B&A, Black and Asian ethnic groups; CPRD, Clinical Practice Research Datalink; GP, general practitioner; IMD, Index of Multiple Deprivation; NHS, National Health Service.

^a^Percentage of the total variable.

^b^Medical codes indicating diagnosis with severe and complex obesity where BMI category not specified.

^c^Percentage of “All” (*n* = 3,701).

^d^See S4 Appendix for the list of NHS bariatric surgery centres by region.

### Poisson analyses

#### Weight management referrals

A total of 1,751,385 patients with overweight or obesity had at least 2 years of follow-up and “research quality” data over the study period (96.68% of full sample of 1,811,587) and were eligible to be included in the regression analysis (**Fig 1 and Table 4**). Of these, only 53,270 (3.04%) had a WM referral recorded, mirroring the rate in the full descriptive sample. A total of 1,228,024 had complete data for all covariables and were included in the adjusted analysis.

**Table 4:** RRs for NHS WM referral within adults eligible for WM referral in England with at least 2 years of follow-up data in CPRD GOLD (2007–2020)^a^ (MODEL A).

	Eligible for a WM referral (N =)	Had a WM referral recorded (N =) (% of those eligible)	Crude RR (95% CI)	*P* value	Adjusted RR (95% CI)^b^	*P* value
**Total**	1,751,385	53,270 (3.04)	1,751,385^c^		1,228,024^d^	
**Sex** ^**e**^						
Male	795,602	17,896 (2.25)	0.61 (0.60,0.62)	<0.001	0.69 (0.68,0.71)	<0.001
*Female*	955,783	35,374 (3.70)	1.0		1.0	
**Age group at diagnosis with overweight or obesity**						
18–24	107,515	2,464 (2.29)	0.54 (0.52,0.57)	<0.001	0.52 (0.49,0.55)	<0.001
25–34	206,868	5,342 (2.58)	0.61 (0.59,0.63)	<0.001	0.59 (0.57,0.62)	<0.001
35–44	295,335	12,174 (4.12)	0.98 (0.96,1.00)	0.072	0.95 (0.93,0.98)	0.001
*45–54*	351,288	14,796 (4.21)	1.0		1.0	
55–64	338,340	11,998 (3.55)	0.84 (0.82,0.86)	<0.001	0.91 (0.89,0.94)	<0.001
65–74	269,041	5,695 (2.12)	0.50 (0.49,0.52)	<0.001	0.60 (0.58,0.62)	<0.001
75+	182,998	801 (0.44)	0.10 (0.10,0.11)	<0.001	0.15 (0.13,0.16)	<0.001
**Strategic Health Authority of GP practice**						
North East	39,114	1,397 (3.57)	1.24 (1.17,1.31)	<0.001	1.60 (1.50,1.70)	<0.001
*North West*	271,163	7,826 (2.89)	1.0		1.0	
Yorkshire and the Humber	63,586	751 (1.18)	0.41 (0.38,0.44)	<0.001	0.61 (0.56,0.65)	<0.001
East Midlands	62,955	1,825 (2.90)	1.00 (0.96,1.06)	0.863	1.61 (1.52,1.70)	<0.001
West Midlands	237,361	12,828 (5.40)	1.87 (1.82,1.92)	<0.001	2.17 (2.10,2.24)	<0.001
East of England	169,466	1,762 (1.04)	0.36 (0.34,0.38)	<0.001	0.43 (0.41,0.46)	<0.001
South West	196,943	4,613 (2.34)	0.81 (0.78,0.84)	<0.001	0.98 (0.94,1.02)	0.235
South Central	237,355	6,566 (2.77)	0.96 (0.93,0.99)	0.010	1.21 (1.17,1.26)	<0.001
London	214,783	5,836 (2.72)	0.94 (0.91,0.97)	<0.001	1.11 (1.06,1.15)	<0.001
South East Coast	258,659	9,866 (3.81)	1.32 (1.28,1.36)	<0.001	1.64 (1.59,1.70)	<0.001
**Rural–urban classification of GP practice**						
*Urban*	1,196,702	35,217 (2.94)	1.0		1.0	
Rural	180,091	4,376 (2.43)	0.83 (0.80,0.85)	<0.001	0.88 (0.85,0.90)	<0.001
Data missing/not recorded	*374*,*592*	*13*,*677 (3*.*65)*	-		-	
**Year of diagnosis with overweight/obesity**						
*2007*	531,157	16,637 (3.13)	1.0		1.0	
2008	271,624	7,842 (2.89)	0.92 (0.90,0.95)	<0.001	0.95 (0.93,0.98)	0.003
2009	192,610	5,447 (2.83)	0.90 (0.88,0.93)	<0.001	0.93 (0.89,0.96)	<0.001
2010	154,843	3,807 (2.46)	0.78 (0.76,0.81)	<0.001	0.85 (0.81,0.88)	<0.001
2011	131,933	3,352 (2.54)	0.81 (0.78,0.84)	<0.001	0.89 (0.85,0.93)	<0.001
2012	123,252	3,380 (2.74)	0.88 (0.84,0.91)	<0.001	0.97 (0.93,1.01)	0.176
2013	108,539	3,696 (3.41)	1.09 (1.05,1.13)	<0.001	1.27 (1.22,1.32)	<0.001
2014	83,549	3,088 (3.70)	1.18 (1.14,1.23)	<0.001	1.31 (1.25,1.37)	<0.001
2015	64,674	2,455 (3.80)	1.21 (1.16,1.26)	<0.001	1.25 (1.19,1.32)	<0.001
2016	43,212	1,664 (3.85)	1.23 (1.17,1.29)	<0.001	1.36 (1.28,1.44)	<0.001
2017	33,218	1,388 (4.18)	1.33 (1.26,1.41)	<0.001	1.25 (1.17,1.34)	<0.001
2018	12,774	514 (4.02)	1.28 (1.18,1.40)	<0.001	1.34 (1.21,1.49)	<0.001
**BMI category (kg/m** ^**2**^ **) at diagnosis with overweight/obesity**						
23.0–24.9 (B&A only)	29,158	178 (0.61)	0.45 (0.39,0.52)	<0.001	0.36 (0.30,0.43)	<0.001
*25*.*0–29*.*9*	1,006,478	13,529 (1.34)	1.0		1.0	
30.0–34.9	444,510	17,240 (3.88)	2.89 (2.82,2.95)	<0.001	2.83 (2.75,2.90)	<0.001
35.0–40.0	162,595	10,025 (6.17)	4.59 (4.47,4.70)	<0.001	4.20 (4.07,4.33)	<0.001
40.0 +	83,901	8,436 (10.05)	7.48 (7.29,7.68)	<0.001	6.19 (5.99,6.40)	<0.001
Medical codes	24,743	3,862 (15.61)	11.61 (11.23,12.01)	<0.001	8.88 (8.53,9.25)	<0.001
**Ethnic group**						
*White*	1,322,847	42,347 (3.20)	1.0		1.0	
Asian	70,014	1,723 (2.46)	0.77 (0.73,0.81)	<0.001	0.99 (0.93,1.05)	0.726
Black	42,712	1,520 (3.56)	1.11 (1.06,1.17)	<0.001	1.19 (1.12,1.26)	<0.001
Mixed	12,009	355 (2.96)	0.92 (0.83,1.02)	0.129	1.01 (0.89,1.14)	0.926
Other	19,647	576 (2.93)	0.92 (0.84,0.99)	0.033	0.92 (0.84,1.02)	0.101
Unknown	284,156	6,749 (2.38)	0.74 (0.72,0.76)	<0.001	0.71 (0.68,0.74)	<0.001
**IMD**						
*1 (least deprived)*	290,170	7,017 (2.42)	1.0		1.0	
2	288,664	8,374 (2.90)	1.20 (1.16,1.24)	<0.001	1.13 (1.09,1.16)	<0.001
3	279,202	7,219 (2.59)	1.07 (1.04,1.10)	<0.001	0.99 (0.96,1.03)	0.665
4	259,566	7,875 (3.03)	1.25 (1.22,1.30)	<0.001	1.10 (1.06,1.13)	<0.001
5 (most deprived)	235,220	8,696 (3.70)	1.53 (1.48,1.58)	<0.001	1.16 (1.13,1.20)	<0.001
Data missing/not recorded	398,563	14,089 (3.53)	-		-	
**Smoking status**						
*Nonsmoker*	802,920	26,526 (3.30)	1.0		1.0	
Current smoker	330,257	11,084 (3.36)	1.02 (0.99,1.04)	0.156	0.93 (0.91,0.95)	<0.001
Ex-smoker	453,803	13,763 (3.03)	0.92 (0.90,0.94)	<0.001	1.00 (0.97,1.02)	0.798
Data missing/not recorded	164,405	1,897 (1.15)			-	
**Presence of comorbidities** ^**f**^						
Type 2 diabetes	262,713	8,951 (3.41)	1.14 (1.12,1.17)	<0.001	0.92 (0.90,0.95)	<0.001
Hypertension	483,016	12,870 (2.66)	0.84 (0.82,0.85)	<0.001	0.78 (0.76,0.80)	<0.001
Coronary heart disease	219,434	4,426 (2.02)	0.63 (0.61,0.65)	<0.001	0.83 (0.80,0.86)	<0.001
Obstructive sleep apnoea	27,969	2,305 (8.24)	2.79 (2.68,2.90)	<0.001	1.63 (1.55,1.70)	<0.001
Asthma	271,421	9,759 (3.60)	1.22 (1.20,1.25)	<0.001	1.01 (0.98,1.03)	0.623
Chronic musculoskeletal condition	381,461	13,248 (3.47)	1.19 (1.17,1.21)	<0.001	1.21 (1.18,1.24)	<0.001
Gastro-oesophageal reflux disease	485,268	17,286 (3.56)	1.25 (1.23,1.28)	<0.001	1.14 (1.11,1.16)	<0.001
Liver disease	37,430	1,325 (3.54)	1.17 (1.11,1.23)	<0.001	1.02 (0.97,1.08)	0.435
Polycystic ovarian syndrome	25,879	1,598 (6.17)	2.06 (1.96,2.16)	<0.001	1.29 (1.22,1.37)	<0.001
Fertility problems	41,151	1,763 (4.28)	1.42 (1.36,1.49)	<0.001	1.11 (1.06,1.18)	<0.001
Depression	456,930	19,164 (4.19)	1.59 (1.56,1.62)	<0.001	1.17 (1.14,1.19)	<0.001
Anxiety	337,680	13,148 (3.89)	1.37 (1.35,1.40)	<0.001	1.07 (1.05,1.10)	<0.001
Idiopathic intracranial hypertension	1,635	146 (8.93)	2.94 (2.52,3.43)	<0.001	1.31 (1.11,1.56)	0.002

Text in *italics* indicates the reference group for the Poisson regression model.

B&A, Black and Asian ethnic groups; BMI, body mass index; CI, confidence interval; CPRD, Clinical Practice Research Datalink; GP, general practitioner; IMD, Index of Multiple Deprivation; NHS, National Health Service; RR, rate ratio; T2DM, type 2 diabetes mellitus; WM, weight management.

^a^Patients without “research quality” data excluded (*N* = 18,935).

^b^Variables included in adjusted model include sex, age group at diagnosis with overweight/obesity, Strategic Health Authority of GP practice, rural–urban classification of GP practice, year of diagnosis with overweight/obesity, BMI category at diagnosis with overweight/obesity, ethnic group, IMD, smoking status, presence of comorbidities (each of 13 comorbidities included individually in the model).

^c^Number of individuals included in the crude analysis.

^d^Number of individuals with complete data for all variables included in the adjusted analysis.

^e^Indeterminate and unknown sex excluded due to small numbers (*n* = 18).

^f^Compared with not having comorbidity.

**Model A**. The likelihood of WM referral was greater in women (RR 0.69, 95% CI [0.68,0.71] for men compared with women), middle age groups (e.g., RR 0.52, 95% CI [0.49,0.55] for 18 to 24 compared with 45 to 54 years), people of Black ethnicity (RR 1.19, 95% CI [1.12,1.26] compared with White), and diagnosis with overweight/obesity in later study years (e.g., RR 1.36, 95% CI [1.28,1.44] for 2016 compared with 2007). People in IMD categories 2, 4, and 5 (most deprived) were more likely to have a WM referral than those in IMD category 1 (least deprived) (e.g., RR 1.16, 95% CI [1.13,1.20] for deprivation level 5 compared with level 1). Current smokers were less likely to have a referral (RR 0.93, 95% CI [0.91,0.95] compared with nonsmokers).

Increasing BMI category was associated with an increased likelihood of referral with people with a BMI ≥40 kg/m^2^, more than 6 times as likely to have a referral than BMI 25.0 to 29.9 kg/m^2^ (RR 6.19, 95% CI [5.99,6.40]). Having obstructive sleep apnoea (OSA), a chronic musculoskeletal condition, gastro-oesophageal reflux disease (GORD), polycystic ovarian syndrome (PCOS), fertility problems, depression, anxiety, or idiopathic intracranial hypertension (IIH) were associated with increased likelihood of referral (e.g., RR 1.63, 95% CI [1.55,1.70] for OSA), whereas people with T2DM, hypertension (HTN), or coronary heart disease (CHD) were less likely to have a referral (e.g., RR 0.78, 95% CI [0.76,0.80] for HTN) (**Table 4**). Differences were seen across regions with GP practices from the West Midlands more than twice as likely to refer (RR 2.17, 95% CI [2.10,2.24]), and practices from the East of England less than half as likely to refer (RR 0.43, 95% CI [0.41,0.46]) compared with the North West. Rural GP practices were less likely to refer than urban practices (RR 0.88, 95% CI [0.85,0.90]).

### Bariatric surgery

A total of 297,332 patients with severe and complex obesity were eligible for HES linkage, had at least 5 years of follow-up and did not have bariatric surgery prior to their severe and complex obesity index date (68% of all 436,501 patients with severe and complex obesity in the study), and were eligible to be included in the regression analysis (**Fig 1 and Table 5**). Of these, only 3,604 (1.21%) underwent bariatric surgery, mirroring the rate in the descriptive sample. A total of 280,316 had complete data for all covariables and were included in the adjusted analysis.

**Table 5:** RRs for provision of bariatric surgery within adults eligible for bariatric surgery and HES linkage in England with at least 5 years of follow-up data in CPRD GOLD (2007–2020)^a^.

	Eligible for bariatric surgery (*N =*)	Underwent bariatric surgery (N =) (% of those eligible)	Crude RR (95% CI)	*P* value	Adjusted RR (95% CI)^b^	*P* value
**Total**	297,332	3,604 (1.21)	297,332^c^		280,316^d^	
**Sex**						
Male	125,147	852 (0.68)	0.43 (0.39,0.46)	<0.001	0.77 (0.71,0.84)	<0.001
*Female*	172,185	2,752 (1.60)	1.0		1.0	
**Age group at diagnosis with severe and complex obesity**						
18–24	11,173	237 (2.12)	1.21 (1.05,1.39)	0.008	1.30 (1.11,1.52)	0.001
25–34	26,016	696 (2.68)	1.52 (1.39,1.67)	<0.001	1.47 (1.32,1.65)	<0.001
35–44	43,386	1,174 (2.71)	1.54 (1.42,1.67)	<0.001	1.37 (1.25,1.50)	<0.001
*45–54*	60,640	1,065 (1.76)	1.0		1.0	
55–64	66,549	384 (0.58)	0.33 (0.29,0.37)	<0.001	0.37 (0.33,0.42)	<0.001
65–74	54,893	47 (0.09)	0.05 (0.04,0.07)	<0.001	0.07 (0.06,0.10)	<0.001
75+	34,675	1 (0.00)	0.00 (0.00,0.01)	<0.001	0.00 (0.00,0.03)	<0.001
**Strategic Health Authority of GP practice**						
North East	7,951	188 (2.36)	3.46 (2.91,4.13)	<0.001	3.05 (2.56,3.64)	<0.001
*North West*	52,436	358 (0.68)	1.0		1.0	
Yorkshire and the Humber	12,627	167 (1.32)	1.94 (1.61,2.32)	<0.001	1.95 (1.63,2.35)	<0.001
East Midlands	9,836	102 (1.04)	1.52 (1.22,1.89)	<0.001	1.38 (1.10,1.74)	0.006
West Midlands	36,765	391 (1.06)	1.56 (1.35,1.80)	<0.001	1.48 (1.28,1.71)	<0.001
East of England	29,996	257 (0.86)	1.25 (1.07,1.47)	0.005	1.26 (1.07,1.48)	0.006
South West	40,610	483 (1.19)	1.74 (1.52,2.00)	<0.001	1.73 (1.51,1.99)	<0.001
South Central	36,366	423 (1.16)	1.70 (1.48,1.96)	<0.001	1.68 (1.45,1.94)	<0.001
London	31,359	673 (2.15)	3.14 (2.77,3.57)	<0.001	3.29 (2.88,3.76)	<0.001
South East Coast	39,386	562 (1.43)	2.09 (1.83,2.38)	<0.001	2.17 (1.90,2.49)	<0.001
**Rural–urban classification of GP practice**						
*Urban*	258,135	3,197 (1.24)	1.0		1.0	
Rural	39,197	407 (1.04)	0.84 (0.76, 0.93)	0.001	1.02 (0.92, 1.14)	0.718
Data missing/not recorded		-	-		-	
**Year of diagnosis with severe and complex obesity**						
*2007*	99,441	1,658 (1.67)	1.0		1.0	
2008	42,133	666 (1.58)	0.95 (0.87,1.04)	0.241	0.87 (0.79,0.95)	0.002
2009	32,030	387 (1.21)	0.72 (0.65,0.81)	<0.001	0.70 (0.62,0.78)	<0.001
2010	28,358	255 (0.90)	0.54 (0.47,0.61)	<0.001	0.59 (0.52,0.68)	<0.001
2011	25,679	226 (0.88)	0.53 (0.46,0.61)	<0.001	0.57 (0.50,0.66)	<0.001
2012	23,958	165 (0.69)	0.41 (0.35,0.48)	<0.001	0.51 (0.43,0.60)	<0.001
2013	21,740	123 (0.57)	0.34 (0.28,0.41)	<0.001	0.40 (0.33,0.49)	<0.001
2014	17,304	101 (0.58)	0.35 (0.29,0.43)	<0.001	0.40 (0.32,0.49)	<0.001
2015	6,689	23 (0.34)	0.21 (0.14,0.31)	<0.001	0.20 (0.12,0.32)	<0.001
**BMI category (kg/m** ^**2**^ **) at diagnosis with severe and complex obesity**						
27.5–29.9 in B&A groups with T2DM diagnosed no more than 10 years prior to first eligible BMI measurement	36,429	7 (0.02)	0.04 (0.02,0.08)	<0.001	0.09 (0.04,0.18)	<0.001
30.0–34.9 with T2DM diagnosed no more than 10 years prior to first eligible BMI measurement	52,669	44 (0.08)	0.16 (0.12,0.21)	<0.001	0.28 (0.21,0.39)	<0.001
*35*.*0–40*.*0 with weight related comorbidity*	137,378	730 (0.53)	1.0		1.0	
40.0 +	70,713	2,817 (3.98)	7.50 (6.91,8.13)	<0.001	5.52 (5.07,6.02)	<0.001
Diagnosis of severe and complex obesity as per medical codes	143	6 (4.20)	7.90 (3.60,17.34)	<0.001	12.26 (5.48,27.47)	<0.001
***Ethnic group***						
*White*	258,969	3,268 (1.26)	1.0		1.0	
Asian	9,113	89 (0.98)	0.77 (0.63,0.95)	0.017	0.94 (0.76,1.18)	0.606
Black	7,263	117 (1.61)	1.28 (1.06,1.53)	0.009	0.90 (0.74,1.09)	0.290
Mixed	1,430	42 (2.94)	2.33 (1.72,3.14)	<0.001	1.46 (1.06,2.01)	0.020
Other	2,713	61 (2.25)	1.78 (1.39,2.29)	<0.001	1.24 (0.95,1.62)	0.120
Unknown	17,844	27 (0.15)	0.12 (0.08,0.18)	<0.001	0.14 (0.09,0.21)	<0.001
**IMD**						
*1 (least deprived)*	49,899	431 (0.86)	1.0		1.0	
2	58,437	626 (1.07)	1.24 (1.10,1.40)	0.001	1.09 (0.96,1.23)	0.200
3	61,865	741 (1.20)	1.39 (1.23,1.56)	<0.001	1.11 (0.98,1.25)	0.088
4	62,890	870 (1.38)	1.60 (1.43,1.80)	<0.001	1.03 (0.91,1.15)	0.666
5 (most deprived)	64,054	935 (1.46)	1.69 (1.51,1.89)	<0.001	1.02 (0.90,1.15)	0.732
Data missing/not recorded	187	1 (0.53)	-		-	
**Smoking status**						
*Nonsmoker*	131,749	1,580 (1.20)	1.0		1.0	
Current smoker	59,622	767 (1.29)	1.07 (0.98,1.17)	0.109	0.87 (0.80,0.95)	0.001
Ex-smoker	89,118	961 (1.08)	0.90 (0.83,0.97)	0.009	1.22 (1.13,1.32)	<0.001
Data missing/not recorded	16,843	296 (1.76)	-		-	
**Presence of comorbidities** ^**e**^						
Type 2 diabetes	146,114	1,214 (0.83)	0.53 (0.49,0.56)	<0.001	1.19 (1.10,1.29)	<0.001
Hypertension	126,748	1,152 (0.91)	0.63 (0.59,0.68)	<0.001	0.98 (0.91,1.06)	0.683
Coronary heart Disease	62,171	362 (0.58)	0.42 (0.38,0.47)	<0.001	0.81 (0.72,0.91)	0.001
Obstructive sleep apnoea	11,899	603 (5.07)	4.82 (4.42,5.25)	<0.001	2.59 (2.36,2.84)	<0.001
Asthma	65,730	1,169 (1.78)	1.69 (1.58,1.81)	<0.001	1.13 (1.05,1.22)	0.001
Chronic musculoskeletal condition	99,615	1,253 (1.26)	1.06 (0.99,1.13)	0.106	1.71 (1.58,1.85)	<0.001
Gastro-oesophageal reflux disease	103,322	1,575 (1.52)	1.46 (1.37,1.56)	<0.001	1.35 (1.26,1.45)	<0.001
Liver disease	13,763	400 (2.91)	2.57 (2.32,2.85)	<0.001	2.21 (1.99,2.46)	<0.001
Polycystic ovarian syndrome	7,730	394 (5.10)	4.60 (4.15,5.09)	<0.001	1.75 (1.57,1.96)	<0.001
Fertility problems	7,343	227 (3.09)	2.65 (2.33,3.03)	<0.001	1.14 (0.99,1.31)	0.074
Depression	105,265	2,173 (2.06)	2.77 (2.59,2.96)	<0.001	1.71 (1.58,1.84)	<0.001
Anxiety	71,305	1,221 (1.71)	1.62 (1.52,1.74)	<0.001	0.96 (0.89,1.03)	0.231
Idiopathic intracranial hypertension	794	45 (5.67)	4.72 (3.55,6.28)	<0.001	1.45 (1.07,1.95)	0.016

Text in *italics* indicates the reference group for the Poisson regression model.

B&A, Black and Asian ethnic groups; BMI, body mass index; CI, confidence interval; CPRD, Clinical Practice Research Datalink; GP, general practitioner; HES, Hospital Episode Statistics; IMD, Index of Multiple Deprivation; RR, rate ratio; T2DM, type 2 diabetes mellitus.

^a^A total of 21 individuals with bariatric surgery before their severe and complex obesity index date excluded.

^b^Variables included in adjusted model include sex, age group at diagnosis with severe and complex obesity, Strategic Health Authority of GP practice, rural–urban classification of GP practice, year of diagnosis with severe and complex obesity, BMI category at diagnosis with severe and complex obesity, ethnic group, IMD, smoking status, presence of comorbidities (each of 13 comorbidities included individually in the model).

^c^Number of individuals included in the crude analysis.

^d^Number of individuals with complete data for all variables included in the adjusted analysis.

^e^Compared with not having comorbidity.

**Model A**. The likelihood of undergoing bariatric surgery was greater in women (RR 0.77, 95% CI [0.71,0.84] for men compared with women), younger age groups (e.g., RR 1.47, 95% CI [1.32,1.65] for 25 to 34 compared with 45 to 54 years), and longer duration since diagnosis of severe and complex obesity (i.e., diagnosis in earlier study years) (e.g., RR 0.20, 95% CI [0.12,0.32] for 2015 compared with 2007). Compared with nonsmokers, ex-smokers were more likely to undergo bariatric surgery (RR 1.22, 95% CI [1.13,1.32]), and current smokers less likely (RR 0.87, 95% CI [0.80,0.95]). There was some evidence that those of Mixed ethnicity were more likely to undergo bariatric surgery than those of White ethnicity (RR 1.46, 95% CI [1.06,2.01]), but not for Asian, Black, or Other ethnic groups (although those where ethnicity was “Unknown” were less likely to undergo surgery). No differences were found for level of deprivation.

Increasing BMI category was associated with an increased likelihood of surgery, with people with a BMI ≥40 kg/m^2^ more than 5 times as likely to undergo surgery than BMI 35.0 to 40.0 kg/m^2^ with a comorbidity (RR 5.52, 95% CI [5.07,6.02]). Having T2DM, OSA, asthma, a chronic musculoskeletal condition, GORD, liver disease, PCOS, depression and IIH were associated with increased likelihood of bariatric surgery (e.g., RR 2.59, 95% CI [2.36,2.84] for OSA), whereas people with CHD were less likely to undergo surgery (RR 0.81, 95% CI [0.72,0.91]). Differences were seen across regions with individuals from GP practices in London more than 3 times as likely to have bariatric surgery (RR 3.29, 95% CI [2.88,3.76]) than those from the North West. There was, however, no difference been rural or urban GP practices.

**Model B**. Having an increasing number of comorbidities slightly increased the likelihood of WM referral and greatly increased the likelihood of bariatric surgery (**S2 and S3 Tables**). For example, people with 6 or more comorbidities were nearly 9 times as likely to undergo bariatric surgery than those with no comorbidities (RR 8.79, 95% CI [7.16,10.79]). Results were the same for other covariables compared with model A.

### Sensitivity analysis

Characteristics of patients included in the adjusted regression analyses (with complete data for all variables) were very similar to patients in the corresponding full samples (**S4 and S5 Tables**). When removing people with “Unknown” ethnicity from both the WM referral (*n* = 284,156) and the bariatric surgery (*n* = 17,844) adjusted analyses, the results were unchanged (for both models A and B) (**S5 and S6 Appendices**).

## Discussion

This study found that just 3% of those with a recorded diagnosis of overweight/obesity in CPRD in England between 2007 and 2020 had a referral for WM recorded. Given that adults with recorded overweight and obesity in CPRD (31%) represent about half that estimated by the HSE (64%), this may translate to only 1.5% of the truly eligible population receiving a WM referral [2]. A similarly small proportion (1%) of those eligible for NHS bariatric surgery received this. WM referrals and bariatric surgery were low across all groups. Higher BMI and region of GP practice had the strongest associations for both WM referral and bariatric surgery. Longer duration since diagnosis with severe and complex obesity and increasing comorbidities were strongly associated with undergoing surgery. These observed practices urgently require further attention and investigation.

The differences in overweight and obesity estimated by the HSE and this study may reflect reporting practice. In our data set, there is evidence that recording increased from 2007 to 2015. This may be due to the introduction in 2006 to 2007 of the Quality Outcomes Framework (QOF) indicator to keep a register of people with BMI ≥30 kg/m^2^, and the publication of the first NICE guidance for obesity [38–40]. Underrecording of obesity and BMI in primary care has been recognised as an issue, with studies reporting increasing age, female sex, comorbidities, and higher BMI to be positively associated with BMI recording [38,41–43]. Underrecording may be due to multiple factors, such as consultation time pressures, perceptions that primary care is not the place to deal with obesity, and the need for more training in obesity; these may, in turn, influence the offer of WM referrals [44,45]. To our knowledge, this is the first study to estimate the proportion of the English primary care population potentially eligible for bariatric surgery according to the 2014 NICE obesity guidance; this agrees with estimates of bariatric surgery eligibility (7.8% of the population) using HSE data [27].

Booth and colleagues found that between 2005 and 2012, approximately 17% of people with BMI ≥40 kg/m^2^ in CPRD received a referral (versus 10% in our study); however, this included referrals to dietitians and exercise therapy (which may or may not have been specifically for WM) in addition to WM programmes [21]. Lemp and colleagues found that between 2010 and 2019, 32% of people newly diagnosed with obesity in CPRD received a lifestyle intervention [46]. However, lifestyle interventions did not only pertain to WM, and diagnosis of obesity was defined by clinical codes only. It has been reported that the assignment of an obesity clinical code may indicate a marker of clinician concern [21]. This may help explain why we found the highest percentage of WM referrals in people who had only an obesity medical code that could not be mapped to a BMI category (16% versus 10% in BMI ≥40 kg/m^2^). Within a 2013 to 2014 cluster RCT, 30 general practices in England were randomised to receive or not receive training in implementing WM guidelines, with the outcome the proportion of people with overweight/obesity who received a WM intervention (defined as advice or referral) [47]. Just 3.7% to 5.1% received an external WM referral (private or publicly funded), which is closer to the figures found in our study. No differences were seen between groups with the authors concluding the intervention did not improve implementation of WM guidelines. Welbourn and colleagues previously suggested that NHS bariatric surgery meets less than 1% of the UK need [25]. Our study supports this, estimating that 1.09% of those eligible for NHS bariatric surgery in England between 2007 and 2020 actually underwent this. Although we applied the 2014 NICE criteria for bariatric surgery (which widened the access criteria from the original guidance) to the whole study period (2007 to 2020), the National Bariatric Surgery Registry (NBSR) reported that NHS bariatric surgery decreased between 2014 and 2019. Currie and colleagues recently reported that the 2014 NICE criteria has had little impact on the rate of bariatric surgery [48,49]. Our study did, however, find an increased likelihood of WM referrals from 2013 onwards. It is possible that the updated NICE obesity guidance helped to improve awareness and/or availability of nonsurgical WM services.

We found BMI category to have the strongest association with receiving a WM referral or bariatric surgery with those in the highest BMI category most likely, similar to Booth and colleagues [21]. People with BMI 23.0 to 24.9 from Black and Asian groups were the least likely to have a WM referral; however, in the overall sample, people of Black ethnicity were slightly more likely to receive a WM referral than those of White ethnicity. The former finding may reflect a lack of awareness of lower ethnicity specific BMI cutoffs to access WM interventions. Previous CPRD studies did not include people within these lower ethnicity-specific BMI cutoffs within their cohorts. There was an increased focus on lower BMI thresholds in Black and Asian ethnic groups in the 2014 NICE obesity guidance, and 2022 updates to the document have expanded the ethnicities included in the lower BMI cutoffs [11]. We found a negative association between T2DM and CHD, and WM referral, whereas both these comorbidities were positively associated with WM interventions in Booth and colleagues’ study. As our study focused on WM referrals specifically, our finding may be explained by people with these conditions instead receiving other comorbidity-specific programmes, which may include a WM component (such as cardiac rehabilitation programmes); however, further research is needed to confirm this. To our knowledge, this is one of the first studies to comprehensively investigate a range of characteristics associated with receiving NHS bariatric surgery versus not receiving surgery. Bolckmans and colleagues reported that NHS bariatric surgery patients were significantly older and more comorbid than self-paying patients [50]. We also found that an increasing number of comorbidities was associated with undergoing surgery. The reasons for this are unclear but may include low confidence of GPs in discussing obesity and bariatric surgery with one study reporting it took an average of 9 years for a clinical conversation about weight to occur after concerns about weight started, delayed help-seeking of people living with obesity, and the lengthy pathway to undergo bariatric surgery in the NHS [51–58].

Despite national guidance with respect to the availability of WM services and bariatric surgery, our study revealed striking regional inequalities. These may reflect differences in commissioning priorities and “patchy” provision of WM services and NHS bariatric centres across the country [59]. For example, people within the East of England and the North West are the least likely to undergo bariatric surgery where there are the lowest number of NHS bariatric centres. Low and variable provision of WM services has also been reported to be an issue in Scotland and Wales, and bariatric surgery has been reported to be disproportionately lower in the devolved nations, with no bariatric surgery commissioned at all in Northern Ireland [49,60–65]. Individuals undergoing bariatric surgery in Scotland were on average heavier than those in this study, with all having a BMI ≥40 [65]. Bariatric surgery criteria in Scotland is more restrictive than NICE guidance, recommending prioritising bariatric surgery for people with recent onset T2DM, who have a BMI ≥35 [66].

We have been unable to identify comparable international data on access to WM interventions due to differences in health system models, although some data on bariatric surgery is available [67]. Several European countries with a lower prevalence of obesity than the UK undertake more bariatric surgery [68,69]. The reasons for this are unclear but could be related to differences in social and political attitudes to obesity, and more straightforward pathways to bariatric surgery in other countries [67]. A 2015 systematic review and meta-analysis, which pooled data from bariatric surgery retrospective cohort studies from the United States of America, Canada, UK, and Australia, found that overall, less than 1% of eligible patients received surgery [70]. The review found that women, adults under age 50, of White ethnicity, and from urban areas were more likely to undergo surgery, whereas our study did not find evidence of the latter two.

A key strength of this study was the size of cohort drawn from a large national primary care dataset considered to be representative of the UK population, linked with NHS bariatric surgery hospital data. A weakness of all studies using routinely collected primary care data is reliance on clinical data being recorded in patients’ primary care records. This may have introduced selection bias in this study as those living with overweight or obesity who have a diagnosis documented in their GP records may be different in some way to those who do not. Herrett and colleagues, however, previously reported that BMI distribution in CPRD is comparable to the HSE [22]. Another limitation is that the Vision practice software which CPRD GOLD draws on has been declining in use in recent years, and thus it is possible there could be an issue of reducing representativeness of practices in CPRD GOLD over time. This study only captures WM referrals made from primary care and was not able to capture patient self-referrals, or referrals from other non-primary care–based health and social care professionals, which is possible for some community-based NHS WM services. We used a focused definition of WM referral to ensure that we correctly identified referrals for WM in line with the NICE guidance. It is possible we missed some programmes that include a WM component but may not be coded as such in primary care. For example, since 2016, there has been a focus in England on the Diabetes Prevention Programme (for people with prediabetes), and in 2022, the NHS Type 2 Diabetes Path to Remission Programme pilot was launched (for people with T2DM) [71,72]. For pseudonymisation purposes, CPRD does not include Integrated Care Board or Clinical Commissioning Group (CCG)-level geography, thus we were unable to compare different smaller geographies (e.g., CCGs), which may have their own WM policies that are more restrictive than NICE guidance.

Since this study was undertaken, new national initiatives have been launched. The introduction of the 12-week NHS Digital WM programme in 2021 for people with T2DM and/or HTN is a first step towards improved access to WM across England [73]. However, not everyone is able to access or engage with digital services, and there is a concern that the reliance on remote WM services since COVID-19 could widen inequalities without adequate in-person provision alongside this [74,75]. In 2021 to 2022, the Enhanced Service Specification (ESS) for WM was introduced incentivising GP practices to improve recording of overweight or obesity and refer to WM programmes [76–78]. Theis and White identified that one of the key failings of obesity strategies and policies in England between 1992 and 2020 was a lack of information on how to implement policies, including limited monitoring and evaluation [79]. The recently launched National Obesity Audit at present has published a dashboard of NHS bariatric surgery data from HES and, in time, will also include data from primary care and community and specialist (tier 2 and 3) WM programmes [19]. A national database of specialist (tier 3) WM services is also in development, and, together with the National Obesity Audit, there will hopefully be better data to evaluate the availability and outcomes of services nationally in the years to come, including the impact on inequalities [80]. Together with strong population-wide obesity prevention strategies, how to improve equity in WM provision across the country should be an important focus of national policies related to obesity going forward [79]. Qualitative research with commissioners, health professionals, and people living with obesity is currently underway within our research team to provide a more in-depth understanding of the barriers to accessing WM services, and potential solutions.

Our study suggests that access to WM programmes in England is very low and has not improved over the last 10 years. Our study agrees with previous research describing the demographic and clinical characteristics of adults who are referred for WM and undergo bariatric surgery. We have importantly highlighted the regional inequalities in access to WM interventions across England. We recommend that future research considers how to improve regional equity in access to WM across the country, including the evaluation of recent national WM initiatives.

## Supporting information

S1 Checklist STROBE (Strengthening the Reporting of Observational Studies in Epidemiology) and-RECORD (Reporting of studies Conducted using Observational Routinely-collected health Data (RECORD) statements. (DOCX)

S1 Appendix Approved ISAC protocol including statistical analysis plan. ISAC, Independent Scientific Advisory Committee.
(PDF)

S2 Appendix Exposure and outcomes codelists. (XLSX)

S3 Appendix Covariables codelists. (XLSX)

S4 Appendix NHS bariatric surgery centres in England, by Strategic Health Authority region. NHS, National Health Service.
(DOCX)

S5 Appendix Adjusted RRs for WM referral within adults eligible for WM referral in England with at least 2 years of follow-up data in CPRD GOLD (2007–2020)—People with unknown ethnicity dropped (*n =* 284,156). RR, rate ratio; WM, weight management.
(TXT)

S6 Appendix Adjusted RRs for provision of bariatric surgery within adults eligible for bariatric surgery and HES linkage in England with at least 5 years of follow-up data in CPRD GOLD (2007–2020)—People with unknown ethnicity dropped (*n* = 17,844). CPRD, Clinical Practice Research Datalink; HES, Hospital Episode Statistics; RR, rate ratio.
(TXT)

S1 Table Characteristics of adults with recorded overweight and obesity in England in CPRD GOLD, by BMI group (all years 2007–2020). B&A, Black and Asian ethnic groups; BMI, body mass index; CPRD, Clinical Practice Research Datalink. ^a^Percentage of the total variable. ^b^Medical codes indicating diagnosis with overweight or obesity where BMI category not specified. ^c^Percentage of “All” (*n* = 1,811,587).
(DOCX)

S2 Table RRs for NHS WM referral within adults eligible for WM referral in England with at least 2 years of follow-up data in CPRD GOLD (2007–2020)^a^ (MODEL B). Text in *italics* indicates the reference group for the Poisson regression model. B&A, Black and Asian ethnic groups; BMI, body mass index; CI, confidence interval; CPRD, Clinical Practice Research Datalink; IMD, Index of Multiple Deprivation; NHS, National Health Service; RR, rate ratio; WM, weight management. ^a^Patients without “research quality” data excluded (*N =* 18,935). ^b^Variables included in adjusted model include sex, age group at diagnosis with overweight/obesity, strategic Health Authority of GP practice, rural–urban classification of GP practice, year of diagnosis with overweight/obesity, BMI category at diagnosis with overweight/obesity, ethnic group, IMD, smoking status, total number of comorbidities. ^c^Number of individuals included in the crude analysis. ^d^Number of individuals with complete data for all variables included in the adjusted analysis. ^e^Indeterminate and unknown sex excluded due to small numbers (*n =* 18).
(DOCX)

S3 Table RRs for provision of bariatric surgery within adults eligible for bariatric surgery in England with at least 5 years of follow-up data in CPRD GOLD (2007–2020).^a^ (MODEL B). Text in *italics* indicates the reference group for the Poisson regression model. B&A, Black and Asian ethnic groups; BMI, body mass index; CI, confidence interval; CPRD, Clinical Practice Research Datalink; GP, general practitioner; IMD, Index of Multiple Deprivation; RR, rate ratio. ^a^A total of 21 individuals with bariatric surgery before their severe and complex obesity index date excluded. ^b^Variables included in adjusted model include sex, age group at diagnosis with severe and complex obesity, strategic health authority of GP practice, rural–urban classification of GP practice, year of diagnosis with severe and complex obesity, BMI category at diagnosis with severe and complex obesity, ethnic group, IMD, smoking status, total number of comorbidities. ^c^Number of individuals included in the crude analysis. ^d^Number of individuals with complete data for all variables included in the adjusted analysis.
(DOCX)

S4 Table Characteristics of patients included in adjusted Poisson model versus those in full sample (WM referral as outcome). B&A, Black and Asian ethnic groups; BMI, body mass index; WM, weight management. ^a^Included in adjusted model. ^b^Medical codes indicating diagnosis with overweight or obesity where BMI category not specified.
(DOCX)

S5 Table Characteristics of patients included in adjusted Poisson model versus those in full sample (bariatric surgery as outcome). B&A, Black and Asian ethnic groups; BMI, body mass index. ^a^Included in adjusted model. ^b^Medical codes indicating diagnosis with severe and complex obesity where BMI category not specified.
(DOCX)

S1 Fig Percentage of adults with overweight or obesity in England as recorded in CPRD GOLD. ^**a**^**, by (a) sex, (b) age category**^**b**^**, and (c) region**^**c**^. ^a^Includes data from January 2007 until June 2020. ^b^Age at end of study period (earliest of: death, transfer out from practice, end of practice data collection). ^c^East Midlands and North East drop out of the dataset after 2015 and 2017, respectively.
(DOCX)

S2 Fig **Percentage of adults with overweight and obesity in England with a WM referral recorded in CPRD GOLD**^**a**^ **by (a) sex, (b) age category**^**b**^**, and (c) region**^**c**^. ^a^Includes data from January 2007 until June 2020. ^b^Age at WM referral. ^c^East Midlands and North East drop out of the dataset after 2015 and 2017, respectively.
(DOCX)

S3 Fig **Percentage of adults with severe and complex obesity in CPRD GOLD.** ^**a**^**Who underwent NHS bariatric surgery in England by (a) sex, (b) age category**^**b**^**, and (c) region**^**c**^. ^a^Includes data from January 2007 until June 2020 in adults eligible for Hospital Episode Statistics linkage. ^b^Age at bariatric surgery. ^c^East Midlands, North East, and East of England drop out of the dataset after 2015, 2017, and 2019, respectively.
(DOCX)

## Abbreviations

BMI: body mass index
CCG: Clinical Commissioning Group
CHD: coronary heart disease
CI: confidence interval
CPRD: Clinical Practice Research Datalink
ESS: Enhanced Service Specification
GORD: gastro-oesophageal reflux disease
GP: general practitioner
HSE: Health Survey for England
HES-APC: Hospital Episode Statistics–Admitted Patient Care
HTN: hypertension
IIH: idiopathic intracranial hypertension
IMD: Index of Multiple Deprivation
IQR: interquartile range
ISAC: Independent Scientific Advisory Committee
NBSR: National Bariatric Surgery Registry
NICE: National Institute for Health and Care Excellence
OSA: obstructive sleep apnoea
PCOS: polycystic ovary syndrome
PPI: patient and public involvement
QOF: Quality Outcomes Framework
RR: rate ratio
T2DM: type 2 diabetes mellitus
WM: weight management
