# Supplementary material for: Access to publicly funded weight management services in England using routine data from primary and secondary care (2007–2020): An observational cohort study
Source: PLoS Med. 2023 Sep 28;20(9):e1004282. doi: 10.1371/journal.pmed.1004282 (PMC10538857; doi:10.1371/journal.pmed.1004282)
Supplement: S1 Checklist — (DOCX) [file pmed.1004282.s001.docx]

**S1 Checklist: STROBE (Strengthening the Reporting of Observational Studies in Epidemiology) and-RECORD (Reporting of studies Conducted using Observational Routinely-collected health Data (RECORD) statements [32]**

| **Checklist Item** | **Location in Manuscript and example text** |
| --- | --- |
| **Title and Abstract** |  |
| 1. (a) Indicate the study’s design with a commonly used term in the title or the abstract.  (b) Provide in the abstract an informative and balanced summary of what was done and what was found. (STROBE)  RECORD 1.1: The type of data used should be specified in the title or abstract. When possible, the name of the databases used should be included.  RECORD 1.2: If applicable, the geographic region and time frame within  which the study took place should be reported in the title or abstract.  RECORD 1.3: If linkage between databases was conducted for the study, this should be clearly stated in the title or abstract. | **(a) *Title*:** “Access to publicly funded weight management services in England (2007-2020): Cohort study using routine data from primary and secondary care.”  **(b) See abstract.**  **RECORD 1.1 & 1.2 & 1.3:** ***Title*:** “…using routine data from primary and secondary care.”  ***Abstract*:** “An observational cohort study was undertaken using routinely collected primary care data in England from the Clinical Practice Research Datalink linked with Hospital Episode Statistics. During the study period (January 2007-June 2020)…” |
| **Introduction** |  |
| 2. Explain the scientific background and rationale for the investigation being reported. (STROBE) | **See Introduction pages 4-5. Example text Introduction, paragraph 3:** “It is not known whether intervention rates have improved in the last 10 years. A recent systematic review investigated inequalities in the uptake, adherence to and effectiveness of behavioural WM interventions within trial settings and found that most trials did not examine whether inequalities occurred [24]. To our knowledge no systematic review has investigated inequalities in WM referrals within routine clinical practice.” |
| 3. State specific objectives, including any prespecified hypotheses. (STROBE) | **Introduction, paragraph 5 for study aims:** “The aims of this study were to: 1) Describe the population of adults with a recording of overweight or obesity in primary care in England, including those who a) receive an NHS referral for WM from primary care and b) undergo NHS bariatric surgery, 2) Determine the patient and GP practice characteristics associated with a) WM referral and b) bariatric surgery.”  **Methods, ‘Study design’** **for specific research questions:** “Our research questions were: 1) Of people with overweight and obesity in primary care in England (eligible population), who and how many are a) referred for WM and b) undergo publicly funded bariatric surgery (in the sub-group with severe and complex obesity)? 2) For people who are eligible, which patient and GP-practice factors are associated with WM referral and bariatric surgery?” |
| **Methods** |  |
| 4. Present key elements of study design early in the paper. (STROBE) | **Methods, ‘Study design’** : “An observational cohort study design was undertaken using primary care data from CPRD of all adult patients in England with a record indicating overweight or obesity. The study period ran from January 2007 (to coincide with the publication of the first NICE guidance for obesity in December 2006) to June 2020 (the latest CPRD update available when the data were extracted).” |
| 5. Describe the setting, locations, and relevant dates, including periods of recruitment, exposure, follow-up, and data collection. (STROBE) | **Methods, ‘Study design’ (see text in item 4. Above).**  **Methods, ‘Follow-up’:** “Individuals included in the study were followed up from first indication of overweight/obesity until the earliest of the following: first indication of WM referral or bariatric surgery, death, transfer out from practice; end of practice data collection; or end of the study period (June 2020).” |
| 6. (a) *Cohort study:* Give the eligibility criteria and the sources and methods of selection of participants. Describe methods of follow-up.  (b) *Cohort study:* For matched studies, give matching criteria and number of exposed and  unexposed. (STROBE)  RECORD 6.1: The methods of study population selection (such as codes or algorithms used to identify subjects) should be listed in detail. If this is not possible, an explanation should be provided.  RECORD 6.2: Any validation studies of the codes or algorithms used to select the population should be referenced. If validation was conducted for this study and not published elsewhere, detailed methods and results should be provided.  RECORD 6.3: If the study involved linkage of databases, consider use of a flow diagram or other graphical display to demonstrate the data linkage process, including the number of individuals with linked data at each stage. | **(a) Methods, ‘Participants and inclusion criteria’, and Table 1 (exposure definitions). Example text Methods:** “The source population included all patients with ‘research quality’ data (deemed ‘acceptable’ quality by CPRD and providing some ‘up-to-standard' data), registered with general practices between January 2007 and June 2020 contributing to the CPRD GOLD primary care dataset [22]. Adults aged ≥18 years who were registered at GP practices in England were included in the study if during the study period they had a recording of overweight or obesity (BMI ≥25.0 or ≥23.0 kg/m^2^ in people of Black and Asian ethnic groups as defined in the 2014 NICE obesity guidance) in CPRD GOLD [11] (**Table 1**)”  See item 5 for example text for methods of follow-up (**Methods, ‘Follow-up’**).  **(b) N/A**  **RECORD 6.1:** **Detailed in Table 1 (Exposure definitions), S2 Appendix (code lists for Exposures and Outcomes) and S3 Appendix (code lists for co-variables).**  **RECORD 6.2**: **Methods, ‘Outcomes’**: “KC (obesity specialist dietitian) compiled the code lists for the exposures and outcomes which were cross-checked by HP (GP with specialist interest in obesity). Previously published bariatric surgery procedure codes (cross checked by RW, bariatric surgeon) were used to identify bariatric surgery [34].”  **Methods, ‘Co-variables’**: “Co-variables included…ethnicity (identified using both clinical codes within CPRD and patient information within HES mapped into six ethnicity categories as per Mathur et al [35])…smoking status (earliest after index date, not censored for outcome; identified from clinical codes and Additional Clinical Details file in CPRD and classified into current, ex- and never/non-smoker), BMI category (as defined in the 2014 NICE obesity guidance [11]) at diagnosis with overweight/obesity (or severe and complex obesity)…Both clinical codes within CPRD and ICD-10 codes within HES were used to identify co-morbidities where possible to improve completeness of data. Existing publicly available code lists were used where possible.”  Previously used code list references included in S2 and S3 Appendices.  **RECORD 6.3**: Figure 1. |
| 7. Clearly define all outcomes, exposures, predictors, potential confounders, and effect modifiers. Give diagnostic criteria, if applicable. (STROBE)  RECORD 7.1: A complete list of codes and algorithms used to classify exposures, outcomes, confounders, and effect modifiers should be provided. If these cannot be  reported, an explanation should be provided. | **Methods, paragraphs 4-6**  **RECORD 7.1**: Table 1 (Exposure definitions), S2 Appendix (code lists for Exposures and Outcomes) and S3 Appendix (code lists for co-variables). |
| 8. For each variable of interest, give sources of data and details of methods of assessment (measurement). Describe comparability of assessment methods if there is more than one group. (STROBE) | **Methods, paragraphs 4-6. Table 1 (Exposure definitions)** |
| 9. Describe any efforts to address potential sources of bias. (STROBE) | **Methods, ‘Statistical analyses’:** “The characteristics of individuals included in the full model versus those with complete data across all variables included in the adjusted model were compared to examine the potential for information and confounding bias. We also conducted a sensitivity analysis removing people with ‘Unknown’ ethnicity from the adjusted models.” |
| 10. Explain how the study size was arrived at. (STROBE) | **Figure 1 (flow diagram)** for size of sample for each analysis**.**  ***To identify the study population -* Methods ‘Participants and inclusion criteria’**: “The source population included all patients with ‘research quality’ data (deemed ‘acceptable’ quality by CPRD and providing some ‘up-to-standard' data), registered with general practices between January 2007 and June 2020 contributing to the CPRD GOLD primary care dataset [22]. Adults aged ≥18 years who were registered at GP practices in England were included in the study if during the study period they had a recording of overweight or obesity (BMI ≥25.0 or ≥23.0 kg/m^2^ in people of Black and Asian ethnic groups as defined in the 2014 NICE obesity guidance) in CPRD GOLD [11]…” |
| 11. Explain how quantitative variables were handled in the analyses. If applicable, describe which groupings were chosen and why. (STROBE) | **Methods ‘Outcomes’ and ‘Co-variables’:**  **Example text, ‘Outcomes’: “**Relevant clinical codes were used to define whether an individual received a WM referral (YES/NO) (outcome a).”  **Example text, ‘Co-variables:** “Co-morbidities were included in the analysis models in two different ways; as presence of individual co-morbidities (13 individual binary YES/NO co-morbidity variables, model A) and as a total number of co-morbidities (a single categorical variable, model B).” |
| 12. (a) Describe all statistical methods, including those used to control for confounding.  (b) Describe any methods used  to examine subgroups and interactions.  (c) Explain how  missing data were addressed.  (d) *Cohort study:* If applicable, explain how loss to follow-up was addressed.  (e) Describe any sensitivity analyses. (STROBE)  RECORD 12.1: Authors should describe the extent to which the investigators had access to the database population used to create the study population.  RECORD 12.2: Authors should provide information on the data cleaning methods used in the study.  RECORD 12.3: State whether the study included person-level, institutional-level, or other data linkage across two or more databases. The methods of linkage and methods of linkage quality evaluation should be provided. | **(a) Methods, ‘Statistical analyses’, example text:** “The characteristics of individuals included in the full model versus those with complete data across all variables included in the adjusted model were compared to examine the potential for information and confounding bias.”  **(b) N/A**  **(c)** **Methods, ‘Statistical analyses’:** “Some data were missing for IMD, smoking and Rural-Urban index of GP practice. Additionally, for ethnicity, 16.5% of individuals included in the study were coded as 'Unknown' ethnicity. Missing or ‘Unknown’ data for these co-variables were not imputed as they are probably not missing at random [36,37]. The characteristics of individuals included in the full model versus those with complete data across all variables included in the adjusted model were compared to examine the potential for information and confounding bias. We also conducted a sensitivity analysis removing people with ‘Unknown’ ethnicity from the adjusted models.”  **(d) N/A**  **(e) Methods, ‘Statistical analyses’:** “We also conducted a sensitivity analysis removing people with ‘Unknown’ ethnicity from the adjusted models.” |
| **Results** |  |
| 13. (a) Report the numbers of individuals at each stage of the study (e.g., numbers potentially eligible, examined for eligibility, confirmed eligible, included in the study, completing follow-up, and analysed).  (b) Give reasons for non-participation at each stage.  (c) Consider use of a flow  diagram. (STROBE)  RECORD 13.1: Describe in detail the selection of the persons included in the study (i.e., study population selection), including filtering based on data quality, data availability, and linkage. The selection of included persons can be described in the text and/or by means of the study flow diagram. | **(a) (b) (c) See Figure 1 – flow diagram.**  **RECORD 13.1: See Figure 1 – flow diagram.** |
| 14. (a) Give characteristics of study participants (e.g., demographic, clinical, and social) and information on exposures and potential confounders.  (b) Indicate the number of participants with missing data for each variable of interest.  (c) *Cohort study:* summarise follow-up time  (e.g., average and total amount). (STROBE) | **(a) Results paragraphs 1-3 ‘Participants and descriptive information’ and Tables 2, 3 and S1 Table.**  **(b) Provided in Tables 2, 3 and S1 Table.**  **(c) Results paragraph 2 ‘Weight management referrals’:** “The median study follow-up time from date of diagnosis with overweight/obesity (index date) was 7.18 years, interquartile range (IQR) 3.47-10.70 years.”  **Results paragraph 3 ‘Bariatric surgery’:** “The median follow-up time from severe and complex obesity index date was 6.85 years (IQR 3.28-10.51).” |
| 15. *Cohort study:* Report numbers of outcome events or summary measures over time. (STROBE) | **Results paragraph 2 ‘Weight management referrals’:** “During the study period, 56,783/1,811,587 adults had a recorded WM referral equating to just 3.13% of adults living with overweight or obesity within the study (**Table 2**).”  **Results paragraph 3 ‘Bariatric surgery’:** “Of the 340,502 eligible for HES linkage, 3,701 (1.09%) underwent NHS bariatric surgery (**Table 3**).” |
| 16. (a) Give unadjusted estimates and, if applicable, confounder-adjusted estimates and their precision (e.g., 95% confidence interval). Make clear which confounders were adjusted for and why they were included.  (b) Report category boundaries when continuous variables were categorized.  (c) If relevant, consider translating estimates  of relative risk into absolute risk for a meaningful time period. (STROBE) | **(a) Both unadjusted and adjusted Rate Ratios with 95% CIs provided Tables 4 and 5 and S2 and S3 Tables.**  **(b) N/A**  **(c) N/A** |
| 17. Report other analyses done—e.g., analyses of subgroups and interactions and sensitivity analyses. (STROBE) | **Model B (with total number of co-morbidities included instead of individual co-morbidities) presented in Results, paragraph 10 ‘Model B’ and in S2 and S3 Tables. Results of sensitivity analyses presented Results paragraph 11 ‘Sensitivity analysis’ and S4 and S5 Tables.** |
| **Discussion** |  |
| 18. Summarise key results with reference to study objectives. (STROBE) | **Discussion paragraph 1** |
| 19. Discuss limitations of the study, taking into account sources of potential bias or imprecision. Discuss both direction and magnitude of any potential bias. (STROBE)  RECORD 19.1: Discuss the implications of using data that were not created or collected to answer the specific research question(s). Include discussion of misclassification bias, unmeasured confounding, missing data, and changing eligibility over time, as they pertain to  the study being reported. | **Discussion, paragraph 7**  **RECORD 19.1: Discussion paragraph 7:** “A weakness of all studies using routinely collected primary care data is reliance on clinical data being recorded in patients’ primary care records. This may have introduced selection bias in this study as those living with overweight or obesity who have a diagnosis documented in their GP records may be different in some way to those who do not.” |
| 20. Give a cautious overall interpretation of results considering objectives, limitations, multiplicity of analyses, results from similar studies, and other relevant evidence. (STROBE) | **Discussion paragraphs 8-9** |
| 21. Discuss the generalisability (external validity) of the study results. (STROBE) | **Discussion paragraph 7:** “A weakness of all studies using routinely collected primary care data is reliance on clinical data being recorded in patients’ primary care records. This may have introduced selection bias in this study as those living with overweight or obesity who have a diagnosis documented in their GP records may be different in some way to those who do not. … Another limitation is that the Vision practice software which CPRD GOLD draws on has been declining in use in recent years, and thus it is possible there could be an issue of reducing representativeness of practices in CPRD GOLD over time. This study only captures WM referrals made from primary care and was not able to capture patient self-referrals, or referrals from other non-primary care-based health and social care professionals which is possible for some community-based NHS WM services. We used a focused definition of WM referral, to ensure that we correctly identified referrals for WM in line with the NICE guidance. It is possible we missed some programmes which include a WM component but may not be coded as such in primary care.” |
| 22. Give the source of funding and the role of the funders for the present study and, if applicable, for the original study on which the present article is based. (STROBE)  RECORD 22.1: Authors should provide information on how to access any supplemental information such as the study protocol, raw data, or programming code. | **Please see funding statement.**  **RECORD 22.1: Protocol provided in S1 Appendix.** |
