# Supplementary material for: Access to publicly funded weight management services in England using routine data from primary and secondary care (2007–2020): An observational cohort study
Source: PLoS Med. 2023 Sep 28;20(9):e1004282. doi: 10.1371/journal.pmed.1004282 (PMC10538857; doi:10.1371/journal.pmed.1004282)
Supplement: S1 Appendix — ISAC, Independent Scientific Advisory Committee. (PDF) [file pmed.1004282.s002.pdf]

INDEPENDENT SCIENTIFIC ADVISORY COMMITTEE (ISAC) PROTOCOL APPLICATION FORM

PART 2: PROTOCOL INFORMATION

Applicants must complete all sections listed below<br>Applications with sections marked 'Not applicable' without justification will be returned as invalid
**A. Study Title (Max. 255 characters, including spaces)**<br><br>A cohort study examining access to NHS weight management services using routine data from primary and secondary care
**B. Lay Summary (Max. 250 words)**<br><br><u>Background</u><br><br>Obesity is a major health issue associated with many health problems costing the National Health Service (NHS) billions. Public health programmes are very important to prevent overweight and obesity but for those who are already living with obesity, effective treatments are needed to improve health. Weight management (WM) services available in the NHS include community-based lifestyle programmes, specialist medical WM services, and surgical treatment (bariatric surgery) for those living with the most severe and complex obesity. These NHS services, however, are not consistently available in all areas of the country, and the number of bariatric surgery operations undertaken nationally is lower than other European countries. We know that not everyone who is eligible for NHS WM services are able to access them, but we don't know which groups of people are more likely to get access.<br><br><u>Purpose of the study</u><br>This study will 1) describe the characteristics of people with overweight and obesity in England, and 2) determine which groups within this population are more/less likely to get access to NHS WM services including bariatric surgery.<br><br><u>Potential importance of the findings</u><br><br>Given the current obesity problem, its associated health risks and healthcare costs, it is important that strategies to reduce overweight and obesity levels are available and delivered. This research will provide information about which groups are most under-served by NHS WM services. This will help to inform future planning of services to improve fairness of access.

C. Technical Summary (Max. 300 words)

<u>Aims and Objectives</u>
This study aims to describe the characteristics of people with overweight and obesity in England who are eligible for NHS weight management (WM) services including bariatric surgery, according to national guidance, and determine predictors of access to these services. We will also investigate trends in bariatric surgery before and after the transfer of commissioning responsibility from NHS England to Clinical Commissioning Groups in April 2017.

<u>Exposures and Outcomes</u>
The primary exposure is people with a diagnosis of overweight and obesity in England during the study period (January 2007-latest CPRD update), and the outcomes are **a)** referral to NHS WM services and **b)** undergoing NHS bariatric surgery.

<u>Methods</u>
This study includes both descriptive and hypothesis-testing elements using retrospective cohort and cross-sectional designs. Hospital Episode Statistics (HES) will be used to cross-check bariatric surgery recorded in CPRD.

<u>Data analyses</u>
Descriptive statistics will summarise demographic and clinical characteristics of the overall study cohort. The provision to need ratios for outcomes **a)** and **b)** will be descriptively calculated (number within the cohort with each outcome compared with the total number eligible for these services according to national guidance), and stratified by demographic and clinical characteristics. Proportions and Confidence Intervals will be calculated using a Poisson model. Cox regression will estimate time to event data including predictors for both outcomes **a)** and **b)**. An interrupted time series approach will investigate the trend in bariatric surgery levels before and after the transfer of commissioning responsibility. Segmented linear regression will estimate changes in levels and trends after the transfer. Cox regression will be repeated on two separate cohorts of patients – a pre-intervention and post-intervention cohort.

D. Outcomes to be Measured

a) Referral to weight management services (primary care-based or hospital-based)
b) Undergoing bariatric surgery

E. Objectives, Specific Aims and Rationale

Objective:
To describe pathways of access to NHS weight management services in people with overweight and obesity and determine the patient and GP practice factors associated with an increased likelihood of referral to these services.

Specific aims:

1) Identify and describe the demographic and clinical characteristics of people with a diagnosis of overweight and/or obesity in primary care in England since 2007 (the publication of the first National Institute for Health and Care Excellence (NICE) guidance for obesity)
2) In people with a diagnosis of overweight and obesity, identify the number of a) referrals for NHS weight management services and b) primary NHS bariatric surgery operations carried out in England and compare these with the number who would be eligible for these interventions according to NICE guidance
3) Identify patient and GP practice predictors of time to a) referral to NHS weight management services, and b) treatment with bariatric surgery
4) Compare trends in bariatric surgery before and after April 2017 (transfer of commissioning responsibility for bariatric surgery from NHS England to Clinical Commissioning Groups (CCGs)

Rationale:
In England, 63% of the adult population are living with overweight or obesity (body mass index (BMI) ≥25 kg/m^2), and 3% live with severe obesity (BMI ≥40 kg/m^2) (1). Excess weight is associated with a multitude of health problems which cost the NHS billions (2, 3). Effective interventions are needed to both prevent overweight and obesity and treat those currently living with obesity. A tiered approach to NHS weight management (WM) services exists in England including tier 1 (population health preventative interventions), tier 2 (lifestyle WM services), tier 3 (specialist WM services) and tier 4 (bariatric surgery). Not all areas of the country have access to NHS tier 2 or 3 WM services and there is no national registry of these services, meaning little is known about who accesses them and how this compares to NICE guidance. Previous research, however, suggests that access to primary care WM interventions is low (4). Bariatric surgery (tier 4) is a NICE recommended clinical and cost-effective treatment for severe and complex obesity (5-7). However, access to and provision of bariatric surgery is limited in the UK (8).

Given the scale of the current obesity problem, and its associated disease burden and healthcare costs, it is essential that strategies to reduce overweight and obesity levels are available and delivered. Little is known about the factors influencing access to WM services; however, this information is crucial to improve the equity of accessing effective care for people with overweight and obesity. This study will investigate pathways of access to NHS WM services in people with overweight and obesity and determine the patient and GP practice factors associated with an increased likelihood of referral to these services.

F. Study Background

Over 1.9 billion adults worldwide suffer from overweight and obesity, with rates of obesity tripling since 1975 (9). In England, 63% of the adult population are living with overweight or obesity (1). Excess weight is associated with an increased risk of type 2 diabetes, cardiovascular disease, certain cancers, depression, reduced quality of life, and premature death (10-15). In 2006-7, the cost of overweight and obesity to the NHS was estimated at £5.1 billion, and by 2050, is forecast to be £9.7 billion (2, 3). Effective public health initiatives are critically important to prevent future overweight and obesity, however, experts agree these are not sufficient to achieve weight loss in those already suffering with obesity, particularly those with severe and complex obesity (BMI of ≥40 kg/m^2, or 35-40 kg/m^2 with another significant health problem that could be improved by weight loss), who are at the highest risk of morbidity and premature death (1, 16, 17). In 2018, 3% of adults in England were reported to have a BMI ≥40 kg/m^2, and data from the USA indicate that 40% of the total healthcare costs of overweight and obesity can be attributed to the 8% of the US population with a BMI ≥35 kg/m^2 (1, 16, 18). Thus, government-supported public health initiatives are urgently needed to prevent people living with overweight and obesity as well as effective clinical interventions for those who already living with obesity, to reduce associated morbidity and healthcare costs (16).

In England, there is a tiered pathway of WM services in the NHS, which include population-wide strategies to reduce overweight and obesity (tier 1), lifestyle WM services (tier 2) which can include group-based interventions and/or individual support from community-based health professionals, and 'tier 3' specialist multi-disciplinary WM services (often based in secondary care) (17, 19, 20). The National Institute for Health and Care Excellence (NICE) recommends NHS-funded lifestyle WM services for people with a BMI ≥30 kg/m² and ≥25 kg/m² where capacity, although lower BMIs can be considered for people of certain minority ethnic groups who are at an increased risk of developing weight-related co-morbidities (21, 22). Tier 3 multidisciplinary specialist services are recommended for people with severe and complex obesity (22, 23). From tier 3, there is the opportunity where appropriate to be referred into 'tier 4' for assessment of bariatric surgery (8). Bariatric surgery is the most clinically effective treatment when combined with behaviour change and dietary management in people with severe and complex obesity (5). Comparative studies show consistently that bariatric surgery leads to greater weight loss and improvement in control of type 2 diabetes, compared with lifestyle interventions or drug therapy alone, and is also cost-effective (5-7). NICE recommends bariatric surgery and appropriate long-term behavioural and dietary support be considered for people with severe and complex obesity, that have been unable to achieve adequate or sustained weight loss through non-surgical interventions alone (17). In addition, it can be considered as a first line treatment for those with a BMI ≥50 kg/m². NICE also recommends that bariatric surgery can be considered for people at lower BMIs with recent-onset type 2 diabetes mellitus.

Not all regions in England have access to tier 2 and 3 WM services and some areas are known to set variable referral criteria (different to NICE recommendations) for patients to move between tier 2, 3 and 4 services. The number of referrals made to tiers 2, 3 and 4 services nationally is not known, as currently there is no national registry for tier 2 and 3 services (23, 24). Previous research undertaken in CPRD between 2005 and 2012 reported low access to primary care WM interventions, which included brief GP advice, prescriptions for WM drugs, and referrals to WM services (4). Approximately 60% of people with a BMI ≥40 kg/m² did not have any WM intervention recorded during the study period, and this rose to 90% in those with a BMI 25-29.9 kg/m². In terms of WM referrals specifically, only around 17% of people with a BMI ≥40 kg/m² received a referral, dropping to 3% in those with a BMI 25-29.9 kg/m². BMI category, female gender, increasing age, socioeconomic deprivation and the presence of heart disease, type 2 diabetes, and depression were associated with greater access to WM interventions overall, however, predictors of receiving a WM referral specifically were not reported. Bariatric surgery rates in the UK are low compared with other European countries and are far below clinical need (8, 25). Rates and clinical outcomes of bariatric surgery have previously been investigated in CPRD studies, however to our knowledge there is no data on which groups are more likely to undergo bariatric surgery within the eligible population (26-29).

There have been some important changes in the landscape since the above research on primary care WM interventions and bariatric surgery was conducted by Booth *et al* (4). The Quality and Outcomes Framework (QOF) is a voluntary incentive programme that financially rewards GP surgeries in England for good practice (30). In 2015/16 "establishing and maintaining a register of patients aged ≥18 years who have a recorded BMI of ≥30 within the previous 12 months" was incorporated as an indicator into QOF for which GP surgeries could receive points (31). The impact of the incorporation of this indicator into QOF on access to WM services is not known. Another development was the transfer of commissioning responsibility for bariatric surgery from NHS England to CCGs in April 2017 (32). There has been concern that this will have had an impact on patient access to bariatric surgery, however, the actual impact of this change is not known (33).

G. Study Type

A descriptive study will be undertaken for aims 1 and 2 summarising the demographic and clinical characteristics of people with overweight and obesity (aim 1), and the number referred for WM services and bariatric surgery compared with the number eligible for these services according to NICE guidance (aim 2). A hypothesis testing study will be used for aims 3 and 4 to investigate whether WM treatment pathways differ according to patient and GP-practice characteristics (aim 3), and whether there is any difference in bariatric surgery trends before and after the transfer of commissioning responsibility for bariatric surgery from NHS England to individual CCGs in April 2017 (aim 4).

H. Study Design

This study will use both cross-sectional (aims 1 and 2) and retrospective cohort (aims 3 and 4) designs to address the research aims.

I. Feasibility counts

Counts in CPRD GOLD (prevalent cases):

- Patients with a diagnosis of overweight/obesity during the study period with 1 year of up-to-standard registration before index date: 2,332,949
- Having bariatric surgery after index date: 4,158
- Having WM referral after index date: 57,262
- Patients in above cohort with index BMI > 40: 122,617
- BMI > 40 and bariatric surgery: 2,494
- BMI > 40 and WM referral: 11,535

Counts limited to incident cases (first ever diagnosis in study period) in CPRD GOLD:

- Patients with a **first ever** diagnosis of overweight/obesity during the study period with 1 year of up-to-standard registration before index date: 1,052,460
- Having bariatric surgery after index date: 1,885
- Having WM referral after index date: 48,510
- Patients in above cohort with index BMI > 40: 16,911
- BMI > 40 and bariatric surgery: 141
- BMI > 40 and WM referral: 971

J. Sample size considerations

Sample size calculations are based on feasibility counts of **incident** cases (see section I.)

Weight management referrals as outcome:

A previous study by Booth *et al* (4) reported the "hazard" of being referred to a WM service in the obesity class 1 group compared to the overweight group to be 2.12. Based on this estimate, assuming 80% power, alpha 0.05, and an overall event rate of 4.6% (48,510/1,052,460 based on our feasibility counts in CPRD GOLD – see section I), we would need a sample size of **1209** in our study to estimate the same effect.

Bariatric surgery as outcome:

A previous study by Booth *et al* (26) reported the "hazard" of undergoing bariatric surgery in women with obesity aged 35-54 years compared to women with obesity aged 20-34 to be 3.1. Based on this estimate, assuming 80% power, alpha 0.05, and an event rate of 0.18% in the total cohort (1885/1,052,460 based on our feasibility counts in CPRD GOLD), we would need a sample size of **13,626** in our study to estimate the same effect.

However, we will be limiting the bariatric surgery analysis to people within the overall cohort who meet the NICE criteria for bariatric surgery (BMI ≥40 kg/m² or BMI 30-40 kg/m² with a relevant co-morbidity, **see section L**). Our feasibility counts show that in people with BMI ≥40 kg/m², the event rate is 0.83% (141/16,911). Using this event rate, we would need a sample size of **2,955**.

Our feasibility counts in CPRD GOLD indicate there are **1,052,460** patients (incident cases) who meet the study inclusion criteria (BMI ≥25 kg/m²), and **16,911** within this cohort with a BMI ≥40 kg/m². Thus, our study will be very well-powered for both outcomes (using either event rate for bariatric surgery and anything in between). Analyses using prevalent cases will be even more well-powered (**2,332,949** patients who meet the inclusion criteria, **122,617** with BMI ≥40).

K. Planned use of linked data (if applicable):

We will require access to HES Admitted Patient Care data to include ICD-10 diagnostic codes for obesity and OPCS-4 procedure codes for bariatric surgery to validate the recording of bariatric surgery in CPRD. We will also require linkage to the Index of Multiple Deprivation (patient-level), and Rural-Urban classification (practice-level), to investigate whether variations in socioeconomic status and rurality influence access to weight management services.

Patient benefit
Data from this study will provide important information on inequalities in access to weight management services. This data will be useful for policy makers in commissioning and designing services to improve equity of care. This work is part of a larger project investigating barriers to accessing weight management services in people living with obesity which will lead to improved knowledge for health professionals and policymakers with regards to improving and streamlining weight management services for patients with obesity.

L. Definition of the Study population

Inclusion criteria

Patients who have been registered with a GP practice for at least one year will be included in the study if since January 2007 (the publication of the first NICE guidance for obesity) they have:

- A recorded BMI ≥25 kg/m^2 **OR**
- A recorded BMI ≥23 kg/m^2 in people of Black African, African-Caribbean and Asian ethnic groups **OR**
- A recorded diagnosis of overweight or obesity as per READ codes **AND**
- Are aged ≥18 years at the time of eligible BMI recording or diagnosis of overweight/obesity

Exclusion criteria

- Patients who have been registered with a GP practice for less than one year
- Patients that do not have an eligible BMI or diagnosis of overweight/obesity recorded within the study period (January 2007 – latest CPRD dataset)
- Patients who are <18 years of age at eligible BMI recording or diagnosis of overweight/obesity
- Patients who were recorded to have undergone bariatric surgery prior to diagnosis with overweight/obesity.

For the analyses relating to bariatric surgery, people with **severe and complex obesity** (potentially eligible for bariatric surgery according to NICE guidance) defined as:

- A recorded BMI ≥40 kg/m^2 **OR**
- BMI 35-39.9 kg/m^2 **AND** one of the following recorded co-morbidities:
    - Type 2 diabetes
    - Hypertension
    - Coronary heart disease
    - Obstructive sleep apnoea
    - Asthma
    - Chronic musculo-skeletal problem on daily medication (*such as OA, back pain, knee pain, exclude those with inflammatory conditions only*)
    - Gastro-oesophageal reflux disease (GORD)
    - Liver disease
    - Polycystic ovary syndrome (PCOS)
    - Fertility problems
    - Depression
    - Anxiety
    - Idiopathic intracranial hypertension **OR**
- BMI 30-34.9 kg/m^2 **AND** type 2 diabetes diagnosed within the last 10 years **OR**
- BMI 27.5-34.9 kg/m^2 in people of Black African, African-Caribbean and Asian ethnic groups **AND** type 2 diabetes diagnosed within the last 10 years **OR**
- A recorded diagnosis of severe obesity as per READ codes

M. Selection of comparison group(s) or controls

No control population is required for this study. All analyses (detailed in section O.) will be undertaken within the study population (people with overweight and obesity).

N. Exposures, Outcomes and Covariates

Aim 1

Population: People aged ≥18 years of age in England who have been registered with a GP practice for at least one year.

Outcome: People with a diagnosis of overweight or obesity in primary care, defined as a recorded BMI ≥25 kg/m² or a diagnosis of overweight or obesity as per READ codes since January 2007.

Covariates: Demographics: Age, gender, ethnicity, level of deprivation of patient, geographical region of GP practice by strategic health authority, rural-urban classification of GP practice.

Clinical characteristics: BMI, smoking status, co-morbidities (Type 2 diabetes including date of diagnosis, hypertension, coronary heart disease, obstructive sleep apnoea, asthma, chronic musculoskeletal pain on daily medication, gastro-oesophageal reflux disease, liver disease, polycystic ovarian syndrome, fertility problems, depression, anxiety, idiopathic intracranial hypertension)

Aim 2

a: *Population:* People with a diagnosis of overweight or obesity in primary care (as defined above) since January 2007.

Outcome: Referred for NHS WM services (to include community-based and/or secondary care-based services) (YES/NO) after first eligible BMI measurement or diagnosis of overweight/obesity up until the latest CPRD update.

b: *Population:* People within the cohort identified in a. with severe and complex obesity (potentially eligible for bariatric surgery according to NICE criteria, see definition in section L).

Outcome: Underwent NHS bariatric surgery (YES/NO) after first eligible BMI measurement or diagnosis of severe obesity up until the latest CPRD update.

Covariates: As per aim 1

Aim 3

a: *Exposure:* People with a diagnosis of overweight or obesity in primary care (as defined above) since January 2007.

Outcome: Patient and GP-practice predictors of time to referral to NHS WM services from first eligible BMI measurement or diagnosis of overweight/obesity up until the latest CPRD update.

b: *Exposure:* People within the cohort identified in a. with severe and complex obesity (potentially eligible for bariatric surgery according to NICE criteria).

Outcome: Patient and GP-practice predictors of time to treatment with bariatric surgery from **i.** first eligible BMI measurement or diagnosis of severe obesity, and **ii.** first referral to NHS WM services up until the latest CPRD update.

Covariates: As per aim 1

Aim 4

Exposure: Time – April 2017 (the transfer of commissioning responsibility for bariatric surgery from NHS England to CCGs).

Outcome: Trends in bariatric surgery levels

Covariates: As per aim 1

O. Data/ Statistical Analysis

Aim 1: Descriptive statistics will be used to describe the demographic and clinical characteristics of people with overweight and obesity in primary care in England since 2007 (the publication of the first NICE guidance for obesity). Means, standard deviations (SD), medians and inter-quartile ranges (IQR) will be reported by BMI group, demographic group, and by year.

Aim 2a: The provision to need ratio will be descriptively calculated for NHS WM services, with the numerator the number of people with overweight and obesity who have been referred to WM services, and the denominator the total number of people with overweight and obesity who would be eligible for referral to WM services according to NICE guidance.

2b: The provision to need ratio will be descriptively calculated for bariatric surgery, with the numerator the number of people with severe and complex obesity (as previously defined in section L) who underwent NHS bariatric surgery, and the denominator the total number of people with severe and complex obesity (potentially eligible for bariatric surgery according to NICE guidance) in primary care.

The provision to need ratios for **a.** and **b.** will be stratified by year (up to the latest update of CPRD), demographic and clinical characteristics. Rate Ratios and Confidence Intervals will be calculated using a Poisson model.

Aim 3: Cox regression will be used to estimate time to event data including predictors. Outcomes of interest include **a.** time to referral for WM services, and **b.** time to treatment with bariatric surgery. The proportional hazards assumption will be checked using Schoenfeld residuals. The incident date is the first eligible BMI recording or diagnosis of overweight or obesity after January 2007. Covariates to be included in the model include patient factors (age, gender, ethnicity, deprivation level, BMI, co-morbidities), GP-practice factors (rurality, geographical location according to SHA), and whether there is an NHS bariatric surgery centre located within the SHA). BMI will be treated as a time-varying exposure within the Cox model. Patients will be censored on date of the outcome of interest, date of death, date of loss to follow up, or end of study period.

Aim 4: An interrupted time series approach will be used to investigate the trend in bariatric surgery levels (outcome) before and after the transfer of commissioning responsibility for bariatric surgery in April 2017 (intervention). The number of bariatric surgery operations will be measured at monthly intervals over the study period (2007 through to latest CPRD update). Controlling for baseline level and trend, segmented linear regression will estimate changes in levels and trends after the change in commissioning responsibility. The regression model will include terms to estimate the pre-existing level for the outcome in the first month of the observation period (intercept), trend in the outcome before the intervention was introduced, change in level of the outcome after the intervention, and change in trend after the intervention. The model can cope in situations when the intervention takes time to affect the outcome (lag times), and in this situation we will consider taking that time period out of the analysis. We will include an extensive pre-intervention period to control for biases in level and trend at baseline. Cox regression will be used to obtain incidence rates for the outcome of time to treatment with bariatric surgery (as described in Aim 3). Analyses will be repeated on two separate cohorts of patients – a pre-intervention cohort and post-intervention cohort.

P. Plan for addressing confounding

A priori, we intend to adjust for all potential relevant confounders and covariates using the most recent recorded data after first eligible record of overweight/obesity and prior to the outcome of interest (WM referral/bariatric surgery) based on the available literature. We do not wish to make any decisions about accepting covariates that do not appear to influence the model in case these are in fact chance findings. We feel that given the size of the dataset there are sufficient numbers that this should not introduce any systematic bias to the model. We will consider the following covariates:

Patient-level: Age, gender, ethnicity, Index of Multiple Deprivation, BMI, smoking status, co-morbidities (Type 2 diabetes, hypertension, coronary heart disease, obstructive sleep apnoea, asthma, chronic musculoskeletal pain on daily medication, gastro-oesophageal reflux disease, liver disease, polycystic ovarian syndrome, fertility problems, depression, anxiety, idiopathic intracranial hypertension)

GP practice-level: Strategic health authority (SHA), Rural-Urban classification

Q. Plans for addressing missing data

Data from all patients with a first ever diagnosis of overweight and obesity in primary care during the study period will be included, and missing data will be managed using multiple imputation by chained equations. The cumulative effect of missing data in several variables often leads to exclusion of a substantial proportion of the original sample, causing a loss of precision and power. To overcome this bias multiple imputation allows for the uncertainty about missing data by creating several plausible imputed datasets and appropriately combining their results using Rubin’s rules. We include all covariates (as listed earlier) in the multiple imputation process, together with the outcome variable as this carries information about missing values of the predictors. The dataset will be scrutinised to determine the patterns and quantity of missing data and any reasons for, or associations with missingness. Potential auxiliary variables will be identified and incorporated into imputation models in order to reduce bias introduced by missing data. Sensitivity analyses will be conducted to determine if the estimates would have varied in case the assumption that the data are missing at random was violated.

R. Patient or user group involvement

A patient and public involvement (PPI) group consisting of people living with obesity and bariatric surgery are advising on a wider project to investigate barriers to accessing weight management services, of which this study is one part. Patient members are also part of the Expert Scientific Steering Group advising on this wider project. Patient advisors have specifically provided input on which covariates to collect within this study.

S. Plans for disseminating and communicating study results, including the presence or absence of any restrictions on the extent and timing of publication

We plan to publish our findings in a clinical peer-reviewed journal. We will also present our findings at relevant national and international clinical conferences, as well as national obesity advocacy group meetings.

Conflict of interest statement: We declare no conflicts of interest.

T. Limitations of the study design, data sources, and analytic methods

A previous CPRD study investigating access to primary care weight management interventions between 2005 and 2012 reported that only 45% of patients had a BMI recording, which may have introduced selection bias (4). It has been suggested that weight/BMI data are not likely to be missing at random and the probability of having weight recorded in primary care is associated with demographic and clinical factors (34, 35). Booth *et al* reported that people with higher BMIs were more likely to have their BMI recorded (36). However a study in CPRD between 1990-2011 noted that completeness of BMI records improved over time (35). BMI recording is likely to have improved further since this time due to the changes in QOF in 2015/16 to maintain a register of patients with a BMI ≥30 kg/m² (31). As shown by our feasibility counts and sample size calculations (sections I. and J.), there are more than enough patients who meet the inclusion criteria for our study (a diagnosis of overweight or obesity); the study will thus be well-powered. We will undertake sensitivity analyses to model different scenarios of under-reporting of BMI (34).

U. References

1. Lifestyles Team, NHS Digital. Health Survey for England 2018: Adult and child overweight and obesity. London, 2019. Available from: https://files.digital.nhs.uk/52/FD7E18/HSE18-Adult-Child-Obesity-rep.pdf. Date accessed: 6.5.20.
2. Scarborough P, Bhatnagar P, Wickramasinghe KK, Allender S, Foster C, Rayner M. The economic burden of ill health due to diet, physical inactivity, smoking, alcohol and obesity in the UK: an update to 2006-07 NHS costs. J Public Health (Oxf). 2011;33(4):527-35.
3. Butland B, Jebb S, Kopelman P, McPherson K, Thomas S, Mardell J, et al. FORESIGHT. Tackling Obesities: Future Choices - Project report, 2nd Edition. 2007.
4. Booth HP, Prevost AT, Gulliford MC. Access to weight reduction interventions for overweight and obese patients in UK primary care: population-based cohort study. BMJ Open. 2015;5(1):e006642.
5. Colquitt Jill L, Pickett K, Loveman E, Frampton Geoff K. Surgery for weight loss in adults. Cochrane Database Syst Rev. 2014(8). doi:10.1002/14651858.CD003641.pub4.
6. Picot J, Jones J, Colquitt JL, Gospodarevskaya E, Loveman E, Baxter L, et al. The clinical effectiveness and cost-effectiveness of bariatric (weight loss) surgery for obesity: a systematic review and economic evaluation. Health Technol Assess. 2009;13(41):1-+.
7. Gulliford MC, Charlton J, Prevost T, Booth H, Fildes A, Ashworth M, et al. Costs and Outcomes of Increasing Access to Bariatric Surgery: Cohort Study and Cost-Effectiveness Analysis Using Electronic Health Records. Value Health. 2017;20(1):85-92.
8. Welbourn R, le Roux CW, Owen-Smith A, Wordsworth S, Blazeby JM. Why the NHS should do more bariatric surgery; how much should we do? BMJ. 2016;353:i1472.
9. World Health Organisation. Obesity and overweight. In: WHO. 2020. Available from: https://www.who.int/news-room/fact-sheets/detail/obesity-and-overweight. Date accessed: 6.5.20.
10. Lim SS, Vos T, Flaxman AD, Danaei G, Shibuya K, Adair-Rohani H, et al. A comparative risk assessment of burden of disease and injury attributable to 67 risk factors and risk factor clusters in 21 regions, 1990–2010: a systematic analysis for the Global Burden of Disease Study 2010. Lancet. 2012;380(9859):2224-60.
11. Must A, Spadano J, Coakley EH, Field AE, Colditz G, Dietz WH. The disease burden associated with overweight and obesity. JAMA. 1999;282(16):1523-9.
12. Kopelman P. Health risks associated with overweight and obesity. Obes Rev. 2007;8:13-7.
13. Warkentin LM, Majumdar SR, Johnson JA, Agborsangaya CB, Rueda-Clausen CF, Sharma AM, et al. Predictors of Health-Related Quality of Life in 500 Severely Obese Patients. Obesity. 2014;22(5):1367-72.
14. Wang Y, McPherson K, Marsh T, Gortmaker SL, Brown M. Health and economic burden of the projected obesity trends in the USA and the UK. Lancet. 2011;378(9793):815-25.
15. Sarwer DB, Polonsky HM. The Psychosocial Burden of Obesity. Endocrinol Metab Clin North Am. 2016;45(3):677-88.
16. Dietz WH, Baur LA, Hall K, Puhl RM, Taveras EM, Uauy R, et al. Management of obesity: improvement of health-care training and systems for prevention and care. Lancet. 2015;385(9986):2521-33.
17. National Institute for Health and Care Excellence. Obesity: identification, assessment and management of overweight and obesity. Clinical Guideline [CG189]. London, 2014. Available from: https://www.nice.org.uk/guidance/cg189. Date accessed: 6.5.20.
18. Arterburn DE, Maciejewski ML, Tsevat J. Impact of morbid obesity on medical expenditures in adults. Int J Obes (Lond). 2005;29(3):334-9.
19. Public Health England. National mapping of weight management services: Provision of tier 2 and tier 3 services in England. London; 2015. Available from: https://www.gov.uk/government/publications/weight-management-services-national-mapping. Date accessed: 6.5.20.
20. Welbourn R, Hopkins J, Dixon JB, Finer N, Hughes C, Viner R, et al. Commissioning guidance for weight assessment and management in adults and children with severe complex obesity. Obes Rev. 2018;19(1):14-27.
21. National Institute for Health and Care Excellence. Weight management: lifestyle services for overweight or obese adults. London; 2014. Available from: https://www.nice.org.uk/guidance/ph53. Date accessed: 6.5.20.
22. Public Health England, National Institute for Health and Care Excellence. A Guide to Delivering and Commissioning Tier 2 Adult Weight Management Services. London; 2017. Available from:

https://assets.publishing.service.gov.uk/government/uploads/system/uploads/attachment_data/file/737905/Tier2_adult_weight_management_services__guide.pdf. Date accessed: 6.5.20.
23. British Obesity & Metabolic Surgery Society, The Royal College of Surgeons of England. Commissioning guide: Weight assessment and management clinics (tier 3). 2017. Available from: https://www.bomss.org.uk/wp-content/uploads/2017/10/Revision-of-Commissioning-guide-Tier-3-clinics-04042017.pdf. Date accessed: 6.5.20.
24. Welbourn R, Dixon J, Barth JH, Finer N, Hughes CA, le Roux CW, et al. NICE-Accredited Commissioning Guidance for Weight Assessment and Management Clinics: a Model for a Specialist Multidisciplinary Team Approach for People with Severe Obesity. Obes Surg. 2016;26(3):649-59.
25. Ahmad A, Laverty AA, Aasheim E, Majeed A, Millett C, Saxena S. Eligibility for bariatric surgery among adults in England: analysis of a national cross-sectional survey. JRSM open. 2014;5(1):2042533313512479.
26. Booth HP, Khan O, Fildes A, Prevost AT, Reddy M, Charlton J, et al. Changing Epidemiology of Bariatric Surgery in the UK: Cohort Study Using Primary Care Electronic Health Records. Obes Surg. 2016;26(8):1900-5.
27. Gulliford MC, Booth HP, Reddy M, Charlton J, Fildes A, Prevost AT, et al. Effect of Contemporary Bariatric Surgical Procedures on Type 2 Diabetes Remission. A Population-Based Matched Cohort Study. Obes Surg. 2016;26(10):2308-15.
28. Douglas IJ, Bhaskaran K, Batterham RL, Smeeth L. Bariatric Surgery in the United Kingdom: A Cohort Study of Weight Loss and Clinical Outcomes in Routine Clinical Care. PLoS Med. 2015;12(12):e1001925.
29. Koppe U, Nitsch D, Mansfield KE, Mathur R, Bhaskaran K, Batterham RL, et al. Long-term effects of bariatric surgery on acute kidney injury: a propensity-matched cohort in the UK Clinical Practice Research Datalink. BMJ Open. 2018;8(5):e020371.
30. NHS Digital. Quality and Outcomes Framework. 2020. Available from: https://qof.digital.nhs.uk/. Date accessed: 6.5.20.
31. Primary Care Domain, NHS Digital. Quality and Outcomes Framework - Prevalence, Achievements and Exceptions Report, England, 2015-16. 2016. Available from: https://webarchive.nationalarchives.gov.uk/20180328130852tf_/http://content.digital.nhs.uk/catalogue/PUB22266/qof-1516-rep-v2.pdf/. Date accessed: 6.5.20.
32. NHS England. Appendix 9 Guidance for Clinical Commissioning Groups (CCGs): Service Specification Guidance for Obesity Surgery. 2016. Available from: https://www.england.nhs.uk/wp-content/uploads/2016/05/appndx-9-serv-spec-ccg-guid.pdf. Date accessed: 6.5.20.
33. British Obesity & Metabolic Surgery Society, The Royal College of Surgeons. Patient access to bariatric surgery. 2017. Available from: https://www.rcseng.ac.uk/news-and-events/media-centre/press-releases/ccgs-restrict-bariatric-surgery/. Date accessed: 6.5.20.
34. Petersen I, Welch CA, Nazareth I, Walters K, Marston L, Morris RW, et al. Health indicator recording in UK primary care electronic health records: key implications for handling missing data. Clin Epidemiol. 2019;11:157-67.
35. Bhaskaran K, Forbes HJ, Douglas I, Leon DA, Smeeth L. Representativeness and optimal use of body mass index (BMI) in the UK Clinical Practice Research Datalink (CPRD). BMJ Open. 2013;3(9):e003389.
36. Booth HP, Prevost AT, Gulliford MC. Epidemiology of clinical body mass index recording in an obese population in primary care: a cohort study. J Public Health (Oxf). 2013;35(1):67-74.

List of Appendices
