# Supplementary material for: Access to publicly funded weight management services in England using routine data from primary and secondary care (2007–2020): An observational cohort study
Source: PLoS Med. 2023 Sep 28;20(9):e1004282. doi: 10.1371/journal.pmed.1004282 (PMC10538857; doi:10.1371/journal.pmed.1004282)
Supplement: S4 Appendix — NHS, National Health Service. (DOCX) [file pmed.1004282.s005.docx]

**S4 Appendix: National Health Service (NHS) bariatric surgery centres in England^a^, by Strategic Health Authority region^b^**

**North East (n=5)**

Darlington Memorial Hospital, County Durham

North Tyneside General Hospital, North Shields

University Hospital of North Tees, Stockton on Tees

The James Cook University Hospital, Middlesbrough

Sunderland Royal Hospital, Sunderland

**North West (n=3)**

Aintree University Hospital, Liverpool

Countess of Chester Hospital, Chester

Salford Royal, Salford

**Yorkshire and the Humber (n=7)**

Bradford Royal Infirmary, Bradford

Doncaster Royal Infirmary, Doncaster

Huddersfield Royal Infirmary, Huddersfield

Hull Royal Infirmary, Hull

St James’s University Hospital, Leeds

Northern General Hospital, Sheffield

York Hospital, York

**East Midlands (n=2)**

Royal Derby Hospital, Derby

Leicester Royal Infirmary, Leicester

**West Midlands (n=5)**

Heartlands Hospital, Birmingham

University Hospital Coventry, Coventry

Royal Shrewsbury Hospital, Shrewsbury

Manor Hospital, Walsall

Royal Stoke University Hospital, Stoke on Trent

**East of England (n=1)**

Luton and Dunstable Hospital, Luton

**South West (n=6)**

Royal Bournemouth General Hospital, Bournemouth

Gloucestershire Royal Hospital, Gloucestershire

Southmead Hospital, Bristol

Derriford Hospital, Plymouth

Musgrove Park Hospital, Taunton

Royal Cornwall Hospital, Truro

**South Central (n=3)**

Royal Berkshire hospital, Reading

Churchill Hospital, Oxford

Queen Alexandra Hospital, Portsmouth

**London (n=8)**

Chelsea and Westminster Hospital London

Guy’s and St Thomas’ Hospital London

Homerton University Hospital

King’s College Hospital, Denmark Hill

St Georges Hospital, Tooting

St Mary’s London

University College Hospital London

Whittington Hospital London

**South East Coast (n=3)**

Prince Royal University Hospital, Kent

St Richard’s Hospital, Chichester

St Peter’s Hospital, Chertsey, Surrey

**^a^List of NHS bariatric surgery centres provided by the British Obesity and Metabolic Surgery Society.**

**^b^Bariatic surgery centres grouped by Strategic Health Authority as per:** [**https://www.legislation.gov.uk/uksi/2006/1408/schedules/made**](https://www.legislation.gov.uk/uksi/2006/1408/schedules/made) **[Accessed 2023 July 29]**
