# Supplementary material for: Access to publicly funded weight management services in England using routine data from primary and secondary care (2007–2020): An observational cohort study
Source: PLoS Med. 2023 Sep 28;20(9):e1004282. doi: 10.1371/journal.pmed.1004282 (PMC10538857; doi:10.1371/journal.pmed.1004282)
Supplement: S1 Table — B&A, Black and Asian ethnic groups; BMI, body mass index; CPRD, Clinical Practice Research Datalink. aPercentage of the total variable. bMedical codes indicating diagnosis with overweight or obesity where BMI category not specified. cPercentage of “All” (n = 1,811,587). (DOCX) [file pmed.1004282.s008.docx]

**S1 Table: Characteristics of adults with recorded overweight and obesity in England in CPRD GOLD, by Body Mass Index group (all years 2007-2020)**

|  | **Body Mass Index (kg/m^2^) Group at diagnosis with overweight or obesity** | | | | | | |
| --- | --- | --- | --- | --- | --- | --- | --- |
|  | **23.0-24.9**  **(B&A only)** | **25.0-29.9** | **30.0-34.9** | **35.0-40.0** | **40.0 +** | **Medical codes^b^** | **All** |
|  | **n (%^a^)** | **n (%^a^)** | **n (%^a^)** | **n (%^a^)** | **n (%^a^)** | **n (%^a^)** | **n (%^c^)** |
| **Total** | 30,335 (1.67) | 1,041,808 (57.51) | 458,707 (25.32) | 167,802 (9.26) | 87,031 (4.80) | 25,904 (1.43) | 1,811,587 |
|  |  |  |  |  |  |  |  |
| ***Sex*** |  |  |  |  |  |  |  |
| Male | 12,573 (1.53) | 494,080 (60.10) | 215,526 (26.22) | 64,506 (7.85) | 26,370 (3.21) | 9,067 (1.10) | 822,122 (45.38) |
| Female | 17,762 (1.80) | 547,711 (55.36) | 243,171 (24.58) | 103,294 (10.44) | 60,659 (6.13) | 16,835 (1.70) | 989,432 (54.62) |
| Indeterminate/Unknown | 0 (0.00) | 17 (51.52) | 10 (30.30) | 2 (6.06) | 2 (6.06) | 2 (6.60) | 33 (0.00) |
|  |  |  |  |  |  |  |  |
| ***Age group at diagnosis with overweight or obesity*** |  |  |  |  |  |  |  |
| 18-24 | 2,878 (2.57) | 65,549 (58.56) | 24,951 (22.29) | 10,756 (9.61) | 6,318 (5.64) | 1,481 (1.32) | 111,933 (6.18) |
| 25-34 | 7,655 (3.56) | 121,378 (56.48) | 49,056 (22.83) | 20,896 (9.72) | 12,964 (6.03) | 2,948 (1.37) | 214,897 (11.86) |
| 35-44 | 7,507 (2.45) | 166,424 (54.36) | 77,025 (25.16) | 31,301 (10.22) | 18,618 (6.08) | 5,251 (1.72) | 306,126 (16.90) |
| 45-54 | 5,421 (1.49) | 196,704 (54.03) | 97,548 (26.79) | 37,615 (10.33) | 20,614 (5.66) | 6,182 (1.70) | 364,084 (20.10) |
| 55-64 | 3,287 (0.94) | 194,962 (55.81) | 95,064 (27.21) | 34,058 (9.75) | 16,509 (4.73) | 5,460 (1.56) | 349,340 (19.28) |
| 65-74 | 2,298 (0.83) | 168,352 (60.75) | 72,169 (26.04) | 22,344 (8.06) | 8,773 (3.17) | 3,190 (1.15) | 277,126 (15.30) |
| 75+ | 1,289 (0.69) | 128,439 (68.29) | 42,894 (22.81) | 10,832 (5.76) | 3,235 (1.72) | 1,392 (0.74) | 188,081 (10.38) |
|  |  |  |  |  |  |  |  |
| ***Ethnic group*** |  |  |  |  |  |  |  |
| White | 0 (0.00) | 794,917 (58.36) | 349,762 (25.68) | 129,426 (9.50) | 67,914 (4.99) | 20,008 (1.47) | 1,362,027 (75.18) |
| Asian | 19,363 (26.55) | 33,907 (46.50) | 13,688 (18.77) | 3,747 (5.14) | 1,435 (1.97) | 780 (1.07) | 72,920 (4.03) |
| Black | 7,865 (17.73) | 18,154 (40.92) | 11,146 (25.13) | 4,357 (9.82) | 2,281 (5.14) | 558 (1.26) | 44,361 (2.45) |
| Mixed | 3,107 (24.57) | 5,254 (41.55) | 2,648 (20.94) | 926 (7.32) | 544 (4.30) | 166 (1.31) | 12,645 (0.70) |
| Other | 0 (0.00) | 12,942 (62.65) | 4,939 (23.91) | 1,652 (8.00) | 735 (3.56) | 391 (1.89) | 20,659 (1.14) |
| Unknown | 0 (0.00) | 176,634 (59.08) | 76,524 (25.60) | 27,694 (9.26) | 14,122 (4.72) | 4,001 (1.34) | 298,975 (16.50) |
|  |  |  |  |  |  |  |  |
| ***Index of Multiple Deprivation*** |  |  |  |  |  |  |  |
| 1 (least deprived) | 3,638 (1.22) | 187,585 (62.99) | 70,843 (23.79) | 22,185 (7.45) | 9,604 (3.22) | 3,952 (1.33) | 297,807 (16.44) |
| 2 | 3,894 (1.32) | 176,505 (59.84) | 73,415 (24.89) | 24,816 (8.41) | 11,963 (4.06) | 4,367 (1.48) | 294,960 (16.28) |
| 3 | 4,433 (1.55) | 163,173 (57.20) | 73,140 (25.64) | 26,623 (9.33) | 13,441 (4,71) | 4,455 (1.56) | 285,265 (15.75) |
| 4 | 5,380 (2.03) | 144,091 (54.47) | 69,491 (26.27) | 27,036 (10.22) | 14,887 (5.63) | 3,645 (1.38) | 264,530 (14.60) |
| 5 (most deprived) | 6,695 (2.79) | 122,625 (51.13) | 63,368 (26.42) | 27,138 (11.32) | 16,264 (6.78) | 3,739 (1.56) | 239,829 (13.24) |
| *Missing* | 6,295 (1.47) | 247,829 (57.74) | 108,450 (25.27) | 40,004 (9.32) | 20,872 (4.86) | 5,746 (1.34) | 429,196 (23.69) |
|  |  |  |  |  |  |  |  |
| ***Smoking status*** |  |  |  |  |  |  |  |
| Current smoker | 4,192 (1.24) | 196,499 (57.98) | 85,217 (25.15) | 31,305 (9.24) | 16,290 (4.81) | 5,392 (1.59) | 338,895 (18.71) |
| Ex-smoker | 2,938 (0.63) | 267,092 (57.28) | 125,241 (26.86) | 43,926 (9.42) | 21,111 (4.53) | 5,955 (1.28) | 466,263 (25.74) |
| Never/non-smoker | 18,803 (2.27) | 480,734 (58.05) | 203,973 (24.63) | 74,958 (9.05) | 39,435 (4.76) | 10,186 (1.23) | 828,089 (45.71) |
| *Missing* | 4,402 (2.47) | 97,483 (54.66) | 44,276 (24.83) | 17,613 (9.88) | 10,195 (5.72) | 4,371 (2.45) | 178,340 (9.84) |
|  |  |  |  |  |  |  |  |
| ***Presence of co-morbidities*** |  |  |  |  |  |  |  |
| Type 2 diabetes | 4,124 (1.54) | 110,594 (41.19) | 83,478 (31.09) | 40,381 (15.04) | 25,541 (9.51) | 4,404 (1.64) | 268,522 (14.82) |
| Hypertension | 4,476 (0.90) | 254,620 (51.47) | 142,069 (28.72) | 56,311 (11.38) | 30,149 (6.09) | 7,069 (1.43) | 494,694 (27.31) |
| Coronary Heart Disease | 2,200 (0.99) | 126,260 (56.61) | 61,136 (27.41) | 21,158 (9.49) | 9,528 (4.27) | 2,760 (1.24) | 223,042 (12.31) |
| Obstructive Sleep Apnoea | 119 (0.41) | 7,999 (27.74) | 8,442 (29.27) | 5,613 (19.46) | 5,538 (19.20) | 1,128 (3.91) | 28,839 (1.59) |
| Asthma | 3,842 (1.38) | 147,765 (52.93) | 72,522 (25.98) | 30,970 (11.09) | 19,214 (6.88) | 4,880 (1.75) | 279,193 (15.41) |
| Chronic musculoskeletal condition | 2,671 (0.69) | 205,047 (52.74) | 109,541 (28.18) | 42,473 (10.93) | 22,447 (5.77) | 6,578 (1.69) | 388,757 (21.46) |
| Gastro-oesophageal reflux disease | 7,976 (1.61) | 280,047 (56.40) | 130,300 (26.24) | 46,763 (9.42) | 23,437 (4.72) | 7,972 (1.61) | 496,495 (27.41) |
| Liver disease | 473 (1.24) | 18,660 (49.12) | 10,771 (28.35) | 4,635 (12.20) | 2,729 (7.18) | 724 (1.91) | 37,992 (2.10) |
| Polycystic ovarian syndrome | 656 (2.43) | 10,393 (38.47) | 6,952 (25.74) | 4,404 (16.30) | 3,716 (13.76) | 892 (3.30) | 27,013 (1.49) |
| Fertility problems | 1,448 (3.39) | 23,444 (54.93) | 10,141 (23.76) | 4,291 (10.05) | 2,504 (5.87) | 849 (1.99) | 42,677 (2.36) |
| Depression | 4,774 (1.01) | 250,384 (53.17) | 122,994 (26.12) | 51,979 (11.04) | 31,487 (6.69) | 9,331 (1.98) | 470,949 (26.00) |
| Anxiety | 3,895 (1.12) | 193,758 (55.68) | 88,527 (25.44) | 35,115 (10.09) | 20,156 (5.79) | 6,504 (1.87) | 347,955 (19.21) |
| Idiopathic Intracranial Hypertension | 10 (0.59) | 385 (22.70) | 434 (25.59) | 380 (22.41) | 413 (24.35) | 74 (4.36) | 1,696 (0.09) |
|  |  |  |  |  |  |  |  |
| ***Strategic Health Authority of GP practice*** |  |  |  |  |  |  |  |
| North East | 399 (1.00) | 22,856 (57.37) | 10,361 (26.01) | 3,810 (9.56) | 1,972 (4.95) | 439 (1.10) | 39,837 (2.20) |
| North West | 2,544 (0.91) | 164,153 (58.52) | 70,714 (25.21) | 26,115 (9.31) | 13,298 (4.74) | 3,695 (1.32) | 280,519 (15.48) |
| Yorkshire & the Humber | 251 (0.39) | 36,903 (57.54) | 16,646 (25.95) | 6,253 (9.75) | 3,241 (5.05) | 845 (1.32) | 64,139 (3.54) |
| East Midlands | 386 (0.61) | 34,704 (54.86) | 17,097 (27.03) | 6,640 (10.50) | 3,671 (5.80) | 762 (1.20) | 63,260 (3.49) |
| West Midlands | 4,982 (2.00) | 140,407 (56.36) | 63,577 (25.52) | 23,370 (9.38) | 12,444 (4.99) | 4,363 (1.75) | 249,143 (13.75) |
| East of England | 2,705 (1.57) | 100,174 (58.18) | 43,505 (25.27) | 15,319 (8.90) | 7,723 (4.49) | 2,751 (1.60) | 172,177 (9.50) |
| South West | 1,235 (0.61) | 114,633 (56.78) | 52,769 (26.14) | 20,056 (9.93) | 10,568 (5.23) | 2,632 (1.30) | 201,893 (11.14) |
| South Central | 3,137 (1.29) | 141,549 (58.21) | 61,357 (25.23) | 22,494 (9.25) | 11,463 (4.71) | 3,158 (1.30) | 243,158 (13.42) |
| London | 12,394 (5.52) | 125,841 (56.02) | 54,060 (24.06) | 18,886 (8.41) | 9,727 (4.33) | 3,741 (1.67) | 224,649 (12.40) |
| South East Coast | 2,302 (0.84) | 160,588 (58.86) | 68,621 (25.15) | 24,859 (9.11) | 12,924 (4.74) | 3,518 (1.29) | 272,812 (15.06) |
|  |  |  |  |  |  |  |  |
| ***Rural-urban classification of GP practice*** |  |  |  |  |  |  |  |
| Rural | 510 (0.28) | 107,400 (58.24) | 47,666 (25.85) | 16,783 (9.10) | 8,276 (4.49) | 3,766 (2.04) | 184,401 (10.18) |
| Urban | 23,965 (1.94) | 707,814 (57.34) | 311,729 (25.25) | 114,333 (9.26) | 59,656 (4.83) | 16,891 (1.37) | 1,234,388 (68.14) |
| *Missing* | 5,860 (1.49) | 226,594 (57.69) | 99,312 (25.28) | 36,686 (9.34) | 19,099 (4.86) | 5,247 (1.34) | 392,798 (21.68) |
|  |  |  |  |  |  |  |  |

B&A=Black and Asian ethnic groups. ^a^Percentage of the total variable. ^b^Medical codes indicating diagnosis with overweight or obesity where BMI category not specified. ^c^Percentage of ‘All’ (n=1,811,587)
