# Supplementary material for: Access to publicly funded weight management services in England using routine data from primary and secondary care (2007–2020): An observational cohort study
Source: PLoS Med. 2023 Sep 28;20(9):e1004282. doi: 10.1371/journal.pmed.1004282 (PMC10538857; doi:10.1371/journal.pmed.1004282)
Supplement: S2 Table — Text in italics indicates the reference group for the Poisson regression model. B&A, Black and Asian ethnic groups; BMI, body mass index; CI, confidence interval; CPRD, Clinical Practice Research Datalink; IMD, Index of Multiple Deprivation; NHS, National Health Service; RR, rate ratio; WM, weight management. aPatients without “research quality” data excluded (N = 18,935). bVariables included in adjusted model include sex, age group at diagnosis with overweight/obesity, strategic Health Authority of GP practice, rural–urban classification of GP practice, year of diagnosis with overweight/obesity, BMI category at diagnosis with overweight/obesity, ethnic group, IMD, smoking status, total number of comorbidities. cNumber of individuals included in the crude analysis. dNumber of individuals with complete data for all variables included in the adjusted analysis. eIndeterminate and unknown sex excluded due to small numbers (n = 18). (DOCX) [file pmed.1004282.s009.docx]

**S2 Table: Rate Ratios for NHS weight management (WM) referral within adults eligible for WM referral in England with at least two years of follow-up data in CPRD GOLD (2007-2020)^a^ (MODEL B)**

|  | **Eligible for a WM referral (N=)** | **Received a WM referral (N=) (% of those eligible)** | **Crude RR**  **(95% CI)** | **P-value** | **Adjusted RR**  **(95% CI)^b^** | **P-value** |
| --- | --- | --- | --- | --- | --- | --- |
|  |  |  |  |  |  |  |
| **Total** | 1,751,385 | 53,270 (3.04) | 1,751,385^c^ |  | 1,228,024^d^ |  |
|  |  |  |  |  |  |  |
| **Sex^e^** |  |  |  |  |  |  |
| Male | 795,602 | 17,896 (2.25) | 0.61 (0.60,0.62) | <0.001 | 0.65 (0.64,0.67) | <0.001 |
| *Female* | 955,783 | 35,374 (3.70) | 1.0 |  | 1.0 |  |
|  |  |  |  |  |  |  |
| **Age group at diagnosis with overweight or obesity** |  |  |  |  |  |  |
| 18-24 | 107,515 | 2,464 (2.29) | 0.54 (0.52,0.57) | <0.001 | 0.55 (0.52,0.58) | <0.001 |
| 25-34 | 206,868 | 5,342 (2.58) | 0.61 (0.59,0.63) | <0.001 | 0.64 (0.62,0.66) | <0.001 |
| 35-44 | 295,335 | 12,174 (4.12) | 0.98 (0.96,1.00) | 0.072 | 1.00 (0.97,1.03) | 0.870 |
| *45-54* | 351,288 | 14,796 (4.21) | 1.0 |  | 1.0 |  |
| 55-64 | 338,340 | 11,998 (3.55) | 0.84 (0.82,0.86) | <0.001 | 0.87 (0.85,0.89) | <0.001 |
| 65-74 | 269,041 | 5,695 (2.12) | 0.50 (0.49,0.52) | <0.001 | 0.54 (0.52,0.56) | <0.001 |
| 75+ | 182,998 | 801 (0.44) | 0.10 (0.10,0.11) | <0.001 | 0.13 (0.12,0.14) | <0.001 |
|  |  |  |  |  |  |  |
| **Strategic Health Authority of GP practice** |  |  |  |  |  |  |
| North East | 39,114 | 1,397 (3.57) | 1.24 (1.17,1.31) | <0.001 | 1.61 (1.51,1.71) | <0.001 |
| *North West* | 271,163 | 7,826 (2.89) | 1.0 |  | 1.0 |  |
| Yorkshire & the Humber | 63,586 | 751 (1.18) | 0.41 (0.38,0.44) | <0.001 | 0.60 (0.56,0.65) | <0.001 |
| East Midlands | 62,955 | 1,825 (2.90) | 1.00 (0.96,1.06) | 0.863 | 1.61 (1.52,1.70) | <0.001 |
| West Midlands | 237,361 | 12,828 (5.40) | 1.87 (1.82,1.92) | <0.001 | 2.18 (2.11,2.25) | <0.001 |
| East of England | 169,466 | 1,762 (1.04) | 0.36 (0.34,0.38) | <0.001 | 0.43 (0.41,0.46) | <0.001 |
| South West | 196,943 | 4,613 (2.34) | 0.81 (0.78,0.84) | <0.001 | 0.98 (0.94,1.02) | 0.251 |
| South Central | 237,355 | 6,566 (2.77) | 0.96 (0.93,0.99) | 0.010 | 1.22 (1.17,1.27) | <0.001 |
| London | 214,783 | 5,836 (2.72) | 0.94 (0.91,0.97) | <0.001 | 1.10 (1.06,1.15) | <0.001 |
| South East Coast | 258,659 | 9,866 (3.81) | 1.32 (1.28,1.36) | <0.001 | 1.64 (1.58,1.69) | <0.001 |
|  |  |  |  |  |  |  |
| **Rural-urban classification of GP practice** |  |  |  |  |  |  |
| *Urban* | 1,196,702 | 35,217 (2.94) | 1.0 |  | 1.0 |  |
| Rural | 180,091 | 4,376 (2.43) | 0.83 (0.80,0.85) | <0.001 | 0.88 (0.85,0.91) | <0.001 |
| Data missing/not recorded | *374,592* | *13,677 (3.65)* | - |  |  |  |
|  |  |  |  |  |  |  |
| **Year of diagnosis with overweight/obesity** |  |  |  |  |  |  |
| *2007* | 531,157 | 16,637 (3.13) | 1.0 |  | 1.0 |  |
| 2008 | 271,624 | 7,842 (2.89) | 0.92 (0.90,0.95) | <0.001 | 0.99 (0.96,1.02) | 0.333 |
| 2009 | 192,610 | 5,447 (2.83) | 0.90 (0.88,0.93) | <0.001 | 0.96 (0.93,1.00) | 0.030 |
| 2010 | 154,843 | 3,807 (2.46) | 0.78 (0.76,0.81) | <0.001 | 0.88 (0.85,0.92) | <0.001 |
| 2011 | 131,933 | 3,352 (2.54) | 0.81 (0.78,0.84) | <0.001 | 0.93 (0.89,0.97) | 0.002 |
| 2012 | 123,252 | 3,380 (2.74) | 0.88 (0.84,0.91) | <0.001 | 1.02 (0.98,1.07) | 0.292 |
| 2013 | 108,539 | 3,696 (3.41) | 1.09 (1.05,1.13) | <0.001 | 1.34 (1.28,1.39) | <0.001 |
| 2014 | 83,549 | 3,088 (3.70) | 1.18 (1.14,1.23) | <0.001 | 1.39 (1.33,1.45) | <0.001 |
| 2015 | 64,674 | 2,455 (3.80) | 1.21 (1.16,1.26) | <0.001 | 1.32 (1.25,1.39) | <0.001 |
| 2016 | 43,212 | 1,664 (3.85) | 1.23 (1.17,1.29) | <0.001 | 1.43 (1.35,1.52) | <0.001 |
| 2017 | 33,218 | 1,388 (4.18) | 1.33 (1.26,1.41) | <0.001 | 1.32 (1.23,1.41) | <0.001 |
| 2018 | 12,774 | 514 (4.02) | 1.28 (1.18,1.40) | <0.001 | 1.42 (1.27,1.58) | <0.001 |
|  |  |  |  |  |  |  |
| **BMI category (kg/m^2^) at diagnosis with overweight/obesity** |  |  |  |  |  |  |
| 23.0-24.9 (B&A only) | 29,158 | 178 (0.61) | 0.45 (0.39,0.52) | <0.001 | 0.36 (0.30,0.43) | <0.001 |
| *25.0-29.9* | 1,006,478 | 13,529 (1.34) | 1.0 |  | 1.0 |  |
| 30.0-34.9 | 444,510 | 17,240 (3.88) | 2.89 (2.82,2.95) | <0.001 | 2.79 (2.72,2.86) | <0.001 |
| 35.0-40.0 | 162,595 | 10,025 (6.17) | 4.59 (4.47,4.70) | <0.001 | 4.09 (3.97,4.22) | <0.001 |
| 40.0 + | 83,901 | 8,436 (10.05) | 7.48 (7.29,7.68) | <0.001 | 6.04 (5.85,6.23) | <0.001 |
| Medical codes | 24,743 | 3,862 (15.61) | 11.61 (11.23,12.01) | <0.001 | 9.06 (8.70,9.43) | <0.001 |
|  |  |  |  |  |  |  |
| **Ethnic group** |  |  |  |  |  |  |
| *White* | 1,322,847 | 42,347 (3.20) | 1.0 |  | 1.0 |  |
| Asian | 70,014 | 1,723 (2.46) | 0.77 (0.73,0.81) | <0.001 | 0.94 (0.89,1.00) | 0.062 |
| Black | 42,712 | 1,520 (3.56) | 1.11 (1.06,1.17) | <0.001 | 1.14 (1.07,1.21) | <0.001 |
| Mixed | 12,009 | 355 (2.96) | 0.92 (0.83,1.02) | 0.129 | 0.98 (0.86,1.11) | 0.751 |
| Other | 19,647 | 576 (2.93) | 0.92 (0.84,0.99) | 0.033 | 0.90 (0.82,0.99) | 0.031 |
| Unknown | 284,156 | 6,749 (2.38) | 0.74 (0.72,0.76) | <0.001 | 0.69 (0.66,0.72) | <0.001 |
| **Index of Multiple Deprivation** |  |  |  |  |  |  |
| *1 (least deprived)* | 290,170 | 7,017 (2.42) | 1.0 |  | 1.0 |  |
| 2 | 288,664 | 8,374 (2.90) | 1.20 (1.16,1.24) | <0.001 | 1.13 (1.09,1.16) | <0.001 |
| 3 | 279,202 | 7,219 (2.59) | 1.07 (1.04,1.10) | <0.001 | 0.99 (0.96,1.02) | 0.493 |
| 4 | 259,566 | 7,875 (3.03) | 1.25 (1.22,1.30) | <0.001 | 1.09 (1.06,1.13) | <0.001 |
| 5 (most deprived) | 235,220 | 8,696 (3.70) | 1.53 (1.48,1.58) | <0.001 | 1.16 (1.12,1.19) | <0.001 |
| Data missing/not recorded | 398,563 | 14,089 (3.53) | - |  | - |  |
|  |  |  |  |  |  |  |
| **Smoking status** |  |  |  |  |  |  |
| *Non-smoker* | 802,920 | 26,526 (3.30) | 1.0 |  | 1.0 |  |
| Current smoker | 330,257 | 11,084 (3.36) | 1.02 (0.99,1.04) | 0.156 | 0.94 (0.92,0.97) | <0.001 |
| Ex-smoker | 453,803 | 13,763 (3.03) | 0.92 (0.90,0.94) | <0.001 | 1.00 (0.98,1.03) | 0.860 |
| Data missing/not recorded | 164,405 | 1,897 (1.15) | - |  | - |  |
|  |  |  |  |  |  |  |
|  |  |  |  |  |  |  |
| **Total co-morbidities** |  |  |  |  |  |  |
| *0* | 413,602 | 10,714 (2.59) | 1.0 |  | 1.0 |  |
| 1 | 477,600 | 13,195 (2.76) | 1.07 (1.04,1.09) | <0.001 | 0.98 (0.95,1.01) | 0.115 |
| 2 | 387,124 | 11,672 (3.02) | 1.16 (1.13,1.19) | <0.001 | 1.02 (0.99,1.06) | 0.174 |
| 3 | 248,112 | 8,241 (3.32) | 1.28 (1.25,1.32) | <0.001 | 1.09 (1.05,1.13) | <0.001 |
| 4 | 132,761 | 5,091 (3.83) | 1.48 (1.43,1.53) | <0.001 | 1.19 (1.14,1.24) | <0.001 |
| 5 | 59,770 | 2,590 (4.33) | 1.67 (1.60,1.74) | <0.001 | 1.27 (1.21,1.33) | <0.001 |
| 6+ | 32,416 | 1,767 (5.45) | 2.10 (2.00,2.21) | <0.001 | 1.39 (1.32,1.47) | <0.001 |

Text in *italics* indicates the reference group for the Poisson regression model. B&A=Black and Asian ethnic groups, BMI=Body Mass Index, CI=Confidence Interval, RR=Rate Ratio. ^a^Patients without ‘research quality’ data excluded (N=18,935). ^b^Variables included in adjusted model include sex, age group at diagnosis with overweight/obesity, strategic Health Authority of GP practice, rural-urban classification of GP practice, year of diagnosis with overweight/obesity, BMI category at diagnosis with overweight/obesity, ethnic group, Index of Multiple Deprivation, smoking status, total number of co-morbidities. ^c^Number of individuals included in the crude analysis. ^d^Number of individuals with complete data for all variables included in the adjusted analysis. ^e^Indeterminate and unknown sex excluded due to small numbers (n=18).
