# Supplementary material for: Access to publicly funded weight management services in England using routine data from primary and secondary care (2007–2020): An observational cohort study
Source: PLoS Med. 2023 Sep 28;20(9):e1004282. doi: 10.1371/journal.pmed.1004282 (PMC10538857; doi:10.1371/journal.pmed.1004282)
Supplement: S3 Table — Text in italics indicates the reference group for the Poisson regression model. B&A, Black and Asian ethnic groups; BMI, body mass index; CI, confidence interval; CPRD, Clinical Practice Research Datalink; GP, general practitioner; IMD, Index of Multiple Deprivation; RR, rate ratio. aA total of 21 individuals with bariatric surgery before their severe and complex obesity index date excluded. bVariables included in adjusted model include sex, age group at diagnosis with severe and complex obesity, strategic health authority of GP practice, rural–urban classification of GP practice, year of diagnosis with severe and complex obesity, BMI category at diagnosis with severe and complex obesity, ethnic group, IMD, smoking status, total number of comorbidities. cNumber of individuals included in the crude analysis. dNumber of individuals with complete data for all variables included in the adjusted analysis. (DOCX) [file pmed.1004282.s010.docx]

**S3 Table: Rate Ratios for provision of bariatric surgery within adults eligible for bariatric surgery in England with at least 5 years of follow-up data in CPRD GOLD (2007-2020).^a^ (MODEL B)**

|  | **Eligible for bariatric surgery (N=)** | **Underwent bariatric surgery (N=) (% of those eligible)** | **Crude RR**  **(95% CI)** | **P-value** | **Adjusted RR**  **(95% CI)^b^** | **P-value** |
| --- | --- | --- | --- | --- | --- | --- |
|  |  |  |  |  |  |  |
| **Total** | 297,332 | 3,604 (1.21) | 297,332^c^ |  | 280,316^d^ |  |
|  |  |  |  |  |  |  |
| **Sex** |  |  |  |  |  |  |
| Male | 125,147 | 852 (0.68) | 0.43 (0.39,0.46) | <0.001 | 0.78 (0.72,0.84) | <0.001 |
| *Female* | 172,185 | 2,752 (1.60) | 1.0 |  | 1.0 |  |
|  |  |  |  |  |  |  |
| **Age group at diagnosis with severe and complex obesity** |  |  |  |  |  |  |
| 18-24 | 11,173 | 237 (2.12) | 1.21 (1.05,1.39) | 0.008 | 1.46 (1.26,1.70) | <0.001 |
| 25-34 | 26,016 | 696 (2.68) | 1.52 (1.39,1.67) | <0.001 | 1.63 (1.48,1.81) | <0.001 |
| 35-44 | 43,386 | 1,174 (2.71) | 1.54 (1.42,1.67) | <0.001 | 1.45 (1.33,1.57) | <0.001 |
| *45-54* | 60,640 | 1,065 (1.76) | 1.0 |  | 1.0 |  |
| 55-64 | 66,549 | 384 (0.58) | 0.33 (0.29,0.37) | <0.001 | 0.35 (0.31,0.40) | <0.001 |
| 65-74 | 54,893 | 47 (0.09) | 0.05 (0.04,0.07) | <0.001 | 0.06 (0.05,0.09) | <0.001 |
| 75+ | 34,675 | 1 (0.00) | 0.00 (0.00,0.01) | <0.001 | 0.00 (0.00,0.02), | <0.001 |
|  |  |  |  |  |  |  |
| **Strategic Health Authority of GP practice** |  |  |  |  |  |  |
| North East | 7,951 | 188 (2.36) | 3.46 (2.91,4.13) | <0.001 | 3.05 (2.56,3.63) | <0.001 |
| *North West* | 52,436 | 358 (0.68) | 1.0 |  | 1.0 |  |
| Yorkshire & the Humber | 12,627 | 167 (1.32) | 1.94 (1.61,2.32) | <0.001 | 1.92 (1.60,2.30) | <0.001 |
| East Midlands | 9,836 | 102 (1.04) | 1.52 (1.22,1.89) | <0.001 | 1.36 (1.08,1.71) | 0.009 |
| West Midlands | 36,765 | 391 (1.06) | 1.56 (1.35,1.80) | <0.001 | 1.51 (1.31,1.75) | <0.001 |
| East of England | 29,996 | 257 (0.86) | 1.25 (1.07,1.47) | 0.005 | 1.31 (1.11,1.55) | 0.001 |
| South West | 40,610 | 483 (1.19) | 1.74 (1.52,2.00) | <0.001 | 1.76 (1.53,2.02) | <0.001 |
| South Central | 36,366 | 423 (1.16) | 1.70 (1.48,1.96) | <0.001 | 1.74 (1.51,2.01) | <0.001 |
| London | 31,359 | 673 (2.15) | 3.14 (2.77,3.57) | <0.001 | 3.55 (3.11,4.05) | <0.001 |
| South East Coast | 39,386 | 562 (1.43) | 2.09 (1.83,2.38) | <0.001 | 2.28 (2.00,2.61) | <0.001 |
|  |  |  |  |  |  |  |
| **Rural-urban classification of GP practice** |  |  |  |  |  |  |
| *Urban* | 258,135 | 3,197 (1.24) | 1.0 |  | 1.0 |  |
| Rural | 39,197 | 407 (1.04) | 0.84 (0.76,0.93) | 0.001 | 1.01 (0.90,1.12) | 0.891 |
| Data missing/not recorded |  | - | - |  | - |  |
|  |  |  |  |  |  |  |
| **Year of diagnosis with severe and complex obesity** |  |  |  |  |  |  |
| *2007* | 99,441 | 1,658 (1.67) | 1.0 |  | 1.0 |  |
| 2008 | 42,133 | 666 (1.58) | 0.95 (0.87,1.04) | 0.241 | 0.89 (0.81,0.98) | 0.013 |
| 2009 | 32,030 | 387 (1.21) | 0.72 (0.65,0.81) | <0.001 | 0.73 (0.65,0.81) | <0.001 |
| 2010 | 28,358 | 255 (0.90) | 0.54 (0.47,0.61) | <0.001 | 0.62 (0.51,0.71) | <0.001 |
| 2011 | 25,679 | 226 (0.88) | 0.53 (0.46,0.61) | <0.001 | 0.60 (0.52,0.70) | <0.001 |
| 2012 | 23,958 | 165 (0.69) | 0.41 (0.35,0.48) | <0.001 | 0.54 (0.45,0.63) | <0.001 |
| 2013 | 21,740 | 123 (0.57) | 0.34 (0.28,0.41) | <0.001 | 0.43 (0.36,0.53) | <0.001 |
| 2014 | 17,304 | 101 (0.58) | 0.35 (0.29,0.43) | <0.001 | 0.43 (0.34,0.53) | <0.001 |
| 2015 | 6,689 | 23 (0.34) | 0.21 (0.14,0.31) | <0.001 | 0.21 (0.12,0.35) | <0.001 |
|  |  |  |  |  |  |  |
| **BMI category (kg/m^2^) at diagnosis with severe and complex obesity** |  |  |  |  |  |  |
| 27.5-29.9 in B&A groups with T2DM diagnosed no more than 10 years prior to first eligible BMI measurement | 36,429 | 7 (0.02) | 0.04 (0.02,0.08) | <0.001 | 0.08 (0.04,0.16) | <0.001 |
| 30.0-34.9 with T2DM diagnosed no more than 10 years prior to first eligible BMI measurement | 52,669 | 44 (0.08) | 0.16 (0.12,0.21) | <0.001 | 0.25 (0.19,0.35) | <0.001 |
| *35.0-40.0 with weight related co-morbidity* | 137,378 | 730 (0.53) | 1.0 |  | 1.0 |  |
| 40.0 + | 70,713 | 2,817 (3.98) | 7.50 (6.91,8.13) | <0.001 | 6.04 (5.54,6.58) | <0.001 |
| Diagnosis of severe and complex obesity as per medical codes | 143 | 6 (4.20) | 7.90 (3.60,17.34) | <0.001 | 13.24 (5.96,29.39) | <0.001 |
| **Ethnic group** |  |  |  |  |  |  |
| *White* | 258,969 | 3,268 (1.26) | 1.0 |  | 1.0 |  |
| Asian | 9,113 | 89 (0.98) | 0.77 (0.63,0.95) | 0.017 | 0.92 (0.73,1.14) | 0.439 |
| Black | 7,263 | 117 (1.61) | 1.28 (1.06,1.53) | 0.009 | 0.86 (0.71,1.05) | 0.141 |
| Mixed | 1,430 | 42 (2.94) | 2.33 (1.72,3.14) | <0.001 | 1.40 (1.01,1.94) | 0.042 |
| Other | 2,713 | 61 (2.25) | 1.78 (1.39,2.29) | <0.001 | 1.25 (0.95,1.63) | 0.106 |
| Unknown | 17,844 | 27 (0.15) | 0.12 (0.08,0.18) | <0.001 | 0.14 (0.09,0.21) | <0.001 |
|  |  |  |  |  |  |  |
| **Index of Multiple Deprivation** |  |  |  |  |  |  |
| *1 (least deprived)* | 49,899 | 431 (0.86) | 1.0 |  | 1.0 |  |
| 2 | 58,437 | 626 (1.07) | 1.24 (1.10,1.40) | 0.001 | 1.09 (0.96,1.23) | 0.179 |
| 3 | 61,865 | 741 (1.20) | 1.39 (1.23,1.56) | <0.001 | 1.11 (0.98,1.25) | 0.090 |
| 4 | 62,890 | 870 (1.38) | 1.60 (1.43,1.80) | <0.001 | 1.04 (0.92,1.17) | 0.552 |
| 5 (most deprived) | 64,054 | 935 (1.46) | 1.69 (1.51,1.89) | <0.001 | 1.02 (0.90,1.15) | 0.807 |
| Data missing/not recorded | 187 | 1 (0.53) | - |  | - |  |
|  |  |  |  |  |  |  |
| **Smoking status** |  |  |  |  |  |  |
| *Non-smoker* | 131,749 | 1,580 (1.20) | 1.0 |  | 1.0 |  |
| Current smoker | 59,622 | 767 (1.29) | 1.07 (0.98,1.17) | 0.109 | 0.87 (0.80,0.95) | 0.002 |
| Ex-smoker | 89,118 | 961 (1.08) | 0.90 (0.83,0.97) | 0.009 | 1.23 (1.13,1.33) | <0.001 |
| Data missing/not recorded | 16,843 | 296 (1.76) | - |  | - |  |
|  |  |  |  |  |  |  |
| **Total co-morbidities** |  |  |  |  |  |  |
| *0* | 11,737 | 163 (1.39) | 1.0 |  | 1.0 |  |
| 1 | 59,402 | 476 (0.80) | 0.58 (0.48,0.69) | <0.001 | 1.87 (1.53,2.28) | <0.001 |
| 2 | 73,894 | 684 (0.93) | 0.67 (0.56,0.79) | <0.001 | 2.78 (2.29,3.38) | <0.001 |
| 3 | 63,963 | 710 (1.11) | 0.80 (0.67,0.95) | 0.009 | 3.94 (3.25,4.78) | <0.001 |
| 4 | 44,829 | 693 (1.55) | 1.11 (0.94,1.32) | 0.215 | 5.80 (4.77,7.04) | <0.001 |
| 5 | 25,395 | 469 (1.85) | 1.33 (1.11,1.59) | 0.002 | 7.36 (6.02,9.01) | <0.001 |
| 6+ | 18,112 | 409 (2.26) | 1.63 (1.36,1.95) | <0.001 | 8.79 (7.16,10.79) | <0.001 |
|  |  |  |  |  |  |  |

Text in *italics* indicates the reference group for the Poisson regression model. B&A=Black and Asian ethnic groups, BMI=Body Mass Index, CI=Confidence Interval, RR=Rate Ratio. ^a^21 individuals with bariatric surgery before their severe and complex obesity index date excluded. ^b^Variables included in adjusted model include sex, age group at diagnosis with severe and complex obesity, strategic health authority of GP practice, rural-urban classification of GP practice, year of diagnosis with severe and complex obesity, BMI category at diagnosis with severe and complex obesity, ethnic group, Index of Multiple Deprivation, smoking status, total number of co-morbidities. ^c^Number of individuals included in the crude analysis. ^d^Number of individuals with complete data for all variables included in the adjusted analysis.
