# Supplementary material for: Access to publicly funded weight management services in England using routine data from primary and secondary care (2007–2020): An observational cohort study
Source: PLoS Med. 2023 Sep 28;20(9):e1004282. doi: 10.1371/journal.pmed.1004282 (PMC10538857; doi:10.1371/journal.pmed.1004282)
Supplement: S4 Table — B&A, Black and Asian ethnic groups; BMI, body mass index; WM, weight management. aIncluded in adjusted model. bMedical codes indicating diagnosis with overweight or obesity where BMI category not specified. (DOCX) [file pmed.1004282.s011.docx]

**S4 Table: Characteristics of patients included in adjusted Poisson model versus those in full sample (weight management referral as outcome)**

|  | **Patients with overweight/obesity with ≥2 years follow-up data (% of total)** | **Patients with overweight/obesity with ≥2 years follow-up data and complete data for all variables^a^ (% of total)** |  |
| --- | --- | --- | --- |
|  |  |  |  |
| ***Total*** | 1,751,385 | 1,228,024 |  |
|  |  |  |  |
| ***Sex*** |  |  |  |
| Male | 795,602 (45.43) | 560,622 (45.65) |  |
| Female | 955,783 (54.57) | 667,402 (54.35) |  |
|  |  |  |  |
| ***Age group at diagnosis with overweight or obesity*** |  |  |  |
| 18-24 | 107,515 (6.14) | 73,175 (5.96) |  |
| 25-34 | 206,868 (11.81) | 135,464 (11.03) |  |
| 35-44 | 295,335 (16.86) | 202,396 (16.48) |  |
| 45-54 | 351,288 (20.06) | 247,675 (20.17) |  |
| 55-64 | 338,340 (19.32) | 243,464 (19.83) |  |
| 65-74 | 269,041 (15.36) | 195,704 (15.94) |  |
| 75+ | 182,998 (10.45) | 130,146 (10.60) |  |
|  |  |  |  |
| ***Strategic Health Authority of GP practice*** |  |  |  |
| North East | 39,114 (2.23) | 28,542 (2.32) |  |
| North West | 271,163 (15.48) | 209,384 (17.05) |  |
| Yorkshire & the Humber | 63,586 (3.63) | 49,210 (4.01) |  |
| East Midlands | 62,955 (3.59) | 33,525 (2.73) |  |
| West Midlands | 237,361 (13.55) | 152,020 (12.38) |  |
| East of England | 169,466 (9.68) | 126,067 (10.27) |  |
| South West | 196,943 (11.24) | 153,913 (12.53) |  |
| South Central | 237,355 (13.55) | 156,010 (12.70) |  |
| London | 214,783 (12.26) | 141,233 (11.50) |  |
| South East Coast | 258,659 (14.77) | 178,120 14.50) |  |
|  |  |  |  |
| ***Rural-urban classification of GP practice*** |  |  |  |
| Urban | 1,196,702 (68.33) | 1,071,979 (87.29) |  |
| Rural | 180,091 (10.28) | 156,045 (12.71) |  |
| *Data missing/not recorded* | *374,592 (21.39)* |  |  |
|  |  |  |  |
| ***Year of diagnosis with severe and complex obesity*** |  |  |  |
| 2007 | 531,157 (30.33) | 390,436 (31.79) |  |
| 2008 | 271,624 (15.51) | 198,422 (16.16) |  |
| 2009 | 192,610 (11.00) | 136,003 (11.07) |  |
| 2010 | 154,843 (8.84) | 107,835 (8.78) |  |
| 2011 | 131,933 (7.53) | 92,320 (7.52) |  |
| 2012 | 123,252 (7.04) | 86,192 (7.02) |  |
| 2013 | 108,539 (6.20) | 72,261 (5.88) |  |
| 2014 | 83,549 (4.77) | 52,662 (4.29) |  |
| 2015 | 64,674 (3.69) | 38,291 (3.12) |  |
| 2016 | 43,212 (2.47) | 26,173 (2.13) |  |
| 2017 | 33,218 (1.90) | 19,768 (1.61) |  |
| 2018 | 12,774 (0.73) | 7,661 (0.62) |  |
|  |  |  |  |
| ***BMI category (kg/m^2^) at diagnosis with severe and complex obesity*** |  |  |  |
| 23.0-24.9 (B&A only) | 29,158 (1.66) | 20,004 (1.63) |  |
| 25.0-29.9 | 1,006,478 (57.47) | 708,352 (57.68) |  |
| 30.0-34.9 | 444,510 (25.38) | 312,465 (25.44) |  |
| 35.0-40.0 | 162,595 (9.28) | 113,099 (9.21) |  |
| 40.0 + | 83,901 (4.79) | 57,710 (4.70) |  |
| Medical codes^b^ | 24,743 (1.41) | 16,394 (1.33) |  |
|  |  |  |  |
| ***Ethnic group*** |  |  |  |
| White | 1,322,847 (75.53) | 1,021,001 (83.14) |  |
| Asian | 70,014 (4.00) | 47,068 (3.83) |  |
| Black | 42,712 (2.44) | 29,855 (2.43) |  |
| Mixed | 12,009 (0.69) | 8,331 (0.68) |  |
| Other | 19,647 (1.12) | 14,559 (1.19) |  |
| Unknown | 284,156 (16.22) | 107,210 (8.73) |  |
|  |  |  |  |
| ***Index of Multiple Deprivation*** |  |  |  |
| 1 (least deprived) | 290,170 (16.57) | 262,494 (21.38) |  |
| 2 | 288,664 (16.48) | 261,642 (21.31) |  |
| 3 | 279,202 (15.94) | 251,112 (20.45) |  |
| 4 | 259,566 (14.82) | 236,367 (19.25) |  |
| 5 (most deprived) | 235,220 (13.43) | 216,409 (17.62) |  |
| *Data missing/not recorded* | *398,563 (22.76)* |  |  |
|  |  |  |  |
| ***Smoking status*** |  |  |  |
| Non-smoker | 802,920 (45.84) | 615,650 (50.13) |  |
| Current smoker | 330,257 (18.86) | 259,855 (21.16) |  |
| Ex-smoker | 453,803 (25.91) | 352,519 (28.71) |  |
| *Data missing/not recorded* | *164,405 (9.39)* |  |  |
|  |  |  |  |
| ***Presence of co-morbidities*** |  |  |  |
| Type 2 diabetes | 262,713 (15.00) | 204,653 (16.67) | |
| Hypertension | 483,016 (27.58) | 356,543 (29.03) | |
| Coronary Heart Disease | 219,434 (12.53) | 178,006 (14.50) | |
| Obstructive Sleep Apnoea | 27,969 (1.60) | 20,503 (1.67) | |
| Asthma | 271,421 (15.50) | 212,379 (17.29) | |
| Chronic musculoskeletal condition | 381,461 (21.78) | 297,607 (24.23) | |
| Gastro-oesophageal reflux disease | 485,268 (27.71) | 358,630 (29.20) | |
| Liver disease | 37,430 (2.14) | 32,959 (2.68) | |
| Polycystic ovarian syndrome | 25,879 (1.48) | 17,734 (1.44) | |
| Fertility problems | 41,151 (2.35) | 28,599 (2.33) | |
| Depression | 456,930 (26.09) | 331,424 (26.99) | |
| Anxiety | 337,680 (19.28) | 249,076 (20.28) | |
| Idiopathic Intracranial Hypertension | 1,635 (0.09) | 1,268 (0.10) | |
|  |  |  | |
| ***Total co-morbidities*** |  |  |  |
| 0 | 413,602 (23.62) | 260,983 (21.25) |  |
| 1 | 477,600 (27.27) | 320,032 (26.06) |  |
| 2 | 387,124 (22.10) | 274,716 (22.37) |  |
| 3 | 248,112 (14.17) | 187,471 (15.27) |  |
| 4 | 132,761 (7.58) | 105,935 (8.63) |  |
| 5 | 59,770 (3.41) | 50,069 (4.08) |  |
| 6+ | 32,416 (1.85) | 28,818 (2.35) |  |
|  |  |  |  |

B&A=Black and Asian ethnic groups, BMI=Body Mass Index ^. a^Included in adjusted model. ^b^Medical codes indicating diagnosis with overweight or obesity where BMI category not specified.
