# Supplementary material for: Access to publicly funded weight management services in England using routine data from primary and secondary care (2007–2020): An observational cohort study
Source: PLoS Med. 2023 Sep 28;20(9):e1004282. doi: 10.1371/journal.pmed.1004282 (PMC10538857; doi:10.1371/journal.pmed.1004282)
Supplement: S5 Table — B&A, Black and Asian ethnic groups; BMI, body mass index. aIncluded in adjusted model. bMedical codes indicating diagnosis with severe and complex obesity where BMI category not specified. (DOCX) [file pmed.1004282.s012.docx]

**S5 Table: Characteristics of patients included in adjusted Poisson model versus those in full sample (bariatric surgery as outcome)**

|  | **Patients with severe and complex obesity with ≥5 years follow-up data (% of total)** | **Patients with severe and complex obesity with ≥5 years follow-up data and complete data for all variables^a^ (% of total)** |  |
| --- | --- | --- | --- |
|  |  |  |  |
| ***Total*** | 297,332 | 280,316 |  |
|  |  |  |  |
| ***Sex*** |  |  |  |
| Male | 125,147 (42.09) | 119,267 (42.55) |  |
| Female | 172,185 (57.91) | 161,049 (57.45) |  |
|  |  |  |  |
| ***Age group at diagnosis with severe and complex obesity*** |  |  |  |
| 18-24 | 11,173 (3.76) | 10,169 (3.63) |  |
| 25-34 | 26,016 (8.75) | 23,191 (8.27) |  |
| 35-44 | 43,386 (14.59) | 39,908 (14.24) |  |
| 45-54 | 60,640 (20.39) | 57,101 (20.37) |  |
| 55-64 | 66,549 (22.38) | 63,810 (22.76) |  |
| 65-74 | 54,893 (18.46) | 53,080 (18.94) |  |
| 75+ | 34,675 (11.66) | 33,057 (11.79) |  |
|  |  |  |  |
| ***Strategic Health Authority of GP practice*** |  |  |  |
| North East | 7,951 (2.67) | 7,186 (2.56) |  |
| North West | 52,436 (17.64) | 50,506 (18.02) |  |
| Yorkshire & the Humber | 12,627 (4.25) | 11,819 (4.22) |  |
| East Midlands | 9,836 (3.31) | 9,018 (3.22) |  |
| West Midlands | 36,765 (12.36) | 35,043 (12.50) |  |
| East of England | 29,996 (10.09) | 27,993 (9.99) |  |
| South West | 40,610 (13.66) | 38,189 (13.62) |  |
| South Central | 36,366 (12.23) | 34,228 (12.21) |  |
| London | 31,359 (10.55) | 29,034 (10.36) |  |
| South East Coast | 39,386 (13.25) | 37,300 (13.31) |  |
|  |  |  |  |
| ***Rural-urban classification of GP practice*** |  |  |  |
| Urban | 258,135 (86.62) | 244,184 (87.11) |  |
| Rural | 39,197 (13.18) | 36,132 (12.89) |  |
|  |  |  |  |
| ***Year of diagnosis with severe and complex obesity*** |  |  |  |
| 2007 | 99,441 (33.44) | 95,941 (34.23) |  |
| 2008 | 42,133 (14.17) | 40,134 (14.32) |  |
| 2009 | 32,030 (10.77) | 30,078 (10.73) |  |
| 2010 | 28,358 (9.54) | 26,603 (9.49) |  |
| 2011 | 25,679 (8.64) | 23,995 (8.56) |  |
| 2012 | 23,958 (8.06) | 22,231 (7.93) |  |
| 2013 | 21,740 (7.31) | 19,878 (7.09) |  |
| 2014 | 17,304 (5.82) | 15,555 (5.55) |  |
| 2015 | 6,689 (2.25) | 5,901 (2.11) |  |
|  |  |  |  |
| ***BMI category (kg/m^2^) at diagnosis with severe and complex obesity*** |  |  |  |
| 27.5-29.9 in B&A groups with T2DM diagnosed no more than 10 years prior to first eligible BMI measurement | 36,429 (12.25) | 35,603 (12.70) |  |
| 30.0-34.9 with T2DM diagnosed no more than 10 years prior to first eligible BMI measurement | 52,669 (17.71) | 51,338 (18.31) |  |
| 35.0-40.0 with weight related co-morbidity | 137,378 (46.20) | 129,061 (46.04) |  |
| 40.0 + | 70,713 (23.78) | 64,192 (22.90) |  |
| Medical codes^b^ | 143 (0.05) | 122 (0.04) |  |
|  |  |  |  |
| ***Ethnic group*** |  |  |  |
| White | 258,969 (87.10) | 245,123 (87.45) |  |
| Asian | 9,113 (3.06) | 8,445 (3.01) |  |
| Black | 7,263 (2.44) | 6,603 (2.36) |  |
| Mixed | 1,430 (0.48) | 1,301 (0.46) |  |
| Other | 2,713 (0.91) | 2,478 (0.88) |  |
| Unknown | 17,844 (6.00) | 16,366 (5.84) |  |
|  |  |  |  |
| ***Index of Multiple Deprivation*** |  |  |  |
| 1 (least deprived) | 49,899 (16.78) | 47,093 (16.80) |  |
| 2 | 58,437 (19.65) | 55,105 (19.66) |  |
| 3 | 61,865 (20.81) | 57,890 (20.65) |  |
| 4 | 62,890 (21.15) | 59,361 (21.18) |  |
| 5 (most deprived) | 64,054 (21.54) | 60,867 (21.71) |  |
| *Data missing/not recorded* | *187 (0.06)* |  |  |
|  |  |  |  |
| ***Smoking status*** |  |  |  |
| Non-smoker | 131,749 (44.31) | 131,667 (46.97) |  |
| Current smoker | 59,622 (20.05) | 59,585 (21.26) |  |
| Ex-smoker | 89,118 (29.97) | 89,064 (31.77) |  |
| *Data missing/not recorded* | *16,843 (5.66)* |  |  |
|  |  |  |  |
| ***Presence of co-morbidities*** |  |  |  |
| Type 2 diabetes | 146,114 (49.14) | 142,271 (50.75) | |
| Hypertension | 126,748 (42.63) | 122,398 (43.66) | |
| Coronary Heart Disease | 62,171 (20.91) | 60,322 (21.52) | |
| Obstructive Sleep Apnoea | 11,899 (4.00) | 11,354 (4.05) | |
| Asthma | 65,730 (22.11) | 63,054 (22.49) | |
| Chronic musculoskeletal condition | 99,615 (33.50) | 95,594 (34.10) | |
| Gastro-oesophageal reflux disease | 103,322 (34.75) | 98,641 (35.19) | |
| Liver disease | 13,763 (4.63) | 13,152 (4.69) | |
| Polycystic ovarian syndrome | 7,730 (2.60) | 6,919 (2.47) | |
| Fertility problems | 7,343 (2.47) | 6,674 (2.38) | |
| Depression | 105,265 (35.40) | 99,284 (35.42) | |
| Anxiety | 71,305 (23.98) | 67,452 (24.06) | |
| Idiopathic Intracranial Hypertension | 794 (0.27) | 742 (0.26) | |
|  |  |  |  |
| ***Total co-morbidities*** |  |  |  |
| 0 | 11,737 (3.95) | 9,946 (3.55) |  |
| 1 | 59,402 (19.98) | 53,902 (19.23) |  |
| 2 | 73,894 (24.85) | 69,354 (24.74) |  |
| 3 | 63,963 (21.51) | 61,184 (21.83) |  |
| 4 | 44,829 (15.08) | 43,395 (15.48) |  |
| 5 | 25,395 (8.54) | 24,739 (8.83) |  |
| 6+ | 18,112 (6.09) | 17,796 (6.35) |  |
|  |  |  |  |

B&A=Black and Asian ethnic groups, BMI=Body Mass Index. ^a^Included in adjusted model. ^b^Medical codes indicating diagnosis with severe and complex obesity where BMI category not specified.
