# Supplementary material for: Access to publicly funded weight management services in England using routine data from primary and secondary care (2007–2020): An observational cohort study
Source: PLoS Med. 2023 Sep 28;20(9):e1004282. doi: 10.1371/journal.pmed.1004282 (PMC10538857; doi:10.1371/journal.pmed.1004282)
Supplement: Supplementary file 15 [file pmed.1004282.s015.docx]

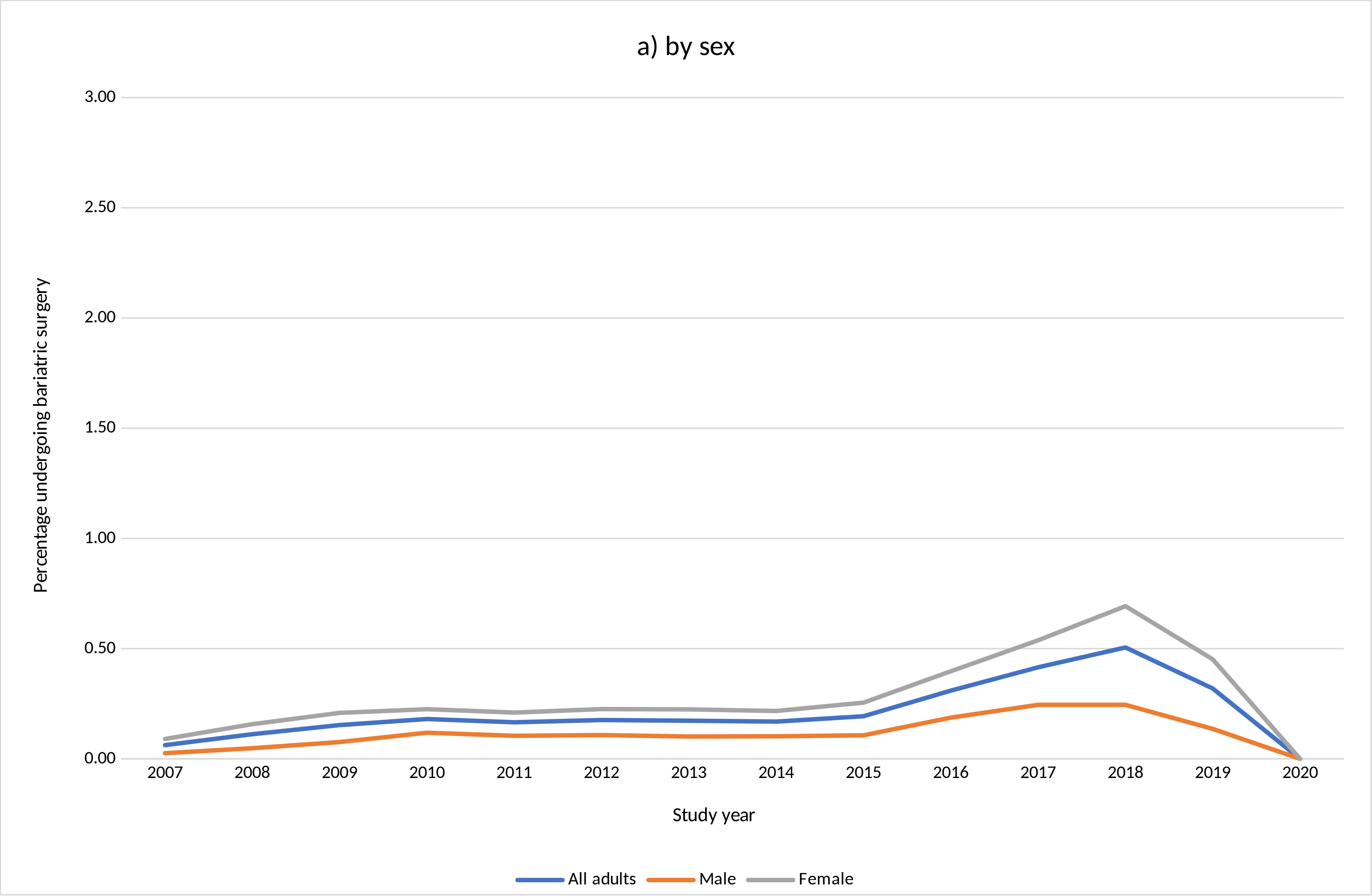
a) by sex
Percentage undergoing bariatric surgery
3.00
2.50
2.00
1.50
1.00
0.50
0.00
2007
2008
2009
2010
2011
2012
2013
2014
2015
2016
2017
2018
2019
2020
Study year
All adults
Male
Female

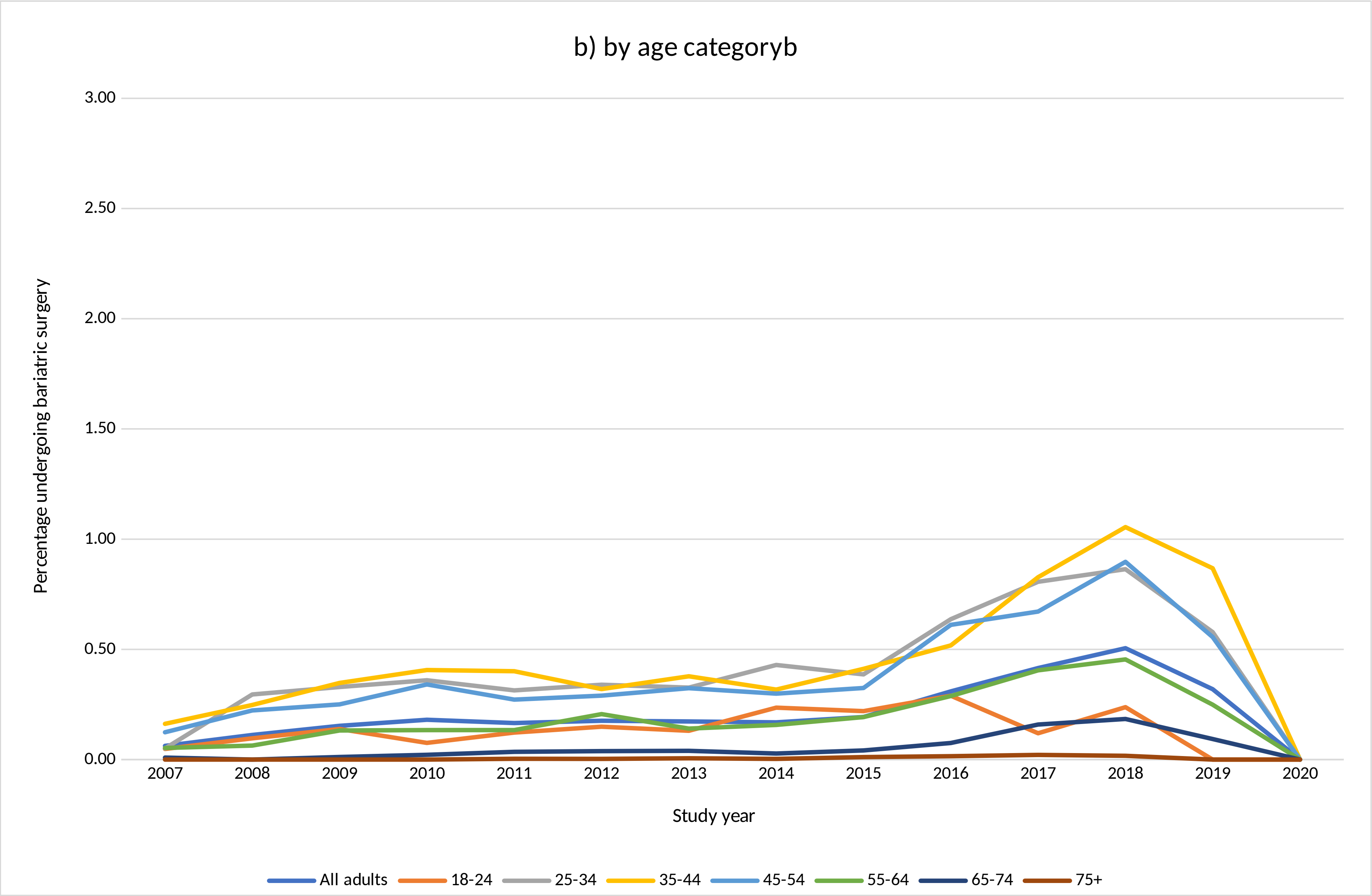
b) by age categoryb
Percentage undergoing bariatric surgery
3.00
2.50
2.00
1.50
1.00
0.50
0.00
2007
2008
2009
2010
2011
2012
2013
2014
2015
2016
2017
2018
2019
2020
Study year
All adults
18-24
25-34
35-44
45-54
55-64
65-74
75+

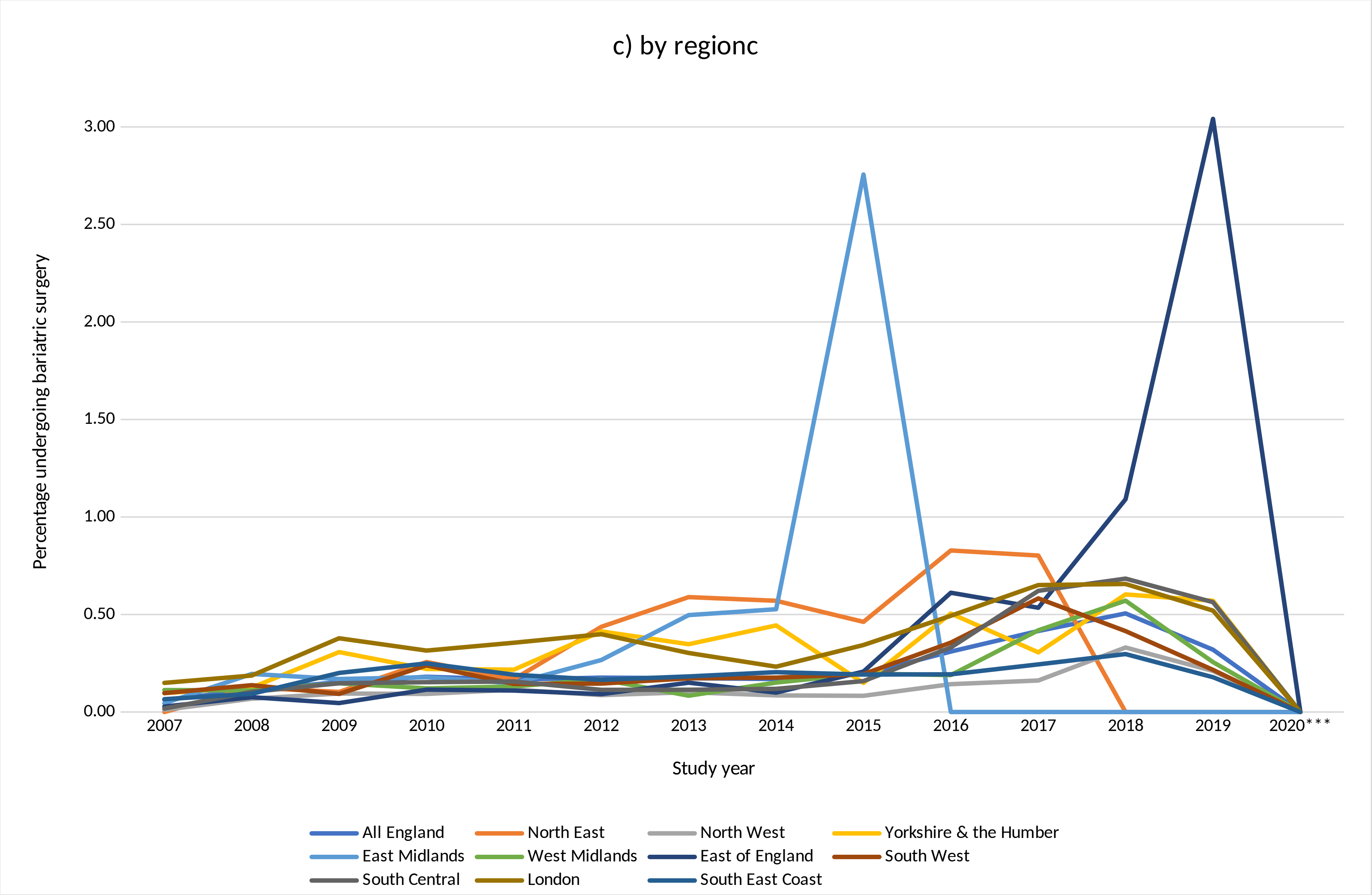
c) by regionc
Percentage undergoing bariatric surgery
3.00
2.50
2.00
1.50
1.00
0.50
0.00
2007
2008
2009
2010
2011
2012
2013
2014
2015
2016
2017
2018
2019
2020***
Study year
All England
North East
North West
Yorkshire & the Humber
East Midlands
West Midlands
East of England
South West
South Central
London
South East Coast
